# The catastrophic cost of TB care: Understanding costs incurred by individuals undergoing TB care in low-, middle-, and high-income settings – A systematic review

**DOI:** 10.1371/journal.pgph.0004283

**Published:** 2025-04-02

**Authors:** Olivia Alise D’Silva, Samantha Lancione, Oviya Ananthakrishnan, Angelina Addae, Suvesh Shrestha, Hannah Alsdurf, Kednapa Thavorn, Nompumelelo Mzizi, Anca Vasilu, Alexander Kay, Anna Maria Mandalakas, Alice Anne Zwerling

**Affiliations:** 1 University of Ottawa, School of Epidemiology and Public Health, Ottawa, Canada; 2 Department of Microbiology and Immunology, University of Western Ontario, London, Canada; 3 Vaccines Research & Development, GSK GlaxoSmithKline, Rockville, Maryland, United States of America,; 4 The Ottawa Hospital Research Institute, Ottawa Hospital, Ottawa, Canada; 5 Department of Pediatrics, Section on Retrovirology and Global Health, Baylor College of Medicine, Houston, Texas, United States of America; 6 Baylor College of Medicine Children’s Foundation Eswatini, Mbabane, Eswatini; 7 Clinical Infectious Disease Group, German Center for Infectious Research (DZIF) Clinical TB Unit, Research Center Borstel, Sülfeld, Germany; Federal University Birnin Kebbi, NIGERIA

## Abstract

Eliminating the burden of catastrophic costs experienced by individuals undergoing tuberculosis (TB) treatment is one of the World Health Organization (WHO) End TB Strategy targets. To help inform decisions on TB patient burden and cost-effective TB care, we conducted a systematic review to summarize current evidence around the burden of catastrophic costs incurred by individuals undergoing TB treatment and identified the main drivers of costs to aid in developing mitigation strategies. A literature search was performed in August 2024 using Embase, Web of Science, Scopus and Medline targeting studies using WHO, or WHO adapted patient costing questionnaires to measure direct (medical and non-medical) and indirect costs associated with TB care. Key cost data and patient baseline characteristics were extracted. The study protocol was registered in PROSPERO (Registration number: CRD42021293600). The systematic review included 76 studies; with 70% published over the last 5 years. Total mean costs per person for TB care ranged from $7.13 - $11,329 USD; pre-diagnostic costs ranged from $30.37 - $1,442 USD; and post-diagnostic costs ranged from $33.64 - $5,194 USD. Costs were consistently higher amongst persons with drug resistant TB (DR-TB) and those identified through passive case finding (PCF). Hospitalization and loss of income were the largest drivers of cost. Despite many countries offering free TB treatment, patients still incurred significant catastrophic costs. Our review suggests that active case finding, improving access to DR-TB testing, and adopting social protection interventions may help mitigate the burden of out-of-pocket expenditures incurred by people suffering with TB.

## Summary

Evidence before this study:

Economic evidence examining patient costs incurred during TB treatment has previously been limited; however, following the release of the WHO “End TB Strategy” goals, there has been a rapid explosion of literature in this space. A handful of systematic reviews on the burden on catastrophic costs for TB patients have been conducted previously but they have restricted their analysis to particular geographical settings, patient populations or phases of TB care. We found no systematic review which examined the burden of these costs for patients in all stages of TB care across differing geographical settings and amongst different patient populations.

Added value of this study:

Our study demonstrated that despite countries offering free or subsidized TB care to their citizens, patients and their families still incurred significant catastrophic costs with many individuals resorting to coping strategies to manage the cost of care. Patients incurred a significant burden of costs throughout all stages of TB care with post-diagnostic medical components, including hospitalization and non-TB medications, having the largest impact. In addition, costs were consistently higher amongst patients with DR-TB and those who were identified using passive case finding (PCF) methods suggesting that delayed diagnosis can influence both patient outcomes as well as the burden of costs incurred during care.

Implications of all the available evidence:

The evidence derived from this study highlights the need for increased use of active case finding to promote early detection and diagnosis of TB, improved access to drug resistance testing, and timely access to treatment. Our study found that post diagnostic medical costs continue to have a large influence on patient costs. This finding highlights the importance of financial mitigation strategies for clients after they are diagnosed and engaged in care.

## Introduction

In 2022, tuberculosis (TB) was the second leading infectious cause of mortality, following COVID-19^1^. The WHO reports that 50% of individuals impacted by TB incur catastrophic patient costs, defined as the total cost of TB care exceeding 20% of a household’s annual income pre-TB diagnosis[1]. The End TB Strategy developed by the WHO has as a central aim the goal to eliminate the burden of catastrophic costs for TB patients and their households by 2030[2]. But in order to achieve this goal we must have a clear understanding of how and where in the diagnostic and treatment pathways clients incur significant costs.

Patient costs can be incurred at any stage of treatment, pre-diagnostic or post-diagnostic, through direct medical costs such as consultations by medical professionals, diagnostic testing or medications, and direct non-medical costs such as transportation, accommodations and food. Indirect costs resulting from time lost during illness and loss of income while seeking care can also be significant[1,3,4]. For many, the cost burden catastrophically impacts their overall household income resulting in significant financial as well as social consequences. The financial burden associated with TB care has been found to deter individuals from seeking care and can lead to delayed diagnosis, poor adherence, and worsened TB outcomes[5,6]. While many countries offer free TB diagnosis and treatment through public health systems, patients and households with TB still incur severe costs associated with care[3,7].

Prior to 2015, economic evidence on patient costs associated with TB care was sparse. Since then, WHO has supported numerous national surveys to better understand the burden of patient-incurred costs. This coupled with the “Tuberculosis Patient Cost Surveys: A Handbook”, from WHO provides a standardized method for assessing TB patient costs[1,7,8]. Previous systematic reviews found that a large proportion of patients incur catastrophic costs while receiving TB care. Key populations— patients with drug-resistant TB (DR-TB) or human immunodeficiency virus (HIV), typically bear a higher burden of costs[3,4]. Yet these studies have not provided a detailed understanding of the costs associated with TB care or factors influencing these costs. Research examining direct and indirect costs incurred throughout all stages of care is limited. To our knowledge, no systematic review to date has reported on patient costs incurred during all stages of TB-care, across all global socioeconomic settings, with prior reviews restricted to analyses of catastrophic costs at specific stages of treatment, specific geographic regions, or among key population settings[3,4,9]. Thus, we conducted a systematic review to help broaden the current understanding of catastrophic costs by examining all costs incurred by patients during TB care in low-, middle-, and high-income country settings. Key costing data from the included studies was categorized as direct medical, direct non-medical, and indirect cost components, and main cost drivers were identified.

## Methods

A systematic review was conducted following Preferred Reporting Items for Systematic Reviews and Meta-Analyses (PRISMA) guidelines to understand the burden of costs for TB patients. Literature searches were performed in August 2024 in Embase, Scopus, Medline, and Web of Science databases. The studies that were included were published between 2011 and 2024. The search strategy contained no restrictions on language, or geography (S1 Text).

### Inclusion criteria

Inclusion criteria consisted of primary research studies that collected and reported on TB patient-incurred costs using a WHO-approved or WHO-adapted patient costing survey (WHO – Tuberculosis Patient Costing Surveys: A Handbook, StopTB, USAID or the TB|CTA – Tool to Estimate Patient Costs) to collect costs[8,10,11]. We included patient populations with latent tuberculosis infection (LTBI), active TB disease, DR-TB, or multi-drug-resistant TB (MDR-TB) as well as with HIV-associated TB.

### Exclusion criteria

Studies were excluded if they used a unique costing survey not based on the WHO approach or if they did not report patient costs. Abstracts, conference posters, letters, and systematic reviews were also excluded.

**Fig 1:**
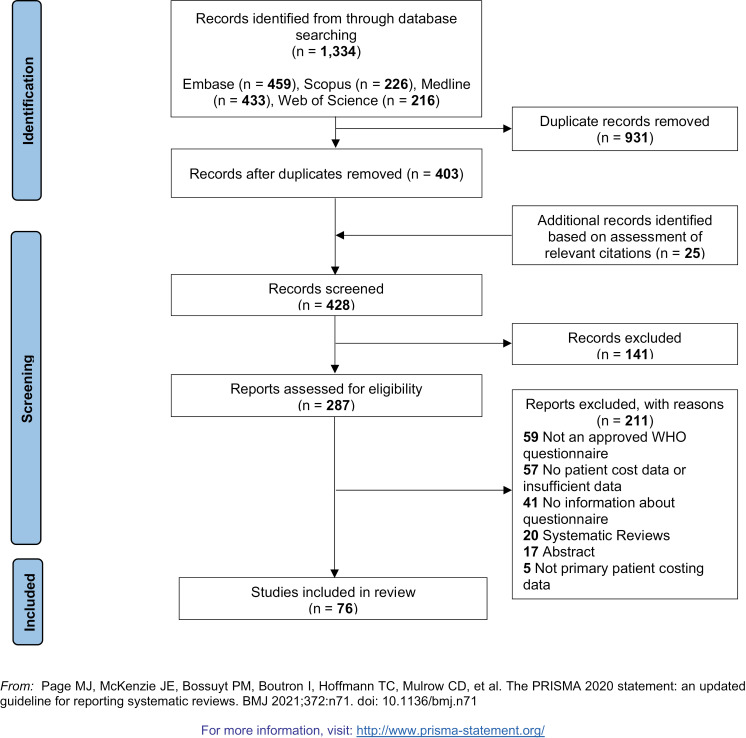
Shown above is the PRISMA flow diagram for the search strategy conducted for this systematic review. It highlights the three main sections of the literature search – the identification phase, the screening phase and the inclusion phase. In addition, it also shows the number of studies that were excluded and their exclusion reasons as well as the number of additional records that were identified through examination of grey literature and added in to the review.

Studies were independently evaluated for inclusion by two reviewers (OD and SL). Discrepancies were resolved by discussion and consensus or by a third reviewer (AZ) where necessary. References from systematic reviews were examined to identify additional studies for inclusion. Included studies are shown in Fig 1 with detailed reasons for exclusions[12]. Key study and patient characteristics and costing data were independently extracted in duplicate by three reviewers (OD, SL, and OA) with discrepancies resolved by discussion. Reporting quality assessment of studies was done using an adapted CHEERS checklist (S1 Checklist).

The primary study outcome was the total cost incurred by TB patients. Pre- and post-diagnostic costs were obtained and stratified by direct medical, direct non-medical, and indirect costs. Key cost components are listed and defined in the Appendix (S1 Table). The secondary outcome was the proportion of patients suffering from catastrophic costs based on the WHO definition. All costs were reported in 2023 USD using World Bank inflation and currency data. If there was missing data such as the year of cost valuation or original currency, reviewers would first attempt to contact the authors for clarification on the matter. If the information could not be confirmed with study authors, it was assumed that costs were collected in the local currency of the country where the study was conducted and valued at the final year of study. We reported both mean and median costs depending on how costs were reported which varied across studies. We also reported the average total cost of TB care across countries by calculating the average total costs reported in each study by country. Overall and individual cost components for direct medical, direct non-medical and indirect costs were compared across geographic settings, patient demographics, treatment stages, and economic standings to understand the burden across different groups as well as identify main drivers of costs.

Weighted averages were calculated by compiling reported patient costs for each subgroup, dividing the cost by patient population size and summing the costs together. Since costs were reported differently across studies both weighted mean and weighted medians were calculated for each sub-group of interest. These were used to understand the differences in patient costs across various subgroups including TB types (DS-TB vs DR/MDR-TB vs TB/HIV coinfection), geographic setting (WHO regions – African Region (AFRO), Eastern Mediterranean Region (EMRO), South-East Asia Region (SEARO), Western Pacific Region (WPRO), European Region (EURO), and the Pan American Health Organization (PAHO)), and TB burden settings (low-TB vs high-TB). TB burden settings were defined using the WHO Global List of High Burden Countries for Tuberculosis (TB), TB/HIV and multidrug/rifampicin-resistant TB (MDR/RR-TB), 2021 – 2025[13].

This study has been registered in PROSPERO (Registration number: CRD42021293600).

## Results

### PRISMA

Our search identified 1,334 studies, with an additional 25 articles found through manual searches. After 931 duplicates were removed, title and abstracts for 428 studies were reviewed, with 287 studies identified for full text reviews. Two studies were excluded due to no members of our team speaking the language in which they were published (Spanish). The remaining 285 studies underwent full-text review, and 76 studies were included in this review (Fig 1) [14–89].

### Study characteristics

Key study characteristics for the included studies are outlined in Table 1. Of the 76 studies, 53 (70%) were published within the last five years (2019 – 2024) (S1 Fig) with all studies but one conducted in low- or middle-income countries based on World Bank Country classifications. Studies were conducted across rural and urban settings with a large proportion conducted in Asian (47.4%) and African countries (37.2%) (S2 Fig). Study populations consisted mostly of adult patients; 9 studies reported data on children <15 years of age, 36 studies (61%) included patients with HIV and 1 study included patients with diabetes. A total of 32 studies (42%) included patients with DR-TB or MDR-TB and only 1 study included patients with LTBI (1%). Where reported studies were conducted in either public health care settings (n=39), or a combination of public and private health care settings (n=31) (Table 1).

**Fig 2:**
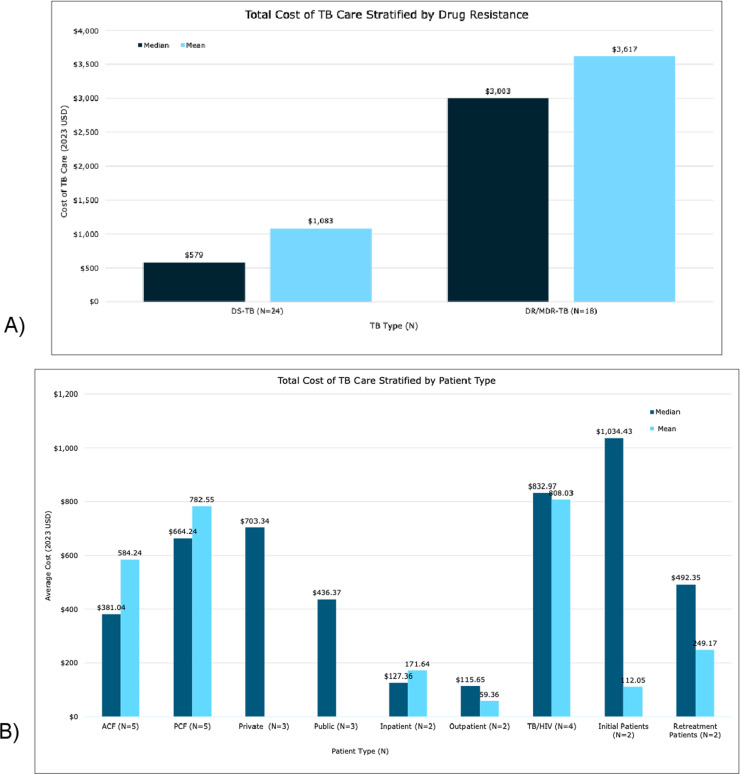
Shown above is the average total cost of TB care when stratified across TB type as represented in Fig 2A and when stratified by patient type as represented in Fig 2B. Fig 2A has the mean and median total average cost of TB care and reflects the differences in costs for patients who have DS-TB compared to patients with DR/MDR-TB. Fig 2B also presented the mean and median total average cost of TB care but instead reflects the difference in costs for differing subgroups of patient types including patients identified using ACF and PCF methods, patients in private and public health settings, inpatients and outpatients, initial and retreatment TB patients as well as patients with TB/HIV co-infection.

**Table 1:** Study characteristics of included studies.

*Author name, Publication year*	*Study Type*	*Year of study conduct*	*Study country (Urbanization status)*	*Study population*	*Sample size*	*Collected currency, Year of cost valuation*	*Health Centre Category*
Aia, 2022**[14]**	Cross-sectional	2018 -2019	Papua New Guinea (NA)	TB patients, adults and children	n = 1000 (MDR-TB, n = 19, TB/HIV, n = 49)	Papua New Guinean Kina (PGK), 2019	Public
Assebe, 2020**[15]**	Cross-sectional	2016 – 2019	Ethiopia (Urban, Rural)	TB patients, HIV^+^/- adult	n = 787 (DR-TB=57)	Ethiopian Birr (ETB), 2019	Public
Aung, 2021**[16]**	Cross-sectional	2015 – 2016	Myanmar (NA)	TB patients, adult	n = 967 (MDR-TB=66, DS-TB =901)	Myanmar Kyat (MMK), 2015	NA
Bengey, 2023**[17]**	Longitudinal	2018 – 2019	Nepal (NA)	New or relapsed DS-TB patients, adults	n = 221	Nepalese Rupees (NPR), 2019	Public
Bogdanova, 2019**[18]**	Cost minimization analysis	2007 – 2009, 2011 – 2012	Russian Federation (Urban)	MDR-TB patients, adult	n = 295	Russian Rubles (RUB), 2014	Public
Chandra, 2021^(1)^**[20]**	Longitudinal	2019	North India (Urban, Rural)	New DS-TB patients, adults	n = 110	Indian Rupee (INR), 2019	NA
Chandra, 2021^(2)^**[19]**	Cross-sectional	2019	North India (Urban, Rural)	New DS-TB patients, adults	n = 109	Indian Rupee (INR), 2019	NA
Chatterjee, 2023**[21]**	Cohort	2019 - 2021	India (Urban, Rural)	DS-TB patients, adults	n = 260	Indian Rupees (INR), 2020	Public & Private
Chatterjee, 2024**[22]**	Cohort	2019 - 2022	India (Urban, Rural)	DS-TB patients, adults	n = 529	Indian Rupees (INR), 2022	Public & Private
Chittamany, 2020**[23]**	Cross-sectional	2018	Lao People’s Democratic Republic (NA)	TB patients	n = 848 (Nationally representative sample = 725, n=717 DS-TB, n=8 DR-TB; Additional sample = 123, n=30 DR-TB, n=123 TB-HIV)	Lao Kip (LAK), 2018	Public
Ciobanu, 2024**[24]**	Cross-sectional	2016 - 2017	Moldova (Urban, Rural)	New or relapse RR-TB patients, adults	n = 430	Moldovan Leu (MDL), 2016	Public & Private
Collins, 2018**[25]**	Cross-sectional	2013	Ethiopia (Urban)	New MDR-TB patients	n=169	Ethiopian Birr (ETB), 2013	Public
Deribew, 2021**[26]**	Cross-sectional	2020 – 2022	Ethiopia (Rural)	TB patients	n = 433	Ethiopian Birr (ETB), 2022	Public & Private
Diallo, 2022**[27]**	Cross-sectional	2020	Burkina Faso (NA)	TB patients	n = 465 (DS-TB, n = 457, DR-TB, n = 8)	West African CFA Franc (CFA), 2020	Public
Dinh, 2023**[28]**	Prospective Cohort	2020 - 2022	Vietnam (Urban)	DS-TB patients, adults	n = 212	Vietnam Dong (VND), 2022	NA
De Siqueria Filha, 2018**[29]**	Pragmatic clinical trial	2015	Brazil (Urban)	New TB patients, HIV-positive adults	n=31 (TB/HIV=26, LTBI/HIV=5)	Brazilian Real (BRL), 2015	Public
Devoid, 2022**[30]**	Observational	2017 – 2020	The Gambia (Urban, Rural)	DS-TB patients, adults	n = 244	Gambian Dalasi (GMD), 2018	Public
Ellaban, 2021**[31]**	Prospective	2019 – 2020	Egypt (NA)	New TB patients, adults	n=151	Egyptian Pound (EGP), 2019	Public & Private
Florentino, 2022**[32]**	Cross-sectional	2016 – 2017	The Philippines (Urban, Rural)	TB patients	n = 1,912 (urban DS-TB – n = 786, rural DS-TB – n = 806, DR-TB – n = 320)	Philippine Peso, 2017	Public
Fuady, 2020**[34]**	Cohort	2016	Indonesia (Urban, Rural, Suburban)	New TB patients, adults	n=252	Indonesian Rupiahs (IDR), 2016	Public
Fuady, 2020**[33]**	Cross-sectional	2016	Indonesia (Urban, Rural, Suburban)	New TB patients, adults	n=346 (DS-TB=282, MDR-TB=64)	Indonesian Rupiahs (IDR), 2015	Public
Fuady, 2018**[35]**	Cross-sectional	2016	Indonesia (Urban, Rural, Suburban)	New DS-TB or MDR-TB patients, adults	n=346 (DS-TB=282, MDR-TB=64)	Indonesian Rupiahs (IDR), 2015	Public
Gadallah, 2022**[36]**	Cohort	2019	Egypt (Urban, Rural)	DS-TB patients, adults and children	n = 257	Egyptian Pounds, 2019	Public & Private
Getahun, 2016**[37]**	Cross-sectional	2015	Ethiopia (Urban, Rural, Homeless)	New TB patients, adults	n=576 (MDR-TB=16)	Ethiopian Birr (ETB), 2015	Public & Private
Gospodarevskaya, 2014**[38]**	Cross-sectional	2012	Bangladesh (subdistricts), Tanzania (Urban, Rural)	TB patients, adults	n=190 (Bangladesh=96, Tanzania=94)	Tanzanian Shillings, Bangladesh Takas (TZS, BDT), 2012	Public
Gurung, 2021**[39]**	Longitudinal	2018 – 2019	Nepal (Urban, Rural)	New, retreated or relapsed DS-TB patients, adults	n=221	Nepal Rupee (NPR), 2018	Public & Private
Gurung, 2019**[40]**	Cross-sectional	2018	Nepal (NA)	New, retreated or relapsed DS-TB patients, adults	n=99	Nepal Rupee (NPR), 2018	Public
Kaswa, 2021**[41]**	Cross-sectional	2019	The Democratic Republic of Congo (Urban, Rural)	TB patients, adults and children	n = 1,108 (DR-TB, n = 197, DS-TB, n = 911)	Congolese Francs, 2019	Private & Public
Kilale, 2022**[42]**	Cross-sectional	2019	Tanzania (Urban, Rural)	TB patients, adults and children	n = 777 (DS-TB = 752, DR-TB = 25)	Tanzanian Shillings, 2019	Public
Kirubi, 2021**[43]**	Cross-sectional	2017	Kenya (Urban, Rural)	DS-TB patients	n=1071	Kenyan Shillings (KSH), 2020	Public & Private
Lourerio, 2023**[44]**	Cross-sectional	2016 – 2018	Brazil (Urban, Rural)	TB patients, adults	n = 62	Brazilian Reals (BRL), 2016	Public
Lu, 2020**[45]**	Cross-sectional	2014-2015	China (Urban)	DS-TB patients	n=248	Chinese Yuan (CNY), 2015	NA
Maciel, 2023**[46]**	Cross-sectional	2019 – 2021	Brazil (Urban)	DS-TB or DR-TB patients	n = 603 (DS-TB = 538, DR-TB = 65)	Brazilian Real (BRL), 2021	Public & Private
Mafirakureva, 2023 1**[47]**	Cross-sectional	2021	Cameroon, Uganda (NA)	Caregivers of TPT patients, children	Cameroon=57, Uganda=47	Central African Franc (CFA), Ugandan Shillings (UGX), 2021	NA
Mafirakureva, 2023 2**[48]**	Cross-sectional	2020 – 2021	Cameroon, Kenya (NA)	Caregivers of TB patients, children	n = 56 (Cameroon=26, Kenya=30)	Central African Franc (CFA), Kenyan Shilling (KES), 2021	Public & Private
Manyazewal, 2022**[49]**	Randomized control hybrid	2021	Ethiopia (NA)	DS-TB patients, adults	n = 109	Ethiopian Birr (ETB), 2021	Public & Private
Martinez, 2024**[50]**	Cross-sectional	2021	Colombia (NA)	DS-TB or DR-TB patients	n = 1,065 (DS-TB=1,035, DR-TB=30)	Colombian Pesos (COP), 2021	Public & Private
Mauch, 2013^(1)^**[53]**	Cross-sectional	2010	Ghana, Viet Nam, Dominican Republic (Urban, Rural)	New DS-TB patients, ≥15 years	n=543 (Ghana=135, Viet Nam=258, Dominican Republic=150)	Dominican Republic Pesos (DOP), Ghanaian Cedi (GHC), Vietnamese Dong (VND), 2009	Public & Private
Mauch, 2011**[51]**	Cross-sectional	2008	Kenya (Urban, Rural)	New or re-treatment TB patients, ≥ 15 years	n=208	Kenyan Shilling (KSH), 2008	Public
Mauch, 2013^(2)^**[52]**	Cross-sectional	2009	Dominican Republic (Urban, Rural)	New or re-treatment TB patients, adults under the age of 65	n=198 (New TB=150, Retreatment= 28, MDR-TB=20)	Dominican Republic Pesos (DOP), 2009	Public & Private
McAllister, 2020**[54]**	Cross-sectional	2017-2019	Indonesia (NA)	New TB patients, adults	n=469	Indonesian Rupiahs (IDR), 2017	Public & Private
Medeiros, 2023**[55]**	Prospective	2019 – 2021	Brazil (Urban)	MDR-TB patients, adults	n = 72	Brazilian Reals (BRL), 2020	Public & Private
Morishita, 2016**[56]**	Cross-sectional	2014	Cambodia (NA)	New DS-TB patients, adults	n=208	Cambodian Riel (KHR), 2014┴	Public
Mudzengi, 2017**[57]**	Cross-sectional study nested within a cluster randomized trial—the MERGE trial.	2013	South Africa (NA)	New TB, HIV, or TB/HIV patients, adults	n=454 (TB/HIV=116, TB=40, HIV= 298)	South African Rand (ZAR), 2013	Public
Muniyandi, 2020**[58]**	Prospective cross-sectional	2018	South India (Urban)	DS-TB patients	n=384	Indian Rupee (INR), 2018	Public
Muttamba, 2020**[59]**	Cross-sectional survey design with retrospective data collection and projections.	2017	Uganda (NA)	DS-TB or MDR-TB patients, children and adults	n=1178 (DS-TB=1134, MDR-TB=44)	Ugandan Shillings (UGX), 2017	Public
Nguyen, 2024**[60]**	Longitudinal	2020 - 2022	Vietnam (Urban)	New DS-TB patients, adults	n = 110 (Private=50, NTP=60)	Vietnamese Dong (VND), 2022	Public & Private
Nhung, 2018**[61]**	National-level cross-sectional survey with retrospective data collection and projection	2016	Vietnam (NA)	DS-TB or MDR-TB patients, children and adults	n=735 (DS-TB=677, MDR-TB=58)	Vietnamese Dong (VND), 2015	Public & Private
Panda, 2024**[62]**	Longitudinal	2021 - 2022	India (Urban, Rural)	New DS-TB patients	n = 160	Indian Rupees (INR), 2022	Public & Private
Pedrazzoli, 2018**[63]**	Nationally representative survey	2016	Ghana (Urban, Rural)	TB patients, adults	n=691 (DS-TB=625, MDR-TB=66)	Ghanaian Cedi (GHC), 2016	Public
Pedrazzoli, 2021**[64]**	Cross-sectional	2016	Ghana (Urban, Rural)	TB patients, adults	n=690	Ghanaian Cedi (GHC), 2016	Public
Pham, 2023**[65]**	Longitudinal prospective cohort	2020 – 2021	Vietnam (Urban)	MDR-TB patients, adults	n = 93	Vietnamese Dong (VND), 2022	Public & Private
Prasanna, 2018**[66]**	Cross-sectional quantitative and in-depth qualitative interviews	2016-2017	South India (Urban, Rural)	New or re-treatment DS-TB patients	n=102	Indian Rupee (INR), 2017	Public & Private
Ramma, 2015**[67]**	Cross-sectional cost-of-illness survey	2013	South Africa (Urban, Rural)	RR-TB or MDR-TB patients, adults	n=134	South African Rand (ZAR), 2013	Public
Razzaq, 2022**[68]**	Cross-sectional	2017 – 2018	Pakistan (Urban)	TB patients, adults	n=516	Pakistani Rupee, 2017	Public & Private
Rupani, 2020**[69]**	Descriptive cross-sectional study	2019	Western India (Rural, Suburban)	DS-TB patients, adults	n=458	Indian Rupee (INR), 2019	Public
Rupani, 2022**[70]**	Cross-sectional	2019 – 2021	India (Urban, Rural)	TB/HIV co-infection patients, adults	n=234	Indian Rupee (INR), 2020	Public
Shin, 2020**[71]**	Cross-sectional	2012-2017	Malawi (Rural)	Inpatients receiving TB care, adults	Primary population: Inpatients being treated for TB (n = 197); Comparison population: Outpatients being treated for TB (n=156) and outpatients consecutive adults who were recently diagnosed with HIV and undergoing screening for active TB (n=1530)	Malawian Kwacha (MWK), 2017	Public
Stracker, 2019**[72]**	Cross-sectional	2017-2018	South Africa (Rural)	New TB patients, adults	n=327 TB positive, n=263 Xpert negative	South African Rand (ZAR), 2018	Public
Sweeney, 2018**[73]**	Patient costing study nested within the TB FastTrack study, a pragmatic, cluster randomised trial	2014 – 2015	South Africa (Rural, Suburban)	Ambulant HIV-positive patients with no TB care, adults	n=66	South African Rand (ZAR), 2015	Public
Timire, 2021**[74]**	Nationally representative health facility-based survey	2018	Zimbabwe (Urban, Rural)	DS-TB or DR-TB patients	n=860 (DS-TB=851; DR-TB=49)	South African Rand^**^ (ZAR), 2018	Public
Tomeny, 2020**[75]**	Cross-sectional	2016	Philippines (NA)	TB patients, ≥16 years	n=194	Philippine Peso (PHP), 2016	Public & Private
Trajman, 2016**[76]**	Cross-sectional costing study	2013-2014	Brazil (Urban, Peri-urban)	TB patients	n=126	Brazilian Real (BRL), 2014	Public & Private
Ukwaja, 2013**[77]**	Cross-sectional	2011	Nigeria (Rural)	New TB patients, ≥15 years old	n=452	Nigeria Naira (NGN), 2011	Public & Private
Ukwaja, 2013**[78]**	Cross-sectional	2011	Nigeria (Urban, Rural)	New TB patients, ≥15 years old	n=452	Nigeria Naira (NGN), 2011	Public & Private
Van der Hof, 2016**[79]**	Cross-sectional	2012-2013	Ethiopia (Urban)	DS-TB or MDR-TB patients	n=603 (TB=197, MDR-TB=406) Ethiopia- 25 TB and 169 MDR-TB patients; Indonesia – 118 TB and 143 MDR-TB patients; Kazakhstan – 54 TB and 94 MDR-TB patients	Ethiopian Birr, Indonesia Rupiah, Kazakh Tenge (ETB, IDR, KZT), 2013	Public
Viney, 2019**[80]**	Cross-sectional health-facility based survey	2016-2017	The Democratic Republic of Timor Leste (Urban, Rural)	TB patients	n=457	US Dollar (USD), 2017	Public
Viney, 2021**[81]**	Cross-sectional	2017 – 2019	Solomon Islands (Urban, Rural)	TB patients, adults and children	n = 183	Solomon Island Dollar, 2019	Public
Vo, 2021**[82]**	Cross-sectional	2018 – 2019	Viet Nam (Urban)	DS-TB patients, adults	n = 98 (ACF, n = 52. PCF, n = 46)	Viet Nam Dong, 2019	Public & Private
Walcott, 2020**[83]**	Retrospective	2017	Uganda (Urban)	New TB patients, adults	n=196	Uganda Shillings (UGX), 2017	Public & Private
Wang, 2020**[84]**	Cross-sectional	2018	China (Urban, Rural)	MDR-TB patients	n=161	Chinese Yuan (CNY), 2018	Public
Wang, 2023**[85]**	Cross-sectional	2018 – 2019	China (Urban, Rural)	MDR-TB patients	n = 180	Chinese Yuan (CYN), 2019	Public
Yamanaka, 2024 (1)**[86]**	Longitudinal	2018 - 2020	Philippines (Urban, Rural)	DS-TB or DR-TB, adults	n = 530 (DS-TB = 443, DR-TB = 87)	Philippine Pesos (PHP), 2020	Public
Yamanaka, 2024 (2) **[87]**	Longitudinal	2018 – 2020	Philippines (Urban, Rural)	DS-TB or DR-TB, adults	n=455 (TB=324, TB-DM=121)	Philippine Pesos (PHP), 2020	Public
Yang, 2020**[88]**	Cross-sectional	2018	China (Urban, Rural)	RS-TB or RR-TB patients	n=672 (RS=586, RMR=30 MDR=56)	Chinese Yuan (CNY), 2017	Public
Youngkong, 2024**[89]**	Cross-sectional	2019 - 2021	Thailand (NA)	DS-TB or DR-TB patients	n = 1,400 (DS-TB = 1,382, DR-TB = 18)	Thai Baht (THB), 2021	Public & Private

Abbreviations: *TB – Tuberculosis, HIV – Human Immunodeficiency Virus, DR-TB – Drug-resistant TB, MDR-TB – Multi-drug resistant TB, DS-TB – Drug Sensitive TB, LTBI – Latent TB Infection, DOTs – Directly observed treatment, NGO – Non-governmental organization, PCF – Passive case finding, ACF – Active case finding, NTP - National TB Program, TB-DM – Tuberculosis and diabetes, RR-TB – Rifampicin-resistant TB, RS-TB – Rifampicin-sensitive TB,RMR – Rifampicin mono-resistant.*

** - study collected costs in South African Rand despite being conducted in Zimbabwe, ┴ ***- costs were originally reported in 2011 – 2014 USD.***

### Total patient costs

Total patient costs were reported in 60 studies (S2 Table). Total cost per person for DS-TB patients ranged from a mean of $7.13 to $4,161 and a median of $7.00 to $2,443. In contrast, DR-TB total patient costs were considerably higher ranging from a mean of $3,075 to $11,329 and a median of $342 to $7,714.

When comparing the *average total cost* of TB care (Fig 2A), DR-TB patients incurred the largest burden of costs (mean: $3,617, median: $3,003) compared to DS-TB patients (mean: $1,083, median: $579). The weighted total mean cost for DR-TB patients was $8,947, and the weighted median was $3,799, while the weighted total mean for DS-TB was $1,259, with a weighted median of $187.

In patients with DS-TB, cost ranges for differing patient populations, such as PLHIV, inpatients versus outpatients, and patients identified using different case finding methods like ACF compared to PCF were examined and are highlighted in Fig 2B. *Average total costs* of TB care were highest in initial TB patients (mean: $112, median:$ 1,034), and patients with HIV-associated TB (mean: $808, median: $833). Patients coming in for TB re-treatment were found to have lower costs compared to initial TB patients (mean: $249, median: $492). Patients identified via PCF methods (mean: $783, median: $664) had higher costs compared to patients identified using ACF methods (mean: $584, median: $381). Insignificant differences were seen when comparing the average cost of TB care between other patient subgroups.

### Pre-diagnostic patient costs

Total costs of pre-diagnostic care for patients were reported in 18 studies. Pre -diagnostic costs were relatively similar for MDR-TB patients (median $30.06 to $134) compared with DS-TB patients (median $7.30 to $168). Pre-diagnostic costs were divided into direct medical costs, direct non-medical costs, and indirect costs (Table 2). Medical costs were slightly higher than non-medical costs, ranging from a mean of $1.65 to $1,909 and a median of $7.61 to $113 (S2 Table). Consistent with earlier findings, direct medical costs were highest for patients with DR-TB, (mean: $1.65-$681, median: $8.42-$113) compared to DS-TB patients (mean: $3.42-$176, median: $8.11-$63.63). When summarized across studies weighted medians for DR-TB were $94.87, and among DS-TB a weighted median of $36.67 and weighted mean of $392.88 (Table 3). Factors that had the largest impact on the direct medical costs’ patients incurred during the pre-diagnostic phase were medications, diagnostic imaging and hospitalization. These cost components were highest for inpatients, patients with DR-TB and those identified using PCF methods.

**Table 2:** Total direct and indirect costs incurred by patients during the pre-diagnostic phase of TB care.

	*Direct Costs*													*Indirect Costs*		
	*Total*						*Medical*			*Non-Medical*						
***TB Type (DS-TB, DR-TB, MDR-TB)***																
***Aia, 2022*[** **14** **]**							*DS-TB*	Mean (95% CI): $10.57 (8.09 – 12.93)		*DS-TB*		Mean (95% CI): $26.19 (22.70 – 29.45)				
							*MDR-TB*	Mean (95% CI): $18.32 (10.34 – 46.98)		*MDR-TB*		Mean (95% CI): $46.87 (24.05 – 86.32)				
							*Total*	Mean (95% CI): $10.68 (8.32 – 13.04)		*Total*		Mean (95% CI): $26.41 (23.04 – 29.78)				
***Aung, 2021*[** **16** **]**							*DS-TB*	Median: $10.22 (0 – 685.92)		*DS-TB*		Median: $4.19 (0 – 226.15)				
							*MDR-TB*	Median: $17.93 (4.27 – 71.89)		*MDR-TB*		Median: $4.19 (0.85 – 29.33)				
							*Total*	Median: $10.22 (0 – 685.92)		*Total*		Median: $4.19 (0 – 219.45)				
***Chittamany, 2020*[** **23** **]**							*DS-TB*	Median: $38.00 (5.70 – 168.17)		*DS-TB*		Median: $10.45 (3.80 – 37.06)				
							*DR-TB*	Median: $67.46 (22.80 – 69.36)		*DR-TB*		Median: $38.01 (22.80 – 49.41)				
							*Total*	Median: $38.95 (5.70 – 168.17) x		*Total*		Median: $10.45 (3.80 – 37.06)				
***Collins, 2018*[** **25** **]**	*MDR-TB*		Median (Range): $133.98 (71.46 – 341.21)											*MDR-TB*	Median (Range): $0 (0 – 14.29)	
***Diallo, 2022*[** **27** **]**							*DS-TB*	Mean (95% CI): $43.79 (27.38 – 60.28)		*DS-TB*		Mean (95% CI): $10.32 (5.59 – 15.09)				
							*DR-TB*	Mean (95% CI): $46.97 (8.28 – 102.22)		*DR-TB*		Mean (95% CI): $3.06 (1.83 – 4.29)				
							*Total*	Mean (95% CI): $43.91 (27.63 – 60.14)		*Total*		Mean (95% CI): $10.20 (5.54 – 14.86)				
***Florentino, 2022*[** **32** **]**	*Urban DS-TB*		Mean (SD): $21.94 (23.69)				*Urban DS-TB*	Mean (SD): $18.90 (19.72)		*Urban DS-TB*		Mean (SD): $3.03 (5.72)				
	*Rural DS-TB*		Mean (SD): $22.75 (39.33)				*Rural DS-TB*	Mean (SD): $19.14 (35.56)		*Rural DS-TB*		Mean (SD): $3.62 (9.10)				
	*DR-TB*		Mean (SD): $30.81 (332.57)				*DR-TB*	Mean (SD): $23.34 (243.42)		*DR-TB*		Mean (SD): $7.58 (89.62)				
	*Total*		Mean (SD): $22.75 (54.85)				*Total*	Mean (SD): $19.14 (45.28)		*Total*		Mean (SD): $3.62 (12.37)				
***Fuady, 2018*[** **35** **]**	*DS-TB*		Median (IQR): $12.25 (3.34 – 23.38)											*DS-TB*	Median (IQR): $1.11 (0 – 7.79)	
	*MDR-TB*		Median (IQR): $23.38 (7.79 – 52.33_											*MDR-TB*	Median (IQR): $4.45 (0 – 17.81)	
***Kaswa, 2021*[** **41** **]**							*DS-TB*	Mean (95% CI): $9.20 (7.56 – 10.90)		*DS-TB*		Mean (95% CI): $1.43 (1.09– 1.84)				
							*DR-TB*	Mean (95% CI): $15.67 (9.74 – 21.67)		*DR-TB*		Mean (95% CI): $2.38 (0.95 – 3.88)				
							*Total*	Mean (95% CI): $10.42 (8.38 – 12.40)		*Total*		Mean (95% CI): $1.64 (1.09 – 2.18)				
***Maciel, 2023* [** **46** **]**							*DS-TB*	Mean (95% CI): $40.92 (36.07 – 45.89)		*DS-TB*		Mean (95% CI): $6.24 (5.09 – 7.40)				
							*DR-TB*	Mean (95% CI): $54.10 (39.77 – 68.32)		*DR-TB*		Mean (95% CI): $6.13 (4.86 – 7.40)				
							*Total*	Mean (95% CI): $42.43 (37.92 – 46.93)		*Total*		Mean (95% CI): $6.24 (5.20 – 7.28)				
***Martinez, 2021* [** **50** **]**							*DS-TB*	Mean (95%CI): 6.42 (1.93- 10.91)		*DS-TB*		Mean (95%CI): 3.64 (0.43- 6.74)				
							*DR-TB*	Mean (95%CI): 23.11 (0- 61.63)		*DR-TB*		Mean (95%CI): 16.69 (1.93- 31.56)				
							*Total*	Mean (95%CI): 6.85 (2.25- 11.56)		*Total*		Mean (95%CI): 3.96 (1.07- 6.85)				
***Mauch, 2013*** ^***(2)***^**[52]**	*New*				Median: $53.04											
	*Retreatment*				Median: $128.56											
	*MDR-TB*				Median: $175.79											
***Muttamba, 2020*[** **59** **]**							*DS-TB*	Mean (95% CI): $10.46 (3.42 – 17.50)								
							*MDR-TB*	Mean (95% CI): $5.03 (0.33 – 9.71)								
							*Total*	Mean (95% CI): $10.40 (3.41 – 17.37)								
***Nhung, 2018*[** **61** **]**							*DS-TB*	Mean (95% CI): $46.93 (36.37 – 57.49)		*DS-TB*		Mean (95% CI): $46.93 (30.51 – 64.53)				
							*MDR-TB*	Mean (95% CI): $181.87 (173.65 – 188.91)		*MDR-TB*		Mean (95% CI): $193.60 (52.80 – 334.40)				
							*Total*	Mean (95% CI): $73.92 (68.05 – 78.61)		*Total*		Mean (95% CI): $58.67 (39.89 – 77.44)				
**Panda, 2024[** **62** **]**							*DS-TB*	Median (IQR): $58.54 (5.92 – 125.24)		*DS-TB*		Median (IQR): $15.99 (7.01 – 34.23)		*DS-TB*	Median (IQR): $37.96 (8.37 – 155.50)	
***Pedrazzoli, 2018*[** **63** **]**	*DS-TB*				Median (IQR): $33.32 (31.85 – 37.86)		*DS-TB*	Median (IQR): $28.16 (28.16 – 28.16)		*DS-TB*		Median (IQR): $3.06 (3.06 – 3.06)				
	*MDR-TB*				Median (IQR): $34.59 (33.11 – 39.33)		*MDR-TB*	Median (IQR): $29.21 (29.21 – 29.21)		*MDR-TB*		Median (IQR): $3.06 (3.06 – 3.06)				
	*Total*				Median (IQR): $33.43 (31.85 – 37.86)		*Total*	Median (IQR): $28.16 (28.16 – 28.16)		*Total*		Median (IQR): $3.06 (3.06 – 3.06)				
**Pham, 2023[** **65** **]**							*MDR-TB*	Median (IQR): $63.58 (21.14 – 148.98)		*MDR-TB*		Median (IQR): $17.95 (6.46 – 50.33)				
***Timire, 2021*[** **74** **]**	*DS-TB*				Median (IQR): $31.31 (13.33 - 67.25)		*DS-TB*	Median (IQR): $14.49 (3.77 – 34.79)								
	*DR-TB*				Median (IQR): $20.29 (12.75 – 40.58)		*DR-TB*	Median (IQR): $7.54 (2.32 – 22.61)								
	*Total*				Median (IQR): $ 30.15 (13.33 – 64.35)		*Total*	Median (IQR): $14.49 (3.48 – 33.63)								
***Van der Hof, 2016*[** **79** **]**	Ethiopia	DS-TB				Median (IQR): $25.01 (7.15 – 194.72)								Ethiopia	DS-TB	Median (IQR): $0.00 (0 – 53.59)
		MDR-TB				Median (IQR): $121.48 (62.53 – 341.21)									MDR-TB	Median (IQR): $0.00 (0 – 14.29)
	Indonesia	DS-TB				Median (IQR): $32.15 (8.77 – 62.35)								*Indonesia*	DS-TB	Median (IQR): $3.90 (0 – 8.77)
		MDR-TB				Median (IQR): $37.99 (11.69 – 61.37)									MDR-TB	Median (IQR): $2.92 (0.97 – 5.84)
	Kazakhstan	DS-TB				Median (IQR): $4.06 (0.81 – 10.55)								*Kazakhstan*	DS-TB	Median (IQR): $2.43 (0.81 – 4.06)
		MDR-TB				–									MDR-TB	–
**Youngkong, 2024[** **89** **]**	*DS-TB*			Mean (95%CI): 35.70 (31.84-40.53)			*DS-TB*	Mean (95%CI): 18.33 (15.44- 22.19)		DS-TB		Mean (95%CI): 7.37 (14.47-20.26)		*DS-TB*	Mean (95%CI): 12.54 (10.61- 13.51)	
				Median (IQR): 26.05 (18.33-26.05)				Median (IQR): 13.51 (0.96- 13.51)				Median (IQR): 11.58 (9.65-11.58)			Median (IQR): 6.75(1.93- 12.54)	
	*DR-TB*			Mean (95%CI): 14.47 (8.68-21.23)			*DR-TB*	Mean (95%CI): 0.96 (0.96- 1.93)		DR-TB		Mean (95%CI): 13.51 (7.72-19.30)		*DR-TB*	Mean (95%CI): 27.02 (0 - 55.97)	
				Median (IQR): 13.51 (9.65-13.51)				Median (IQR): 1.93 (0-1.93)				Median (IQR): 11.58 (8.68-11.58)			Median (IQR): 7.72 (0.96-15.44)	
	*Total*			Mean (95%CI): 35.70 (31.84- 40.53)			*Total*	Mean (95%CI): 18.33 (15.44- 21.23)		Total		Mean (95%CI): 17.37 (14.47-20.26)		*Total*	Mean (95%CI): 12.54 (10.61 – 14.47)	
				Median (IQR): 26.05 (16.40- 26.05)				Median (IQR): 13.51 (0.96- 13.51)				Median (IQR): 11.58 (9.65-11.58)			Median (IQR): 6.75 (1.93-12.54)	
***Case Finding Method (ACF/PCF)***																
***Dinh, 2023* [** **28** **]**							*ACF*	Mean (SD): $39.52 (119.57)		*ACF*		Mean (SD): $12.16 (37.49)				
								Median (IQR): $6.08 (0- 20.27)				Median (IQR): $2.03 (1.01- 5.07)				
							*PCF*	Mean (SD): $188.48 (360.75)		*PCF*		Mean (SD): $51.68 (155.04)				
								Median (IQR): $83.09 (40.53- 166.19)							Median (IQR): $10.13 (1.01- 26.35)	
							*Total*	Mean (SD): $114.51 (279.68)		*Total*		Mean (SD): $28.37 (72.96)				
								Median (IQR): $30.40 (3.04- 98.29)							Median (IQR): $4.05 (1.01 - 23.31)	
***Gurung, 2019*[** **40** **]**							*ACF*	Median (IQR): $15.61 (4.91 – 30.23)		*ACF*		Median (IQR): $3.71 (1.96 – 11.35)		*ACF*	Median (IQR): $69.30 (5.46 – 278.41)	
							*PCF*	Median (IQR): $34.49 (12.01 – 86.33)		*PCF*		Median (IQR): $10.59 (2.95 – 41.36)		*PCF*	Median (IQR): $47.26 (15.61 – 270.88)	
							*Total*	Median (IQR): $20.95 (6.88 – 50.53)		*Total*		Median (IQR): $5.89 (2.29 – 24.45)		*Total*	Median (IQR): $55.77 (9.17 – 274.59)	
***Gurung, 2021*[** **39** **]**	*ACF*				Mean (95% CI): $52.28 (32.8–63.0)		*ACF*	Mean (95% CI): $44.86 (31.32–58.50)		*ACF*		Mean (95% CI): $7.42 (3.7–9.9)		*ACF*	Mean (95% CI) - $8.19 (5.6–9.5)	
					Median (IQR): $14.52 (1.4–59.9)			Median (IQR): $13.42 (0–60.90)				Median (IQR): $1.53 (0–5.8)			Median (IQR) - $4.69 (1.9–8.7)	
	*PCF*				Mean (95% CI): $78.03 (56.2–86.8)		*PCF*	Mean (95% CI): $57.95 (45.40–70.50)		*PCF*		Mean (95% CI): $20.08 (11.9–24.8)		*PCF*	Mean (95% CI) - $16.70 (11.9–18.6)	
					Median (IQR): $44.64 (14.0–11.5)			Median (IQR): $32.30 (11.13–86.44)				Median (IQR): $5.78 (1.8–14.1)			Median (IQR) - $10.91 (5.6–18.0)	
	*Total*				Mean (95% CI): $59.90 (49.1–70.7)		*Total*	Mean (95% CI): $51.51 (42.35–60.79)		*Total*		Mean (95% CI): $13.86 (9.0–16.4)		*Total*	Mean (95% CI) - $12.55 (9.4–13.5)	
					Median (IQR): $30.99 (6.2–81.9)			Median (IQR): $23.68 (3.82–76.72)				Median (IQR): $3.27 (0.4–10.8)			Median (IQR) - $7.31 (3.3–13.6)	
***Morishita, 2016*[** **56** **]**	*ACF*				Mean (SD): $15.71 (33.57)									*ACF*	Mean (SD): $23.56 (74.48)	
					Median (IQR): $3.17 (1.52 – 13.93)										Median (IQR): $ 0.00 (0 – 5.19)	
	*PCF*				Mean (SD): $72.71 (173.28)									*PCF*	Mean (SD): $74.86 (412.80)	
					Median (IQR): $18.75 (2.79 – 59.91)										Median (IQR): $1.77 (0 – 5.19)	
***Vo, 2021*[** **82** **]**	*ACF*				Mean (95% CI): $47.10 (23.55 – 70.65)		*ACF*	Mean (95% CI): $39.61 (19.27 – 59.94)		*ACF*		Mean (95% CI): $7.49 (2.14 – 12.85)		*ACF*	Mean (95% CI): $5.35 (0 – 10.70)	
					Median (IQR): $18.20 (6.42 – 36.39)			Median (IQR): $13.92 (4.28 – 33.18)				Median (IQR): $2.14 (0 – 4.28)			Median (IQR): $1.07 (0.54 – 1.07)	
	*PCF*				Mean (95% CI): $165.92 (78.14 – 254.76)		*PCF*	Mean (95% CI): $161.63 (74.93 – 247.27)		*PCF*		Mean (95% CI): $6.42 (3.21 – 9.63)		*PCF*	Mean (95% CI): $21.41 (3.21 -38.54)	
					Median (IQR): $83.49 (34.25 – 164.84)			Median (IQR): $80.28 (32.11 – 179.83)				Median (IQR): $3.21 (1.07 – 8.56)			Median (IQR): $2.14 (1.07 -5.35)	
	*Total*				Mean (95% CI): $102.76 (58.87 – 146.65)		*Total*	Mean (95% CI): $96.34 (53.52 – 139.15)		*Total*		Mean (95% CI): $7.49 (4.28 – 9.63)		*Total*	Mean (95% CI): $12.85 (4.28 – 21.41)	
					Median (IQR): $36.39 (11.77 – 94.20)			Median (IQR): $31.04 (11.77 – 86.70)				Median (IQR): $2.14 (1.07 – 6.42)			Median (IQR): $1.07 (1.07 – 3.21)	
***Public vs Private Health Care Setting***																
***Chandra, 2021*** ^***(1)***^**[20]**	*Public*		Median (IQR): $11.89 (4.74 – 30.11)											*Public*	Median (IQR): $40.53 (17.68 -147.69)	
	*Private*		Median (IQR): $40.53 (20.53 -88.63)											*Private*	Median (IQR): $73.79 (17.47 – 202.53)	
	*Total*		Median (IQR): $33.37 (13.58 -67.16)											*Total*	Median (IQR): $45.90 (17.68 -158.74)	
***McAllister, 2020*[** **54** **]**	*CHC*		Median (IQR): $33.12 (15.53 – 43.96)													
	*Public Hospital*		Median (IQR): $54.07 (29.94 – 117.84)													
	*Private Hospital*		Median (IQR): $38.87 (8.65 – 104.88)													
	*Private Practice*		Median (IQR): $46.60 (27.81 – 71.00)													
***Nguyen, 2023* [** **60** **]**	*Public*		Mean (SD): $251.31 (375.95)				*Public*	Mean (SD): $183.42 (232.06)		*Public*		Mean (SD): $6.08 (3.04- 22.29)				
			Median (IQR): $115.52 (53.71 - 248.27)					Median (IQR): $100.32 (43.57- 204.70)				Median (IQR): $9.12 (4.05- 25.33)				
	*Private*		Mean (SD): $291.84 (461.07)				*Private*	Mean (SD): $271.58 (446.89)		*Private*		Mean (SD): $21.28 (31.41)				
			Median (IQR): $168.22 (93.23- 238.14)					Median (IQR): $155.04 (86.13- 215.84)							Median (IQR): $6.08 (3.04- 22.29)	
	*Total*		Mean (SD): $251.31 (375.95)				*Total*	Mean (SD): $223.95 (347.58)		*Total*		Mean (SD): $28.37 (55.73)				
			Median (IQR): $153.02 (72.96 - 239.15)					Median (IQR): $139.84 (55.73- 214.83)							Median (IQR): $9.12 (4.05- 25.33)	
***Other Stratifications***																
***Chatterjee, 2024* [** **22** **]**	Using HCA 1		Mean: $152.93											Using HCA 1	Mean: $95.83	
	Using HCA 2		Mean: $152.93											Using HCA 2	Mean: $112.17	
	Using OA		–											Using OA	–	
***De Siqueria Filha, 2018*[** **29** **]**							*TB/HIV*	Mean - $78.16		*TB/HIV*		Mean - $55.03		*TB/HIV*	Mean - $459.83	
							*LTBI/HIV*	Mean - $10.86		*LTBI/HIV*		Mean - $19.53		*LTBI/HIV*	Mean - $8.45	
***Lu, 2020*[** **45** **]**							*Residents*	Mean: $1,026.03		*Residents*		Mean: $74.77				
							*Migrants*	Mean: $494.40		*Migrants*		Mean: $34.81				
***Mafirakureva, 2023***^***2***^ **[48]**	*Cameroon*			Median (IQR): $149.73 (1.41 – 356.71)			*Cameroon*	Median (IQR): $74.08 (15.02 – 132.03		*Cameroon*		Median (IQR): $90.72 (13.98 – 142.91)				
	*Kenya*			Median (IQR): $6.52 (0.00 – 23.59)			*Kenya*	Median (IQR): $11.78 (8.63 – 11.97)		*Kenya*		Median (IQR): $3.36 (3.36 – 6.08)				
***Mauch, 2013*** ^***(1)***^ **[** **53** **]**	*Ghana*				Mean: $28.01									*Ghana*	Mean; $344.26	
					Median (IQR): $12.65 (3.61 – 35.25)										Median (IQR): $153.61 (38.85 – 307.22)	
	*Vietnam*				Mean: $130.03									*Vietnam*	Mean: $1,173.05	
												Median (IQR): $11.31 (14.31 – 122.96)			Median (IQR): $1,019.00 (675.57 – 1,454.30)	
	*Dominican Republic*				Mean: $43.35									*Dominican Republic*	Mean: $1,198.92	
												Median (IQR): $9.13 (2.28 – 21.67)			Median (IQR): $759.74 (313.71 – 1,352.92)	
***Pedrazzoli, 2021*[** **64** **]**							*Insured*	Mean: $30.58		*Insured*		Mean: $4.22		*Insured*	Mean: $4.96	
								Median (IQR): $28.47 (28.47 – 28.47)				Median (IQR): $3.06 (3.06 – 3.06)			Median (IQR): $1.69 (0.63 – 3.80)	
							*Uninsured*	Mean: $47.45		*Uninsured*		Mean: $3.16		*Uninsured*	Mean: $ 11.60	
								Median (IQR): $28.47 (28.47 – 29.53)				Median (IQR): $3.06 (3.06 – 3.06)			Median (IQR): $2.32 (0.91 – 5.27)	
***Ramma, 2015*[** **67** **]**	*Inpatients*				Mean: $22.64									*Inpatient*	Mean: $211.53	
	*Outpatient*				Mean: $48.77									*Outpatient*	Mean: $55.04	
***Viney, 2021*[** **81** **]**	*Extra-pulmonary TB*				Median (IQR): $6.59 (5.49 – 10.99)		*Extra-pulmonary TB*	Median (IQR): $0.00 (0 - 0)		*Extra-pulmonary TB*		Median (IQR): $6.59 (5.49 – 10.99)				
	*Pulmonary TB*				Median (IQR): $6.59 (4.39 – 6.59)		*Pulmonary TB*	Median (IQR): $0.00 (0 – 0)		*Pulmonary TB*		Median (IQR): $6.59 (4.39 – 6.59)				
	*Total*				Median (IQR): $6.59 (5.49 – 6.59)		*Total*	Median (IQR): $0.00 (0 – 0)		*Total*		Median (IQR): $6.59 (5.49 – 6.59)				
***Yamanaka, 2024***^***1***^ **[86]**							*TB Only*	Mean (95%CI): $ 27.77 (17.83- 37.1)		*TB Only*		Mean (95%CI): $27.56 (22.60- 32.63)		*TB Only*	Mean (95%CI): $ 208.45 (150.08- 266.92)	
							*TB-DM*	Mean (95%CI): $37.80 (26.14- 49.45)		*TB-DM*		Mean (95%CI): $41.65 (25.94- 57.46)		*TB-DM*	Mean (95%CI): $312.21 (87.35- 537.18)	
							*Total*	Mean (95%CI): $29.08 (20.37- 37.80)		*Total*		Mean (95%CI): $29.49 (24.62- 34.35)		*Total*	Mean (95%CI): $222.23 (163.35- 281.10)	
***Yamanaka, 2024*** ^***2***^ **[87]**							Longitudinal	Mean (95%CI): $29.08 (20.37- 37.80)		Longitudinal	Mean (95%CI): $29.49 (24.62-34.35)			Longitudinal	Mean (95%CI): $222.23 (163.35-281.10)	
							Cross-sectional	20:80	Mean (95%CI): $14.09 (12.57-15.50)	Cross-sectional	20:80		Mean (95%CI): $18.65 (17.33-19.86)	Cross-sectional	20:80	Mean (95%CI): $127.48 (90.80-164.06)
								35:65	Mean (95%CI): $16.31 (14.49-18.14)		35:65		Mean (95%CI): $20.57 (18.04-23.21)		35:65	Mean (95%CI): $182.10 (128.59-235.60)
								50:50	Mean (95%CI): $16.92 (15.10-18.65)		50:50		Mean (95%CI): $23.71 (19.86-27.46)		50:50	Mean (95%CI): $267.22 (183.01-351.53)
***No Stratification***																
***Chandra, 2021*** ^***(2)***^**[19]**	Median (IQR): $33.68 (13.68 – 70.53)						Median (IQR): $25.46 (8.53 – 60.00)			Median (IQR): $3.37 (1.16 – 11.26)				Median (IQR): $0.00 (0.00 – 15.79)		
	Mean (SD): $85.26 (192.63)						Mean (SD): $68.42 (161.05)			Mean (SD): $16.84 (74.21)				Mean (SD): $33.68 (80.00)		
***Ellaban, 2021*[** **31** **]**	Median (IQR): $24.18 (11.89 – 58.86)						Median (IQR): $15.46 (6.24 - 26.06)							Median (IQR): $0.00 (0.00 – 74.31)		
***Fuady, 2020*** ^***(1)***^ **[** **34** **]**	Mean (95% CI): $17.19 (13.96 – 20.41)													Mean (95% CI): $6.44 (4.30 – 7.52)		
***Gadallah, 2022*[** **36** **]**	Mean: $55.49													Mean: $20.81		
	Median: $33.69													Median: $0.00		
***Getahun, 2016*[** **37** **]**	Mean (SD): $41.86 (31.17)													Mean (SD): $24.24 (31.68)		
	Median (Range): $34.05 (228.18)													Median (Range): $19.18 (309.10)		
***Kilale, 2022*[** **42** **]**							Mean (SD): $11.29 (17.44)			Mean (SD): $4.61 (7.46)				Mean (SD): $9.76 (48.91)		
							Median (IQR); $7.90 (7.90 – 7.90)			Median (IQR): $3.29 (3.29 – 3.29)				Median (IQR): $3.40 (1.75 – 6.14)		
***Loureiro, 2024*[** **62** **]**							Mean: $55.24			Mean: $25.43						
***Mauch, 2011*[** **51** **]**	Median (IQR): $3.13 (1.89 – 6.16)													Median: $69.67		
***Muniyandi, 2020*[** **58** **]**	Mean (SD): $66.08 (183.86)													Mean (SD): $122.95 (243.34)		
	Median (IQR): $14.20 (0.00 – 2,435.45)													Median (IQR): $0.00 (0.00 – 1,932.06)		
***Razzaq, 2022*[** **68** **]**							Median (IQR): $21.98 (10.64 – 30.77)			Median (IQR): $4.57 (3.78 – 5.71)				Median (IQR): $25.67 (17.14 – 41.32)		
***Ukwaja, 2013*** ^***(1)***^**[78]**	Median (IQR): $60.42 (48.76 – 105.82)													Mean (SD): $636.43		
	Mean: $86.84															
***Walcott, 2020*[** **83** **]**	Mean: $52.59						Mean: $41.58			Mean: $11.01						
	Median (IQR): $30.58 (13.45 – 72.16)						Median (IQR): $23.24 (8.56 – 50.15)			Median (IQR): $4.89 (1.22 – 12.23)						

**Abbreviations: *TB – Tuberculosis, DS-TB – Drug sensitive TB, DR-TB – Drug-resistant TB, MDR-TB – Multi-drug resistant TB, CHC – Community health centre, SD – Standard deviation, IQR – Interquartile range.***

*Shin - Costs for TB outpatients were extrapolated using the costing data from HIV outpatients.

**Table 3:** Weighted median and means for pre-diagnostic, post-diagnostic and total costs incurred by patients during TB care based on study population.

	Pre-Diagnostic		Post-Diagnostic		Total	
***Median***						
*DS-TB*	N=17	$36.67	N=16	$63.02	N=34	$186.96
*DR-TB/MDR-TB*	N=5	$94.87	N=6	$1,618.58	N=10	$3,798.87
*Mixed Population*	N=0	–	N=0	–	N=5	$200.00
*HIV* ^+^	N=0	–	N=0	–	N=2	$114.38
***Mean***						
*DS-TB*	N=12	$392.88	N=14	$680.61	N=33	$1,259.48
*DR-TB/MDR-TB*	N=0	–	N=0	–	N=12	$8,946.64
*Mixed Population*	N=0	–	N=0	–	N=7	$455.00
*HIV* ^+^	N=2	$87.62	N=2	$592.37	N=5	$120.04

Abbreviations: *TB – Tuberculosis, DS-TB – drug sensitive TB, DR-TB – drug resistant TB, MDR-TB – multi-drug resistant TB, HIV – human immunodeficiency virus.*

*A number of studies included costs and population sizes for different subgroups within the patient population.

Non-medical costs ranged from a mean of $3.60 to $725 and a median of $0.88 to $63.63 (S3 Table). Major contributors to non-medical costs were nutritional supplements, food, and transportation. Patients with DR-TB and those identified via PCF methods incurred higher non-medical costs except for food costs where DS-TB patients had slightly higher costs than DR-TB patients.

Indirect costs made up the largest portion of pre-diagnostic costs incurred by patients ranging from a mean of $1.26 to $271 and a median of $0.34 to $2,837 (S4 Table), with loss of income being largest single cost contributor (mean: $0.61-$611, median: $0.43-$2,837).

### Post-diagnostic patient costs

Total post-diagnostic costs incurred by patients were reported in 21 studies. For DS-TB patients median post diagnostic costs ranged from $33.12 to $1,889. For DR-TB patients, post-diagnostic costs ranged from a median of $975 to $5,194, with a weighted median of $1,619. Among DS-TB, a weighted median of $63.01 and weighted mean of $680 was estimated (Table 3).

Direct medical costs were the largest component costs, they ranged from a mean of $0.03 to $17,140 while median costs ranged from $5.16 to $14,093 (Table 4). Cost factors that had the biggest impact on medical costs included medication, follow-up tests and hospitalization. Medical costs were highest amongst DR-TB patients (S5 Table).

**Table 4:** Total direct and indirect costs incurred by patients during the post-diagnostic phase of TB care.

	*Direct Costs*															*Indirect Costs*					
	*Total*				*Medical*					*Non-Medical*											
***TB Type (DS-TB, DR-TB, MDR-TB)***																					
***Aia, 2022*[** **14** **]**	*DS-TB*	Mean (95% CI): $183.20 (149.48 – 218.04)			*DS-TB*		Mean (95% CI): $29.33 (13.26 – 45.41)			*DS-TB*				Mean (95% CI): $153.97 (125.99 – 182.86)		*DS-TB*	Mean (95% CI): $421.46 (356.16 – 487.55)				
	*MDR-TB*	Mean (95% CI): $1,603.80 (386.85 - 2,830.99)			*MDR-TB*		Mean (95% CI): $3.37 (3.09 – 9.78)			*MDR-TB*				Mean (95% CI): $1,600.43 (371.45 – 2,828.29)		*MDR-TB*	Mean (95% CI): $1,801.61 (296.60 – 3,899.82)				
	*Total*	Mean (95% CI): $210.17 (169.15 – 274.68)			*Total*		Mean (95% CI): $28.77 (13.15 – 44.51)			*Total*				Mean (95% CI): $182.07 (144.65 – 218.94)		*Total*	Mean (95% CI): $448.44 (373.25 – 522.84)				
***Chittamany, 2020*[** **23** **]**					*DS-TB*		Median (IQR): $25.65 (12.35 – 28.50)			*DS-TB*				Median (IQR): $310.69 (160.57 – 666.99)		*DS-TB*	Median (IQR): $30.40 (0.00 – 536.82)				
					*DR-TB*		Median (IQR): $199.53 (137.77 – 455.11)			*DR-TB*				Median (IQR): $252.73 (198.58 -751.55)		*DR-TB*	Median (IQR): $1,257.97 (302.14 – 1,787.19)				
					*Total*		Median (IQR): $27.55 (12.35 – 28.50)			*Total*				Median (IQR): $310.69 (164.37 – 667.94)		*Total*	Median (IQR): $54.16 (0.00 –536.82)				
Collins, 2018*[25]	*MDR-TB*	*Intensive phase*		Median (Range): $1,141.53 (462.69 – 1,729.26)												*MDR-TB*	*Intensive phase*		Median (Range): $0.00 (0.00 – 0.00)		
		*Continuation Phase*		Median (Range): $1,132.60 (818.19 – 1,872.18)													*Continuation Phase*		Median (Range); $0.00 (0.00 – 0.00)		
***Deribew, 2024* [** **26** **]**	Baseline	Mean: $454.04			Baseline		Mean: $143.86			Baseline				Mean: $259.91		Baseline	Mean: $50.27				
	Follow-up	Mean: $168.15			Follow-up		Mean: $51.01			Follow-up				Mean: $61.50		Follow-up	Mean: $55.76				
***Diallo, 2022*[** **27** **]**					DS-TB		Mean (95% CI): $95.64 (41.98 – 148.62)									DS-TB	Mean (95% CI): $853.84 (675.38 – 1,032.30)				
					DR-TB		Mean (95% CI): $117.20 (59.00 – 293.84)									DR-TB	Mean (95% CI): $521.54 (16.73 – 1,026.40)				
					Total		Mean (95% CI): $95.98 (44.25 – 147.49)									Total	Mean (95% CI): $848.17 (672.66 – 1,023.56)				
***Fuady, 2018*[** **35** **]**					*DS-TB*		Median (IQR): $0.00 (0.00 – 6.68)			*DS-TB*				Median (IQR): $36.74 (8.91 – 107.99)		*DS-TB*	Median (IQR): $8.91 (0.00 – 498.77)				
					*MDR-TB*		Median (IQR): $16.70 (0.00 – 86.84)			*MDR-TB*				Median (IQR): $953.00 (478.73 – 1,588.70)		*MDR-TB*	Median (IQR): $1,496.30 (2.23 – 2,869.02)				
***Kaswa, 2022*[** **41** **]**					DS-TB		Mean (95% CI): $35.43 (15.74 – 54.58)			DS-TB				Mean (95% CI): $116.51 (88.10 – 145.40)							
					DR-TB		Mean (95% CI): $62.68 (20.24 – 104.72)			DR-TB				Mean (95% CI): $387.00 (233.48 – 550.79)							
					Total		Mean (95% CI): 40.20 (21.12 – 59.07)			Total				Mean (95% CI): $165.56 (127.34 – 203.92)							
***Maciel, 2023 [*** **46** ***]***					DS-TB		Mean (95% CI): $100.00 (58.50 – 141.50)														
					DR-TB		Mean (95% CI): $88.09 (31.68 – 144.50)														
					Total		Mean (95% CI): $98.72 (62.77 – 134.68)														
***Martinez, 2021* [** **50** **]**					DS-TB		Mean (95% CI): 9.74 (4.60- 14.87)			DS-TB				Mean (95% CI): 876.38 (789.82- 962.93)							
					DR-TB		Mean (95% CI): 108.70 (0-279.36)			DR-TB				Mean (95% CI): 2337.79 (1128.77- 3545.74)							
					Total		Mean (95% CI): 12.52 (6.42- 18.62)			Total				Mean (95% CI): 917.14 (826.95- 1007.34)							
***Mauch, 2013*** ^***(2)***^ **[** **52** **]**	*New*	Median: $144.42														*New*	Median: $828.64				
	*Retreatment*	Median: $105.63														*Retreatment*	Median: $251.99				
	*MDR*	Median: $230.89														*MDR-TB*	Median: $3,588.67				
***Medeiros, 2024* [** **55** **]**	*MDR-TB*	*Patient’s*		Median: $586.01												*MDR-TB*	Median: $4,573.68				
		*Caretaker’s*		Median: $29.65																	
		*Total*		Median: $648.77																	
***Muttamba, 2020*[** **59** **]**																*MDR-TB*	Mean (95% CI): $1,490.93 (658.01 – 2,325.06)				
																*DS-TB*	Mean (95% CI): $142.49 (118.64 – 166.34)				
																*Total*	Mean (95% CI): $192.02 (143.10 – 240.95)				
***Nhung, 2018*[** **61** **]**					*MDR-TB*		Mean (95% CI): $746.24 (468.16 – 1,025.50)			*MDR-TB*				Mean (95% CI): $2,310.30 (1,670.83 – 2,949.77)		*MDR-TB*	Mean (95% CI); $3,726.52 (726.30 – 2.503.90)				
					*DS-TB*		Mean (95% CI): $110.29 (75.09 – 145.49)			*DS-TB*				Mean (95% CI): $435.31 (319.15 – 552.64)		*DS-TB*	Mean (95% CI): $596.06 (504.53 – 719.26)				
					*Total*		Mean (95% CI): $160.75 (110.29 – 211.20)			*Total*				Mean (95% CI): $586.67 (450.56 – 721.60)		*Total*	Mean (95% CI): $677.02 (554.99 – 801.39)				
***Panda, 2024* [** **62** **]**					*DS-TB*		Median (IQR): $45.81 (10.24 – 93.24)			*DS-TB*				Median (IQR): $14.29 (2.91 – 31.99)		*DS-TB*	Median (IQR): $156.27 (22.74 – 375.23)				
***Pedrazzoli, 2018*[** **63** **]**					*MDR-TB*		Median (IQR): $42.92 (8.75 - 106.82)			*MDR-TB*				Median (IQR): $450.92 (118.21 – 1,119.18)		*MDR-TB*	Median (IQR): $0.00 (0.00 – 228.73)				
					*DS-TB*		Median (IQR); $78.04 (58.84 – 81.20)			*DS-TB*				Median (IQR): $148.27 (32.69 – 450.92)		*DS-TB*	Median (IQR): $0.00 (0.00 –205.85)				
					*Total*		Median (IQR): $73.82 (58.84 – 81.73)			*Total*				Median (IQR): $157.44 (34.06 – 552.58)		*Total*	Median (IQR): $92.06 (0.00 – 533.60)				
***Pham, 2023*[** **65** **]**					*MDR-TB*		Median (IQR): $41.10 (7.89 – 118.70)			*MDR-TB*				Median (IQR): $571.00 (332.18 – 974.22)		*MDR-TB*	Median (IQR): $2,225.51 (793.54 – 4,382.50)				
***Timire, 2021*[** **74** **]**	*DS-TB*	Median (IQR): $311.33 (118.27 – 657.44)			*DS-TB*		Median (IQR: $52.76 (35.83 – 77.69)									*DS-TB*	Median (IQR): $139.14 (0.00 – 626.13)				
	*DR-TB*	Median (IQR): $961.81 (378.58 – 1,615.78)			*DR-TB*		Median (IQR): $120.01 (74.79 – 171.03									*DR-TB*	Median (IQR): $695.71 (57.98 – 1,739.26)				
	*Total*	Median (IQR): $321.76 (127.55 – 693.97)			*Total*		Median (IQR): $52.87 (36.76 – 87.66)									*Total*	Median (IQR): $173.93 (0.00 – 695.71)				
***Tomeny, 2020*** ^*^ **[** **75** **]**					*DS-TB*		Mean: $74.69									*DS-TB*	Mean: $74.69				
					*MDR-TB*		Mean: $66.76									*MDR-TB*	Mean: $1,566.15				
***Van der Hof, 2016*[** **79** **]**		DS-TB		MDR-TB													DS-TB				MDR-TB
	Ethiopia	Intensive Phase	Median (IQR): $185.79 (17.86 – 412.67)	Median (IQR): $1,141.53 (462.69 – 1,729.26)												Ethiopia	Intensive Phase	DS-TB			MDR-TB
		Continuation Phase	Median (IQR): $142.91 (60.74 – 278.68)	Median (IQR): $1,132.60 (818.19 – 1,872.18)													Continuation Phase	Median (IQR): $0.00 (0.00 – 60.74)			Median (IQR): $398.01 (158.99 – 668.12)
	Indonesia	Intensive Phase	Median (IQR): $39.94 (7.79 – 105.21)	Median (IQR): $580.60 (333.16 – 1,008.25)												Indonesia	Intensive Phase	Median (IQR): $0.00 (0.00 – 7.15)			Median (IQR): $130.41 (1.79 – 669.91)
		Continuation Phase	Median (IQR): $57.48 (16.56 – 218.21)	Median (IQR): $950.78 (543.58 – 1,543.06)													Continuation Phase	Median (IQR): $9.74 (0.00 – 38.97)			Median (IQR): $306.86 (149.05 – 826.08)
	Kazakhstan	Intensive Phase	Median (IQR): $0.00 (0.00 – 60.03)	Median (IQR): $133.85 (0.00 – 438.87												Kazakhstan	Intensive Phase	Median (IQR): $8.77 (0.00 – 55.53)			Median (IQR): $247.44 (0.00 – 490.98)
		Continuation Phase	Median (IQR): $145.21 (73.01 – 266.08)	Median (IQR): $611.66 (279.06 – 1,640.29)													Continuation Phase	Median (IQR): $327.73 (245.80 – 546.76)			Median (IQR): $1,246.85 (0.00 – 2,187.06)
***Yang, 2020*[** **88** **]**					*Hospitalized*		*RS-TB*	Mean (SD): $3,399.81 (3,398.45)		*Hospitalized*		*RS-TB*			Mean (SD): $2,196.87 (2,751.77)						
								Median (IQR): $2,555.86 (1,751.45 – 3,573.59)							Median (IQR): $1,491.57 (866.18 – 2,416.06)						
							*RMR-TB*	Mean (SD): $12,311.87 (9.422.82)				*RMR-TB*			Mean (SD): $8,872.63 (9,653.13)						
								Median (IQR): $11,676.32 (5,239.99 – 16,962.98)													Median (IQR): $4,521.43 (2,649.10 – 12,701.81)
							*MDR-TB*	Mean (SD): $13,172.18 (8,655.43)				*MDR-TB*			Mean (SD): $10,137.76 (9,115.42)						
								Median (IQR): $11,901.59 (7,450.18 – 17,697.12)													Median (IQR): $8,335.76 (3,624.14 – 13,793.37)
					*Non-Hospitalized*		*RS-TB*	Mean (SD): $ 1,116.94 (1,180.72)		*Non-Hospitalized*		*RS-TB*			Mean (SD): $737.71 (1,043.43)						
								Median (IQR): $900.90 (590.46 – 1,316.84)													Median (IQR): $543.79 (310.75 – 1,006.09)
							*RMR-TB*	Mean (SD): $2,378.72 (3093.78)				*RMR-TB*			Mean (SD): $1,031.68 (1,018.67)						
								Median (IQR): $1,615.85 (512.75 – 2,559.74)													Median (IQR): $800.21 (97.12 - 1,914.96)
							*MDR-TB*	Mean (SD): $1,429.48 (1084.01)				*MDR-TB*			Mean (SD): $897.33 (1,083.70)						
								Median (IQR): $1,771.27 (536.03 – 1,981.03)													Median (IQR): $365.08 (0.00 – 1,608.09)
***Chandra, 2021*** ^***(2)***^ **[** **19** **]**	Median (IQR): $13.68 (1.20 – 71.58)				Median (IQR): $0 (0.00 – 0.00)					Median (IQR): $13.68 (1.20 – 66.32)						Median (IQR): $0.00 (0.00 – 110.53)					
	Mean (SD): $45.26 (60.00)				Mean (SD): $1.79 (11.16)					Mean (SD): $43.16 (58.95)						Mean (SD): $120.00 (242.11)					
***Treatment Stages (Intensive, Continuation)***																					
***Chatterjee, 2024* [** **22** **]**	*Intensive Phase*	using HCA 1		Mean: $63.33												*Intensive Phase*	using HCA 1			Mean: $18.15	
		using HCA 2		Mean: $63.33													using HCA 2			Mean: $19.26	
		using OA		Mean: $63.33													using OA			Mean: $ 220.48	
	*Continuation Phase*	using HCA 1		Mean: $74.17												*Continuation Phase*	using HCA 1			Mean: $15.66	
		using HCA 2		Mean: $74.17													using HCA 2			Mean: $17.23	
		using OA		Mean: $74.17													using OA			Mean: $271.94	
	*Entire Illness*	using HCA 1		Mean: $290.43												*Entire Illness*	using HCA 1			Mean: $129.64	
		using HCA 2		Mean: $290.43													using HCA 2			Mean: $148.65	
		using OA		Mean: $290.43													using OA			Mean: $492.42	
***Ellaban, 2021*[** **31** **]**	*First two months of treatment (intensive phase)*	Median (IQR): $7.73 (3.77 – 14.86)			*First two months of treatment (intensive phase)*		Median (IQR): $0.00 (0.00 – 0.00)									*First two months of treatment (intensive phase)*	Median (IQR_: $0.00 (0.00 – 92.94)				
	*Second two months of treatment*	Median (IQR): $3.77 (2.48 – 9.31)			*Second two months of treatment*		Median (IQR): $0.00 (0.00 – 0.00)									*Second two months of treatment*	Median (IQR): $0.00 (0.00 – 74.31)				
	*Third two months of treatment*	Median (IQR): $2.48 (1.29 – 5.55)			*Third two months of treatment*		Median (IQR); $0.00 (0.00 – 0.00)									*Third two months of treatment*	Median (IQR): $0.00 (0.00 – 61.93)				
***Gadallah, 2022*[** **36** **]**	*Intensive Phase*	Mean: $13.87														*Intensive Phase*	Mean: $26.75				
		Median: $2.97															Median: $12.88				
	*Continuation Phase*	Mean: $18.83														*Continuation Phase*	Mean: $60.44				
												Median: $12.88					Median: $35.67				
***Ramma, 2015*[** **67** **]**	*Intensive Phase*	Mean: $25.43														*Intensive Phase*	Mean: $168.51				
	*Continuation Phase*	Mean: $50.94														*Continuation Phase*	Mean: $110.60				
***Razzaq, 2022*[** **68** **]**					*Diagnostics*		Median (IQR): $7.03 (0.26 – 16.00)			*Diagnostics*				Median (IQR): $3.78 (2.64 – 10.64)		*Diagnostics*	Median (IQR): $5.71 (3.52 – 8.35)				
					*Intensive Phase*		Median (IQR): $0.00 (0.00 – 0.00)			*Intensive Phase*				Median (IQR): $2.73 (1.49 – 4.84)		*Intensive Phase*	Median (IQR): $5.27 (3.08 – 8.88)				
					*Continuation Phase*		Median (IQR); $2.37 (1.14 – 7.91)			*Continuation Phase*				Median (IQR): $3.08 (1.14 – 6.86)		*Continuation Phase*	Median (IQR): $4.31 (2.29 – 7.65)				
***Case Finding Method (ACF, PCF)***																					
***Dinh, 2023* [** **28** **]**	*ACF*	Mean (SD): 369.87 (535.05)			*ACF*		Mean (SD): 80.05 (237.12)			*ACF*				Mean (SD): 289.82 (336.43)							
		Median (IQR): 226.99 (82.08- 414.46)					Median (IQR): 7.09 (0- 34.45)							Median (IQR): 190.51 (66.88- 360.75)							
	*PCF*	Mean (SD): 430.67 (563.42)			*PCF*		Mean (SD): 97.28 (190.51)			*PCF*				Mean (SD): 338.46 (508.70)							
		Median (IQR): 279.68 (116.53- 598.89)					Median (IQR): 35.47 (0- 106.40)													Median (IQR): 234.08 (96.27- 424.59)	
	*Total*	Mean (SD): 400.27 (549.23)			*Total*		Mean (SD): 88.16 (214.83)			*Total*				Mean (SD): 314.14 (431.69)							
		Median (IQR): 248.27 (103.36- 547.21)					Median (IQR): 13.17 (0- 79.04)													Median (IQR): 220.91 (86.13- 401.29)	
***Gurung, 2019*[** **40** **]**					*ACF*		–			*ACF*				Median (IQR): $0.00 (0.00 – 15.72)		*ACF*	Median (IQR): $59.92 (32.63 – 104.66)				
					*PCF*		Median (IQR): $0.00 (0.00 – 4.37)			*PCF*				Median (IQR): $1.42 (0.00 – 48.89)		*PCF*	Median (IQR): $65.05 (38.09 – 90.26)				
					*Total*		–			*Total*				Median (IQR): $0.00 (0.00 – 30.56)		*Total*	Median (IQR): $60.13 (32.63 – 98.77)				
***Gurung, 2021*[** **39** **]**	*ACF*	Mean (95% CI): $29.69 (22.05 – 37.22)			*ACF*		Mean (95% CI): $21.28 (14.73 – 27.83)			*ACF*				Mean (95% CI): $8.29 (5.57 – 11.13)		*ACF*	Mean (95% CI): $42.24 (36.23 – 48.13)				
		Median (IQR): $17.68 (10.37 – 33.94)					Median (IQR): $11.79 (6.88 – 22.59)							Median (IQR) $2.07 (0.33 – 10.26)			Median (IQR): $32.30 (23.14 – 54.68)				
	*PCF*	Mean (95% CI): $33.40 (25.43 – 41.36)			*PCF*		Mean (95% CI): $23.14 (16.26 – 29.90)			*PCF*				Mean (95% CI): $10.37 (7.42 – 13.21)		*PCF*	Mean (95% CI): $48.13 (36.45 – 59.81)				
		Median (IQR): $19.54 (13.10 – 41.14)					Median (IQR): $13.31 (7.86 – 23.14)							Median (IQR): $3.71 (0.76 – 14.41)			Median (IQR): $30.45 (18.55 – 56.21)				
	*Total*	Mean (95% CI): $31.54 (26.08 – 37.00)			*Total*		Mean 95% CI): $22.15 (17.46 – 26.85)			*Total*				Mean (95% CI): $9.39 (7.31 – 11.35)		*Total*	Mean (95% CI): $45.18 (38.63 – 51.62)				
		Median (IQR): $19.54 (11.68 – 35.25)					Median (IQR): $12.88 (7.20 – 22.59)							Median (IQR): $2.95 (0.76 – 11.79)			Median (IQR): $31.76 (21.28 – 54.79)				
***Vo, 2021*[** **82** **]**	ACF	Mean (95% CI): $104.90 (79.21 – 130.59)			ACF		Mean (95% CI): $57.80 (41.75 – 74.93)			ACF				Mean (95% CI): $44 (31-57)		ACF	Mean (95% CI): $13.92 (7.49 – 21.41)				
		Median (IQR): $70.65 (46.03 – 129.52)					Median (IQR): $36.39 (23.55 – 65.30)							Median (IQR): $27 (12-59)			Median (IQR): $6.42 (4.28 – 13.92)				
	PCF	Mean (95% CI): $457.07 (104.90 – 809.24)			PCF		Mean (95% CI): $411.04 (81.35 – 741.80)			PCF				Mean (95% CI): $46.03 (14.99 -76.00)		PCF	Mean (95% CI): $28.90 (1.07 – 56.73)				
		Median (IQR): $96.34 (53.52 – 208.73)					Median (IQR): $81.35 (39.61 – 164.84)							Median (IQR): $14.99 (8.56 -40.68)			Median (IQR): $5.35 (4.28 – 8.56)				
	Total	Mean (95% CI): $270.82 (103.83 – 436.73)			Total		Mean (95% CI): $223.72 (67.44 – 380.00)			Total				Mean (95% CI): $46.03 (31.04 – 62.08)		Total	Mean (95% CI): $21.41 (7.49 – 35.32)				
		Median (IQR): $88.84 (49.24 – 171.27)					Median (IQR): $50.31 (25.69 – 99.55)							Median (IQR): $21.41 (10.70 – 50.31)			Median (IQR): $5.35 (4.28 -11.77)				
***HIV Status (TB, TB/HIV)***																					
***De Siqueria Filha, 2018*[** **29** **]**					*TB/HIV*		Mean: $95.17			*TB/HIV*				Mean: $132.29		*TB/HIV*	Mean: $617.37				
					*LTBI/HIV*		Mean: $63.98			*LTBI/HIV*				Mean: $27.87		*LTBI/HIV*	Mean: $36.11				
***Mudzengi, 2017*[** **57** **]**	*TB/HIV*	Mean: $18.91			*Study Clinic*		*TB/HIV*	Mean (SD): $0.00 (0.00)								*TB/HIV*	Mean: $45.58				
							*TB*	Mean (SD): $0.00 (0.00)													
	*TB*	Mean: $8.59					*HIV*	Mean (SD): $0.00 (0.00)								*TB*	Mean: $50.92				
							*Other Facilities*							*TB/HIV*	Mean (SD): $1.49 (8.91)						
			*HIV*	Mean: $15.92			*TB*	Mean (SD): $0.06 (0.37)								*HIV*	Mean: $19.27				
														*HIV*	Mean (SD): $0.86 (3.59)						
***Rupani, 2022*[** **70** **]**					*TB*		Median (IQR): $0.00 (0 – 15.89)			*TB*				Median (IQR): $30.71 (21.18 – 56.13)		*TB*	Median (IQR): $19.06 (10.59 – 33.89)				
					*TB/HIV*		Median (IQR): $0.00 (0 – 26.48)			*TB/HIV*				Median (IQR): $49.78 (31.77 – 80.49)		*TB/HIV*	Median (IQR): $34.95 (19.06 – 57.19)				
***Public vs Private Health Care Setting***																					
***McAllister, 2020*[** **54** **]**	*CHC*	Median (IQR): $12.12 (8.18 – 17.46)														*CHC*	Median (IQR): $162.93 (113.44 – 738.85)				
	*Public Hospital*	Median (IQR): $43.38 (14.32 – 72.30)														*Public Hospital*	Median (IQR): $268.26 (64.42 – 757.79)				
	*Private Hospital*	Median (IQR): $32.38 (20.46 – 71.65)														*Private Hospital*	Median (IQR): $199.30 (56.84 – 530.46)				
	*Private Practice*	Median (IQR): $84.57 (32.96 – 151.93)														*Private Practice*	Median (IQR): $106.09 (73.26 – 795.68)				
***Nguyen, 2023* [** **60** **]**	*Public*	Mean (SD): $1504.82 (1310.26)			*Public*		Mean (SD): $384.06 (483.37)			*Public*				Mean (SD): $358.72 (254.35)		*Public*	Mean (SD): $1038.68 (1286.95)				
		Median (IQR): $1125.83 (534.03 - 2021.63)					Median (IQR): $200.64 (15.20 - 629.29)							Median (IQR): $319.20 (190.51 - 434.73)			Median (IQR): $585.71 (0- 1547.38)				
	*Private*	Mean (SD): $2996.47 (4448.59)			*Private*		Mean (SD): $716.44 (482.35)			*Private*				Mean (SD): $525.93 (667.80)		*Private*	Mean (SD): $1755.12 (4176.00)				
		Median (IQR): $1888.88 (981.93- 3006.60)					Median (IQR): $617.13 (354.67 - 982.95)							Median (IQR): $389.13 (132.75 - 645.50)			Median (IQR): $719.48 (0- 1830.10)				
	*Total*	Mean (SD): $2182.75 (3222.44)			*Total*		Mean (SD): $384.06 (483.37)			*Total*				Mean (SD): $434.73 (492.49)		*Total*	Mean (SD): $1364.98 (2977.21)				
		Median (IQR): $1561.57 (746.84- 2510.06)					Median (IQR): $200.64 (15.20 - 629.29)							Median (IQR): $333.39 (155.04- 537.07)			Median (IQR): $592.81 (0- 1568.66)				
***Other Stratifications***																					
***Bengey, 2023* [** **17** **]**					Intensive Phase	Cross Sectional Approach #1	Mean (95% CI): $25.83 (14.27 – 37.39)			Intensive Phase				Cross Sectional Approach #1	Mean (95% CI): $6.84 (5.08 – 8.60)	Intensive Phase		Cross Sectional Approach #1		Mean (95% CI): $57.52 (48.65 – 66.41)	
							Median (IQR): $7.98 (3.42 – 19.87)								Median (IQR): $2.36 (0.00 – 6.51)					Median (IQR): $34.67 (11.87 – 85.95)	
						Cross Sectional Approach #2	Mean (95% CI): $4.28 (3.66 – 4.89)							Cross Sectional Approach #2	Mean (95% CI): $4.67 (3.35 – 5.98)			Cross Sectional Approach #2		Mean (95% CI): $47.88 (41.35 – 54.43)	
										Median (IQR): $2.82 (1.55 – 4.83)							Median (IQR): $0.00 (0.00 – 4.77)			Median (IQR): $19.11 (6.99 – 93.04)	
						Longitudinal Approach	Mean (95% CI): $13.13 (8.92 – 17.33)							Longitudinal Approach	Mean (95% CI): $5.51 (4.28 – 6.75)			Longitudinal Approach		Mean (95% CI): $49.80 (43.37 – 56.23)	
										Median (IQR): $5.59 (2.90 – 11.62)							Median (IQR): $2.00 (0.26 – 5.72)			Median (IQR): $32.42 (11.70 – 78.90)	
					Continuation Phase	Cross Sectional Approach #1	–			Continuation Phase				Cross Sectional Approach #1	–	Continuation Phase		Cross Sectional Approach #1		–	
							–								–					–	
						Cross Sectional Approach #2	Mean (95%): $8.54 (7.31 – 9.76)							Cross Sectional Approach #2	Mean (95% CI): $9.19 (6.66 -11.72)			Cross Sectional Approach #2		Mean (95% CI): $95.39 (82.37 – 108.40)	
							Median (IQR): $5.64 (3.09 – 9.66)								Median (IQR): $0.00 (0.00 – 9.53)					Median (IQR): $38.24 (13.84 – 186.08)	
						Longitudinal Approach	Mean (95% CI): $8.39 (6.99 – 9.78)							Longitudinal Approach	Mean (95% CI): $3.56 (2.60 – 4.50)			Longitudinal Approach		Mean (95% CI): $97.04 (84.34 – 109.74)	
							Median (IQR): $5.67 (3.37 – 9.77)								Median (IQR): $0.00 (0.00 – 3.63)					Median (IQR): $39.04 (17.73 – 189.78)	
***Lu, 2020*[** **45** **]**					*Residents*		Mean: $1,785.67			*Residents*				Mean: $162.44							
					*Migrants*		Mean: $1,042.46			*Migrants*				Mean: $239.62							
***Mafirakureva, 2023*** ^***1***^**[47]**		*Cameroon*		*Uganda*			*Cameroon*		*Uganda*				*Cameroon*		*Uganda*			*Cameroon*		*Uganda*	
	*SOC*	Median (IQR): $59.95 (10.20 – 242.61)		Median (IQR): $30.19 (18.92 – 91.56)	*SOC*		Median (IQR): $0.00 (0.00 – 27.91)		Median (IQR): $6.70 (0.00 – 23.86)	*SOC*			Median (IQR): $13.85 (5.19 – 45.51)		Median (IQR): $23.26 (13.07 – 64.01)	*SOC*		Median (IQR): $7.73 (5.83 – 15.64)		Median (IQR): $8.00 (4.56 – 14.49)	
	*Intervention*	Median (IQR): $0.00 (0.00 -11.89)		Median (IQR): $0.00 (0.00 – 0.00)	*Intervention*		Median (IQR): $15.58 (0.00 – 92.07)		Median (IQR): $0.00 (0.00 – 0.00)	*Intervention*			Median (IQR): $0.00 (0.00 – 0.00)		Median (IQR): $0.00 (0.00 – 0.00)	*Intervention*		Median (IQR): $2.84 (2.84 – 5.67)		Median (IQR): $3.30 (1.65 – 4.95)	
	*Total*	Median (IQR): $0.00 (0.00 – 54.08)		Median (IQR): $0.00 (0.00 – 31.12)	*Total*		Median (IQR): $0.00 (0.00 – 27.91)		Median (IQR): $0.00 (0.00 – 6.10)	*Total*			Median (IQR): $0.00 (0.00 – 5.66)		Median (IQR): $0.00 (0.00 – 23.56)	*Total*		Median (IQR): $4.34 (2.84 – 8.50)		Median (IQR): $4.95 (3.30 – 8.00)	
***Mafirakureva, 2023*** ^***2***^**[48]**	*Cameroon*	Median (IQR): $58.31 (8.96 – 221.21)			*Cameroon*		Median (IQR): $0.00 (0.00 – 44.96)														
	*Kenya*	Median (IQR): $80.36 (32.80 – 162.83)			*Kenya*		Median (IQR): $0.00 (0.00 – 29.30)														
***Mauch, 2013*** ^***(1)***^ **[** **53** **]**	*Ghana*	Mean: $103.01														*Ghana*	Mean: $10.84				
		Median (IQR): $16.26 (4.52 – 46.99)															Median (IQR): $0.00 (0.00 – 0.00)				
	*Vietnam*	Mean: $103.17														*Vietnam*	Mean: $36.75				
												Median (IQR): $31.09 (14.13 – 90.45)					Median (IQR): $9.89 (4.24 – 16.96)				
	*Dominican Republic*	Mean: $125.48														*Dominican Republic*	Mean: $78.71				
												Median (IQR): $13.69 (5.70 – 30.80)					Median (IQR): $63.88 (22.81 – 90.12)				
***Mauch, 2011*[** **51** **]**	*Cost per visit*	Median: $0.38																			
	*Overall treatment cost*	Median (IQR): $5.35 (2.13 – 9.71)																			
***McAllister, 2020*[** **54** **]**	*CHC*	Median (IQR): $12.12 (8.18 – 17.46)														*CHC*	Median (IQR): $162.93 (113.44 – 738.85)				
	*Public Hospital*	Median (IQR): $43.38 (14.32 – 72.30)														*Public Hospital*	Median (IQR): $268.26 (64.42 – 757.79)				
	*Private Hospital*	Median (IQR): $32.38 (20.46 – 71.65)														*Private Hospital*	Median (IQR): $199.30 (56.84 – 530.46)				
	*Private Practice*	Median (IQR): $84.57 (32.96 – 151.93)														*Private Practice*	Median (IQR): $106.09 (73.26 – 795.68)				
***Pedrazzoli, 2021*[** **64** **]**					*Uninsured*		Mean: $140.25			*Uninsured*				Mean: $478.76		*Uninsured*	Mean: $325.85				
							Median (IQR): $87.53 (87.53 – 106.51)							Median (IQR): $159.24 (32.69 – 546.26)			Median (IQR): $0.00 (0.00 – 266.80)				
					*Insured*		Mean: $149.74			*Insured*				Mean: $461.89		*Insured*	Mean: $338.51				
							Median (IQR): $87.53 (87.53 – 136.04)							Median (IQR): $160.29 (39.02 – 562.07)			Median (IQR): $0.00 (0.00 – 213.02)				
***Prasanna, 2018*[** **66** **]**	*Study population*	Median (IQR): $69.68 (23.80 – 167.00)														*Study Population*	Median (IQR): $53.57 (0.96 – 314.89)				
	*Those who incurring costs*	Median (IQR): $72.99 (25.93 – 167.32)														*Those who incurred costs*	Median (IQR): $142.24 (9.28 – 509.85)				
***Ramma, 2015*[** **67** **]**	*Inpatients*	Mean: $22.64														*Inpatients*	Mean: $211.53				
	*Outpatients*	Mean: $48.77														*Outpatients*	Mean: $55.04				
***Rupani, 2020*[** **69** **]**					*Private Provider*		Median (IQR): $31.58 (10.53 – 80.00)			*Private Provider*				Median (IQR): $4.21 (2.11 – 5.26)		*Private Provider*	Median (IQR): $21.05 (3.16 – 80.00)				
					*Public Provider*		Median (IQR): $0.00 (0.00 – 0.00)			*Public Provider*				Median (IQR): $3.16 (2.11 – 4.21)		*Public Provider*	Median (IQR): $4.21 (3.16 – 10.53)				
					*Total*		Median (IQR): $0.00 (0.00 – 0.00)			*Total*				Median (IQR): $3.16 (2.11 – 4.21)		*Total*	Median (IQR): $6.32 (3.16 – 13.68)				
***Shin, 2020*[** **71** **]**	*Patient OOP*	Inpatient (Initial)		Mean (SD): $30.31 (39.54)												Inpatient (Initial)	Mean (SD): $36.59 (71.19)				
		Inpatient (Recurrent)		Mean (SD): $46.31 (55.63)																	
		Outpatient (HIV)		Mean (SD): $6.58 (10.25)																	
		Outpatient (TB)		Mean (SD): $3.42 (2.86)												Inpatient (Recurrent)	Mean (SD): $143.44 (262.60)				
						*Self-Reported OOP*	Inpatient (Initial)		Mean (SD): $11.59 (26.42)												
							Inpatient (Recurrent)		Mean (SD): $21.92 (55.93)												
		Outpatient (HIV)		Mean (SD): $1.70 (3.91)												Outpatient (HIV)	Mean (SD):$3.18 (11.93)				
							Outpatient (TB)		–												
						*Total OOP*	Inpatient (Initial)		Mean (SD): $58.06 (58.93)												
		Inpatient (Recurrent)		Mean (SD): $80.38 (93.27)												Outpatient (TB)	Mean (SD): $3.42 (2.84)				
							Outpatient (HIV)		Mean: $6.58 (10.25)												
							Outpatient (TB)		Mean (SD): $3.42 (2.86)												
***Stracker, 2019*[** **72** **]**	*TB* ^+^	Mean: $99.45																			
	*Xpert-*	Mean: $15.65																			
***Sweeney, 2018*** ^*^ **[** **73** **]**					Mean: $23.32					Mean: $37.97						*Approach #1: Current income (prompted ranges)*	Mean (SD): $34.58 (55.15)				
																*Approach #2: Current income (detailed)*	Mean (SD): $45.18 (55.81)				
																*Approach #3: Permanent income (MCA)*	Mean (SD): $77.55 (80.52)				
																*Approach #4: National mean income*	Mean (SD): $118.02 (99.55)				
																*Approach #5: Self-reported income loss*	Mean (SD): $89.06 (771.91)				
***Trajman, 2016*[** **76** **]**	*All*	*Minimum Wage*		Mean (SD): $75.43 (116.28)	*Minimum Wage*		Mean (SD): $64.43 (112.35)									*All*	*Minimum Wage*		Mean (SD): $248.28 (1,151.83)		
																	*SES Income per Capita*		Mean (SD): $248.28 (1,154.19)		
		*Reported Income*		Mean (SD): $89.57 (127.28)													*Income per Activity*		*Mean (SD):* $260.85 (1,156.54)		
																	*Reported Income*		Mean (SD): $280.49 (1,559.61)		
	*Patients Only*	*Minimum Wage*		Mean (SD): $40.07 (58.14)	*Reported Income*		Mean (SD): $82.50 (124.14)									*Patients Only*	*Minimum Wage*		*Mean (SD):* $243.57 (1,151.83)		
																	*SES Income per Capita*		*Mean (SD):* $245.92 (1,154.19)		
		*Reported Income*		Mean (SD): $44.78 (60.50)													*Income per Activity*		Mean (SD): $253.78 (1,155.76)		
																	*Reported Income*		Mean (SD) $278.14 (1,556.46)		
***Viney, 2021*[** **81** **]**					*Extra-pulmonary TB*		Median (IQR): $63.72 (0.00 – 83.49)			*Extra-pulmonary TB*				Median (IQR): $550.39 (232.90 – 1,453.43)		*Extra-pulmonary TB*	Median (IQR): $108.76 (0.00 – 283.44)				
					*Pulmonary TB*		Median (IQR): $39.55 (0.00 – 75.80)			*Pulmonary TB*				Median (IQR): $433.94 (134.03 – 907.43)		*Pulmonary TB*	Median (IQR); $81.30 (0.00 – 365.83)				
					*Total*		Median (IQR): $40.65 (0.00 – 78.00)			*Total*				Median (IQR): $477.89 (160.39 – 1,055.74)		*Total*	Median (IQR): $81.30 (0.00 – 325.18)				
***Wang, 2020*[** **84** **]**					*Direct medical costs*		Mean: $5,979.64			Mean: $384.94						Mean: $1,965.00					
							Median (IQR): $5,256.12 (3,643.81 – 8,293.30)														
					*OOP medical costs*		Mean: $3,743.57			Median (IQR): $350.68 (168.28 – 544.15)						Median (IQR): $1,200.16 (29.22 – 3,275.00)					
																Median (IQR): $3,159.11 (1,496.42 – 5,996.77)					
***Yamanaka, 2024***^***(1)***^ **[86]**					People with TB		Mean (95%CI): $3.75 (0.71- 6.89)			People with TB			Mean (95%CI): $61.92 (54.11- 69.72)			People with TB	Mean (95%CI): $596.15 (496.24- 696.17)				
					People with TB-DM		Mean (95%CI): $2.63 (0.0- 6.28)			People with TB-DM			Mean (95%CI): $119.37 (90.69- 148.05)			People with TB-DM	Mean (95%CI): $553.19 (354.87- 751.50)				
					Total		Mean (95%CI): $3.65 (0.91- 6.38)			Total			Mean (95%CI): $69.52 (61.61- 77.52)			Total	Mean (95%CI): $590.48 (499.88 -(681.07)				
***No Stratifications***																					
***Chandra, 2021*** ^***(2)***^ **[** **19** **]**	Median (IQR): $13.68 (1.20 – 71.58)				Median (IQR): $0 (0.00 – 0.00)					Median (IQR): $13.68 (1.20 – 66.32)						Median (IQR): $0.00 (0.00 – 110.53)					
	Mean (SD): $45.26 (60.00)				Mean (SD): $1.79 (11.16)					Mean (SD): $43.16 (58.95)						Mean (SD): $120.00 (242.11)					
***Getahun, 2016*[** **37** **]**	Mean (SD): $39.67 (41.67)															Mean (SD): $69.52 (53.53)					
	Median (R): $38.91 (486.33)															Median (R): $51.68 (72.96)					
***Kilale, 2022*[** **42** **]**					Mean (SD): $32.13 (158.34)											Mean (SD): $87.39 (697.18)					
					Median (IQR): 0.00 (0.00 – 4.61)											Median (IQR): $18.42 (6.91 – 41.89)					
***Kirubi, 2021*[** **43** **]**					Median (IQR): $10.29 (0.00 – 41.18)					Median (IQR): $161.91 (73.00 – 322.89)						Median (IQR): $33.69 (20.59 – 61.77)					
***Loureiro, 2024* [** **44** **]**	Mean: $63.03				Mean: $4.42					Mean: 58.62						Mean: $1,119.87					
***Muniyandi, 2020*[** **58** **]**	Mean (SD): $54.03 (205.02)															Mean (SD): $256.69 (542.04)					
	Median (IQR): $17.17 (0.00 – 3.52)															Median (IQR): $0.00 (0.00 – 4,894.33)					
***Ukwaja, 2013*** ^***(1)***^ **[** **77** **]**	Mean (SD): $49.62 (60.04)															Mean (SD): $49.62 (47.76)					
***Ukwaja, 2013*** ^***(2)***^ **[** **78** **]**	Median (IQR): $33.12 (29.77 – 52.11)																				
***Viney, 2019*[** **80** **]**					Mean (95% CI): $268.53 (27.65 – 509.39)					Mean (95% CI): $1,731.39 (1,537.25 – 1,925.52)						Mean (95% CI): $1,276.08 (990.26 – 1,561.90)					

**Abbreviations: *TB – Tuberculosis, DS-TB – Drug sensitive TB, DR-TB – Drug-resistant TB, MDR-TB – Multi-drug resistant TB, RS-TB – Rifampicin sensitive TB, RMR-TB – Rifampicin mono-resistant TB, HIV – Human Immunodeficiency Virus, LTBI – Latent TB Infection, CHC – Community health centre, ACF – Active case finding, PCF – Passive case finding, SES – Socioeconomic status, DOT – Directly observed therapy, SD – Standard deviation, IQR – Interquartile range, CI – Confidence Interval.***

*Costs reported are a combination of pre- and post-diagnostic costs.

Non-medical costs ranged from a mean of $7.60 to $8,649 while median costs ranged from $1.41 to $520 (S6 Table). Key factors that impacted non-medical cost burden were food, transportation, and nutritional supports. MDR-TB patients consistently incurred the largest burden of non-medical costs except in the 2020 study by Chittamany *et al*., where DS-TB patients incurred greater costs than DR-TB patients (median: DS-TB: $520, DR-TB: $423)[23].

Indirect costs ranged from a mean of $1.26 to $6,045 while median costs ranged from $1.57 to $2,837 (S7 Table). Loss of time was the largest component cost contributing to overall lost income (mean $0.18 to $6,045, median $0.69 to $950). Indirect costs were highest amongst DR-TB patients (mean: $51.46-$6,045, median: $80.89-$2,837).

### Patient cost variation by geographical region

Across WHO regions, weighted total costs were highest in DR-TB patients from the WPRO region with a median of $8,774 and a mean of $8,481 (Table 5). Pre-diagnostic and post-diagnostic weighted medians could only be calculated for two WHO regions in DR-TB patients – AFRO and SEARO. Weighted medians for both stages were higher in the AFRO region and lower in the SEARO region (pre-diagnostic: $104 versus $14.88; post-diagnostic: $1,619 versus $746). Weighted means for the pre- and post-diagnostic stages could not be calculated due to insufficient data.

In high TB burden settings, weighted medians for total costs incurred by DR-TB patients were identical in high TB and MDR-TB burden settings ($8,774) while means were higher in high MDR-TB burden settings compared to high TB/HIV burden settings ($4,184 versus $2,853). For pre-diagnostic and post-diagnostic stages, weighted medians were highest in high TB burden settings and lowest in high MDR-TB settings.

In low TB burden settings, medians for total costs incurred by DR-TB patients were highest in low TB/HIV burden settings compared to low MDR-TB burden settings ($3,568 versus $1,722). Weighted medians for pre-diagnostic costs were only able to be calculated for low MDR-TB settings. Post-diagnostic costs were highest in low MDR-TB settings and identical for both low DS-TB and low TB/HIV settings ($1,619 versus $1,110). Weighted means for low TB burden settings could not be obtained due to insufficient data.

**Table 5:** Subgroup analysis on the weighted median and means for pre-diagnostic, post-diagnostic and total costs incurred by DR-TB/MDR-TB patients during TB care.

	Pre-Diagnostic Cost		Post-Diagnostic Cost		Total Cost	
***Median***						
***WHO Regions***						
*AFRO*	N=2	$103.74	N=3	$1,618.58	N=3	$1,722.32
*EMRO*	N=0	–	N=0	–	N=0	–
*SEARO*	N=2	$14.88	N=2	$746.00	N=2	$1,044.23
*WPRO*	N=0	–	N=0	–	N=4	$8,774.10
*PAHO*	N=0	–	N=0	–	N=0	–
***High TB Burden***						
*High TB*	N=3	$93.76	N=3	$1,618.58	N=7	$8,774.10
*High MDR-TB*	N=2	$14.88	N=2	$1,110.38	N=6	$8,774.10
*High TB/HIV*	N=3	$14.88	N=3	$1,142.78	N=8	$6,422.72
***Low TB Burden***						
*Low TB*	N=0	–	N=2	$1,110.38	N=2	$2,913.84
*Low MDR-TB*	N=2	$93.76	N=3	$1,618.58	N=4	$1,722.32
*Low TB/HIV*	N=0	–	N=2	$1,110.38	N=2	$3,568.11
***Mean***						
***WHO Region***						
*AFRO*	N=0	–	N=0	–	N=5	$991.84
*EMRO*	N=0	–	N=0	–	N=0	–
*SEARO*	N=0	–	N=0	–	N=0	–
*WPRO*	N=0	–	N=0	–	N=6	$8,480.85
*PAHO*	N=0	–	N=0	–	N=0	–
***High TB Burden***						
*High TB*	N=0	–	N=0	–	N=10	$3,176.47
*High MDR-TB*	N=0	–	N=0	–	N=8	$4,183.77
*High TB/HIV*	N=0	–	N=0	–	N=8	$2,852.59
***Low TB Burden***						
*Low TB*	N=0	–	N=0	–	N=0	–
*Low MDR-TB*	N=0	–	N=0	–	N=3	$304.80
*Low TB/HIV*	N=0	–	N=0	–	N=3	$2,730.31

Abbreviations: *WHO – World Health Organization, AFRO – African region, EMRO – Eastern Mediterranean region, SEARO – South-East Asia region, WPRO – Western Pacific region, PAHO – Pan American region, TB – Tuberculosis, MDR-TB – Multidrug resistant TB, HIV – Human Immunodeficiency Virus.*

*Studies with N=1 included costs and population sizes for different subgroups within the patient population.

**Table 6:** Subgroup analysis on the weighted median and means for pre-diagnostic, post-diagnostic and total costs incurred by DS-TB patients during TB care.

	Pre-Diagnostic Cost		Post-Diagnostic Cost		Total Cost	
***Median***						
***WHO Regions***						
*AFRO*	N=3	$20.89	N=4	$130.60	N=8	$266.64
*EMRO*	N=0	–	N=0	–	N=2	$122.00
*SEARO*	N=7	$42.00	N=6	$49.80	N=11	$88.80
*WPRO*	N=0	–	N=0	–	N=6	$1,189.90
*PAHO*	N=0	–	N=0	–	N=2	$389.17
***High TB Burden***						
*High TB*	N=7	$51.08	N=6	$60.44	N=17	$148.40
*High MDR-TB*	N=9	$51.08	N=7	$60.44	N=17	$201.28
*High TB/HIV*	N=6	$42.00	N=5	$60.44	N=13	$206.89
***Low TB Burden***						
*Low TB*	N=5	$33.60	N=6	$49.80	N=12	$245.20
*Low MDR-TB*	N=4	$20.89	N=5	$130.60	N=11	$149.05
*Low TB/HIV*	N=7	$51.08	N=7	$91.00	N=13	$148.40
***Mean***						
***WHO Region***						
*AFRO*	N=2	$38.16	N=3	$45.24	N=13	$297.58
*EMRO*	N=0	–	N=0	–	N=0	–
*SEARO*	N=3	$166.28	N=4	$236.13	N=7	$1,148.91
*WPRO*	N=3	$353.26	N=3	$1,058.77	N=8	$1,708.79
*PAHO*	N=1^*^	$173.96	N=1^*^	$602.43	N=3	$204.71
***High TB Burden***						
*High TB*	N=7	$284.25	N=8	$654.22	N=23	$925.07
*High MDR-TB*	N=6	$287.79	N=7	$745.20	N=16	$1,218.98
*High TB/HIV*	N=5	$556.95	N=5	$1,196.72	N=17	$731.46
***Low TB Burden***						
*Low TB*	N=0	–	N=0	–	N=8	$910.32
*Low MDR-TB*	N=3	$47.09	N=3	$59.56	N=13	$576.82
*Low TB/HIV*	N=3	$15.03	N=4	$415.95	N=11	$1,348.45

Abbreviations: *WHO – World Health Organization, AFRO – African region, EMRO – Eastern Mediterranean region, SEARO – South-East Asia region, WPRO – Western Pacific region, PAHO – Pan American region, TB – Tuberculosis, MDR-TB – Multidrug resistant TB, HIV – Human Immunodeficiency Virus.*

*Studies with N=1 included costs and population sizes for different subgroups with the patient population.

Total costs of care for DS-TB patients were highest in the WPRO region (median: $1,190, mean: $1,709) (Table 6). Median weighted averages for pre- and post-diagnostic stages varied between regions while mean weighted averages were consistently higher in the WPRO region for both stages (mean pre-diagnostic: $353, post-diagnostic: $1,059).

### Catastrophic costs

Based on the WHO definition, the proportion of patients that incurred catastrophic costs while seeking care ranged from 3% to 98% (Table 7) with MDR-TB patients reporting a higher burden of catastrophic costs (62-98%) compared with DS-TB patients (28-62%). In addition, patients identified via ACF reported a lower burden of catastrophic costs compared to patients identified via PCF, (15-45% versus 30-61%).

**Table 7:** Proportion of patients that suffered from catastrophic costs and their cut-off point.

*Study*	*Cut-Off Point*	*Catastrophic Costs*				
***Aia, 2022* [** **14** **]**	*20%*	*DS_TB*			*MDR-TB*	*Total*
		*32.9%*			*84.2%*	*33.9% (95% CI: 31.0 – 36.9)*
	*30%*	*24.9%*			*73.7%*	*25.8% (95% CI:32.1 – 28.6)*
	*40%*	*19.9%*			*68.4%*	*20.8% (95% CI:18.3 – 23.4)*
	*50%*	*14.9%*			*68.4%*	*15.9% (95% CI:13.7 – 18.3)*
	*60%*	*8.7%*			*68.4%*	*9.8% (95% CI:8.0 – 11.8)*
	*70%*	*7.2%*			*63.2%*	*8.3% (95% CI:6.7 – 10.2)*
***Assebe, 2020* [** **15** **]**	*10%*	*DS-TB*		*40%*		
		*DR-TB*		*62%*		
***Aung, 2021* [** **16** **]**	*20%*	*60%*				
***Chandra, 2021*** ^***(1)***^ **[19]**	*20%*	*18% (95% CI 12 – 27%)*				
***Chatterjee, 2023* [** **22** **]**	*20%*	*35%*				
***Chittamany, 2020* [** **23** **]**	*20%*	*Total*		*62.6%*		
		*DS-TB*		*62.2%*		
		*DR-TB*		*86.7%*		
		*TB/HIV*		*81.1%*		
***Diallo, 2022* [** **27** **]**	*20%*	*Total*		*54% (95% CI: 46 – 62)*		
		*DS-TB*		*54.4% (95% CI: 46.4 – 62.0)*		
		*DR-TB*		*50.0% (95% CI: 16.4 – 83.6)*		
	*25%*	*48% (95% CI: 41 – 54)*				
	*30%*	*41% (95% CI: 34 - 48)*				
	*35%*	*33% (95% CI: 26 – 41)*				
	*40%*	*28% (95% CI: 21 – 36)*				
***Devoid, 2022* [** **29** **]**	*20%*	*3%*				
***Ellaban, 2021* [** **31** **]**	*20%*	*33%*				
***Florentino. 2022* [** **32** **]**	*20%*	*Total*		*42.4% (95% CI: 40.2 – 44.6)*		
		*DS-TB*		*41.7% (95% CI: 39.3 – 44.1)*		
		*DR-TB*		*89.7% (95% CI: 86.3 – 93.0)*		
***Fuady, 2020*** ^***(1)***^ **[33]**	*10%*	*46%*				
	*15%*	*38%*				
	*20%*	*33%*				
	*25%*	*26%*				
	*30%*	*22%*				
	*35%*	*17%*				
***Fuady, 2018* [** **35** **]**	*20%*	*DS-TB*		*36%*		
		*MDR-TB*		*83%*		
***Gadallah, 2022* [** **36** **]**	*10%*	*59.9%*				
	*15%*	*42.8%*				
	*20%*	*20.1%*				
	*25%*	*13.2%*				
	*30%*	*6.6%*				
***Getahun, 2016* [** **37** **]**	*40%*	*63%*				
***Gurung, 2019* [** **40** **]**	*20%*	*ACF*		*45%*		
		*PCF*		*61%*		
		*Total*		*53%*		
***Gurung, 2021* [** **39** **]**	*20%*	*32%*				
***Kilale, 2022* [** **42** **]**	*20%*	*Total*		*44.9% (95% CI: 41.3 – 48.4)*		
		*DS-TB*		*43.7% (95% CI: 40.1 – 47.3)*		
		*DR-TB*		*80.0% (95% CI: 59.2 – 93.1)*		
***Kirubi, 2021* [** **43** **]**	*20%*	*27%*				
***Lu, 2020* [** **45** **]**	*20%*	*22.2%*				
***Manyazewal, 2022* [** **49** **]**	*20%*	*54.4% (95% CI: 40.7 – 67.6)*				
***McAllister, 2020* [** **54** **]**	*10%*	*38.6%*				
	*20%*	*26.5%*				
	*25%*	*21.7%*				
***Morishita, 2016* [** **56** **]**		*ACF*		*PCF*		
	*10%*	*54.6%*		*63%*		
	*20%*	*36.1%*		*45%*		
	*30%*	*24.1%*		*34%*		
	*40%*	*17.6%*		*21%*		
***Mudzengi, 2017* [** **57** **]**		*TB/HIV*			*TB*	*HIV*
	*5%*	*73%*			*55%*	*72%*
	*10%*	*67%*			*53%*	*60%*
	*15%*	*65%*			*47%*	*55%*
	*20%*	*64%*			*47%*	*52%*
	*25%*	*61%*			*45%*	*49%*
***Muniyandi, 2020* [** **58** **]**	*20%*	*31%*				
***Muttamba, 2020* [** **59** **]**	*20%*	*53.1%*				
***Nhung, 2018* [** **61** **]**		*MDR-TB*			*DS-TB*	*Total*
	*20%*	*98%*			*59.6%*	*63%*
	*30%*	*98%*			*43%*	*48%*
	*40%*	*89%*			*30%*	*35%*
***Pedrazzoli, 2021* [** **64** **]**	*20%*	*Insured*			*65%*	
		*Uninsured*			*59%*	
		*Total*			*64%*	
***Pedrazzoli, 2018* [** **63** **]**	*20%*	*64.1%*				
***Prasanna, 2018* [** **66** **]**	*10%*	*49%*				
	*20%*	*32.5%*				
***Rupani, 2022* [** **70** **]**	*20%*	*TB*		*4% (95% CI: 2 – 8)*		
		*TB/HIV*		*12% (95% CI: 8 – 16)*		
***Rupani, 2020* [** **69** **]**	*20%*	*4%*				
***Stracker, 2019* [** **72** **]**	*20%*	*28%*				
***Sweeney, 2018* [** **73** **]**	*20%*	*0-36%*				
***Timire, 2020* [** **74** **]**	*20%*	*80%*				
***Tomeny, 2020* [** **75** **]**	*20%*	*DS-TB*		*28%*		
		*MDR-TB*		*80%*		
***Ukwaja, 2013***^***(2)***^ **[78]**	*5%*	*OOP Expenditure*		*95%*		
	*10%*			*65%*		
	*15%*			*37%*		
	*25%*			*9%*		
	*40%*	*Non-food Expenditure*		*44%*		
***Viney, 2019* [** **80** **]**	*20%*	*83%*				
***Viney, 2021* [** **81** **]**	*20%*	*Total*		*82% (95% CI: 76.3 – 87.6)*		
		*Extra-pulmonary TB*		*86.1% (95% CI: 74.2 – 98.0)*		
		*Pulmonary TB*		*81% (95% CI: 74.5 – 87.4)*		
***Vo, 2021* [** **82** **]**	*20%*	*ACF*		*15%*		
		*PCF*		*30%*		
***Walcott, 2020* [** **83** **]**	*20%*	*41.8%*				
***Wang, 2020* [** **84** **]**	*20%*	*CHE*		*63.8%*		
		*CTC*		*87%*		
***Yang, 2020* [** **88** **]**		*RS*	*RMR*		*MDR*	*Total*
	*15%*	*43.3%*	*46.7%*		*73.2%*	*46%*
	*20%*	*33.6%*	*43.3%*		*69.6%*	*37.1%*
	*25%*	*26.3%*	*40%*		*66.1%*	*30.2%*

Abbreviations: *TB – Tuberculosis, DS-TB – Drug sensitive TB, DR-TB – Drug-resistant TB, MDR-TB – Multi-drug resistant TB, RS-TB – Rifampicin sensitive TB, RMR-TB – Rifampicin mono-resistant TB, HIV – Human Immunodeficiency Virus, LTBI – Latent TB Infection, ACF – Active case finding, PCF – Passive case finding, CHE – Catastrophic health expenditure, CTC – Catastrophic total cost, OOP – Out of pocket*

To help offset the costs of TB care, patients who suffered from catastrophic costs reported using coping strategies such as: borrowing money (reported in n=33 studies, range: 0-94%), selling assets (n=27, range: 0-52%), using savings (n=11, range: 10-40%), or a combination of strategies (n=10, range: 12-81%) (S8 Table).

### Risk of bias reporting checklist

**Fig 3:**
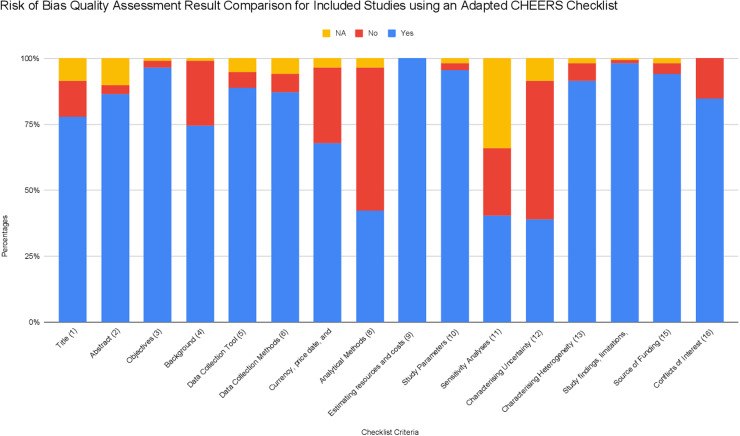
Shown above is the Risk of Bias assessment done using an adapted CHEERS checklist. This figure highlights the key checklist criteria categories for the adapted checklist and reflects the number of studies that meet the criteria (blue), don’t meet the criteria (red), or where the criteria was not applicable.

A risk of bias quality assessment was done on all included studies using an adapted CHEERS checklist, results are displayed in Fig 3, studies scored from 55-96% on the reporting checklist. Overall, studies included in this review were found to be of high quality with 63% of studies (n=48/76) scoring above 70% of checklist items indicating a low risk of bias. There were some key areas of concern, including missing information on currency used, price data, and conversion information, as well as information pertaining to analytical methods, and sensitivity analysis.

## Discussion

Eliminating the burden of catastrophic costs experienced by individuals combatting TB is one of the WHO End TB Strategy targets, this systematic review summarized current evidence from 76 studies published from 2011 to 2024 regarding the costs incurred by individuals diagnosed with and receiving TB treatment and identified main drivers of costs to inform mitigation strategies.

A surge in research in the last five years has led to an increase in TB costing studies conducted across a range of patient populations in low-, middle-, and high-income settings. Total TB care costs ranged widely however, when analyzed based on TB type it was found that DR-TB patients incurred considerably higher costs compared to DS-TB patients. This was consistently seen when examining the *average total cost of TB care* as well as the *weighted total mean/median cost of TB care*. In patients with DS-TB, there was also a large stratification across differing patient populations. Analysis of the *average total costs of TB care* for DS-TB patients found that costs were highest in initial TB patients, patients identified using PCF, and PLHIV despite this review identifying limited cost data for PLHIV [25–29].

Overall, direct medical and indirect costs were the main drivers of financial burden for patients. Direct medical costs including patient consultations, medication, diagnostic imaging, and hospitalization, in addition to indirect costs, namely loss of income were the largest component costs. Pre-diagnostic costs were found to be lower than post-diagnostic costs, despite previous literature finding that patients incurred a larger burden of costs during the pre-diagnostic stage[3,90]. When analyzed based on patient type, little difference in pre-diagnostic costs was seen between DR-TB and DS-TB patients. During the pre-diagnostic stage, indirect costs and direct medical costs had the largest burden on patients. Post-diagnostic costs saw DR-TB patients incurring a larger burden compared to DS-TB patients and once again, direct medical costs and indirect costs had the largest impact on patients. Our findings support previous literature that income loss is one of many factors that poses a significant financial risk for patients and their families while receiving TB care[3,91]. Supporting individuals throughout their TB treatment either through basic income support or cash transfers may be an important element in ensuring individuals can complete treatment and do not face further financial hardship.

Despite similarities between study populations and settings, there were large variations in patient-incurred costs. For DS-TB and DR-TB patients, weighted total costs were highest in WPRO countries including Cambodia, China, Lao People’s Democratic Republic, the Philippines and Viet Nam[92]. As expected, due to the complicated nature of DR-TB, weighted averages were highest in countries with high MDR-TB burden like China, India, and Vietnam where patients often incur a larger burden of costs associated with medication, severe adverse events and challenges with treatment adherence[93–95]. Financial compensation strategies like unconditional or conditional (e.g., for patients with specific types of TB) cash transfers, have shown promising results for reducing the costs burden, although additional data is needed[4,96,97]. “One-size-fits-all” health care policies fall short when it comes to TB care. Countries must adopt tailored policies that provide support and care to their most vulnerable populations [88,98].

The findings of this review highlighted some key patient subgroups of interest for further analysis, Evidence shows that PLHIV had some of the lowest weighted averages, despite previous literature predicting that these individuals may suffer additional financial burdens associated with HIV care, more data on patient level costs among PLHIV is warranted in helping to understand the interplay between costs incurred during TB and HIV care and how catastrophic costs could be mitigated in this group [29,57,70,99,100]. Persons diagnosed with DR-TB, receiving their initial dose of treatment, or identified through PCF consistently incurred larger burden of costs compared to other subgroups, likely due to requirements of longer treatment, longer hospitalizations, more complicated treatment regimens, and higher risk of severe adverse events[101]. ACF is an effective tool to promote earlier TB case detection, and may not only lead to improved patient outcomes, and potentially reduce ongoing community transmission but our review suggests it may also serve to reduce the burden of costs patients incur due to delayed TB diagnosis and lengthier treatment trajectories. In addition, the inclusion of novel, shortened treatment regimens can help to reduce the duration of treatment for both DS-TB and DR-TB patients and as a result, limit the burden of costs that these individuals and their families may face. Studies have found that bedaquiline, pretomanid, and linezolid (BPaL) based regimens are considered to be more effective than the current standard of care for TB treatment and are most cost effective for both individuals as well as the health system[102–104].

Most individuals were found to incur catastrophic costs while receiving TB care. Catastrophic costs were consistently higher in patients with MDR-TB or identified via PCF. These catastrophic costs lead patients to use coping mechanisms such as taking out loans, selling assets or using their savings to help offset some of the financial burden of TB care, leading to significant social consequences such as job loss, interrupted work or schooling, and food insecurity and may impact relationships and result in social exclusion [16,61,69,105]. These factors can isolate individuals from their community and further perpetuate poverty, such that patients may hesitate to seek care to limit these consequences, thereby increasing TB transmission[106–108]. Patients who incur catastrophic costs are 2-4 times more likely to experience treatment failure [34], therefore mitigating these costs will not only benefit individuals but also the health systems and communities at large. ACF programs coupled with improved access to DST such as targeted next generation sequencing could have an important impact on reducing catastrophic costs.

### Study strengths and limitations

There are several strengths to this review; including use of a comprehensive search strategy encompassing studies with differing patient and geographical populations over a large time frame and considered all income level country settings allowing us to compile and compare an extensive range of data. One key limitation is that we restricted to studies that collected patient costing data using WHO or WHO-adapted patient costing surveys. This precluded studies that were conducted prior to the development of the costing survey and studies that did not use the WHO patient costing survey from inclusion, however this approach allowed for improved comparability across included studies. Another key limitation of this study is that some studies conducted after 2019 were restricted in their data collection due to the COVID-19 pandemic and due to the global impact of this pandemic, patients reported suffering from higher cost burdens. As a result, the costs estimated for these studies may not accurately represent the burden of costs that patients in these regions incur outside of the pandemic.

### Conclusion

The findings of this review have demonstrated that “free” TB care under national TB programs is not adequate to prevent patients from facing severe financial burdens during TB diagnosis and treatment. Throughout all stages of care, patients incur costs which vary largely based on patient type and geographical location. These costs can be catastrophic, resulting in the use of various coping mechanisms. Complementary social protection and financial support policies and systems should be further developed and implemented alongside standard TB care programs to protect TB patients from financial burden. Furthermore, uptake of practices like active case finding and more effective treatment regimens like BPaL can help to identify and treat individuals in a cost-savings, timely manner. By working to understand the factors that influence patient costs, we can develop tailored social protection strategies and policies that provide effective TB care resources while lowering catastrophic costs for patients in accordance with the WHO “End TB Strategy” goals[98,109].

## Supporting information

S1 Checklist PRISMA checklist. (DOCX)

S1 Text Search strategy for all databases. (DOCX)

S1 File Studies screened with exclusion reasons. (XLSX)

S1 Table Key definitions table. (DOCX)

S1 Fig Temporal breakdown of included studies. (DOCX)

S2 Fig Geographical breakdown of included studies. (DOCX)

S3 Fig Average total cost of TB care by country (mean, median). (DOCX)

S2 Table Breakdown of direct medical costs incurred by patients during the pre-diagnostic phase. (DOCX)

S3 Table Breakdown of direct non-medical costs incurred by patients during the pre-diagnostic phase. (DOCX)

S4 Table Breakdown of indirect costs incurred by patients during the pre-diagnostic phase. (DOCX)

S5 Table Breakdown of direct medical cost incurred by patients during the post-diagnostic phase. (DOCX)

S6 Table Breakdown of direct non-medical costs incurred by patients during the post-diagnostic phase. (DOCX)

S7 Table Breakdown of indirect costs incurred by patients during the post-diagnostic phase. (DOCX)

S8 Table Use of coping strategies in the included studies. (DOCX)
