# Supplementary figures and images for: The catastrophic cost of TB care: Understanding costs incurred by individuals undergoing TB care in low-, middle-, and high-income settings – A systematic review
Source: PLOS Glob Public Health. 2025 Apr 2;5(4):e0004283. doi: 10.1371/journal.pgph.0004283 (PMC12005564; doi:10.1371/journal.pgph.0004283)

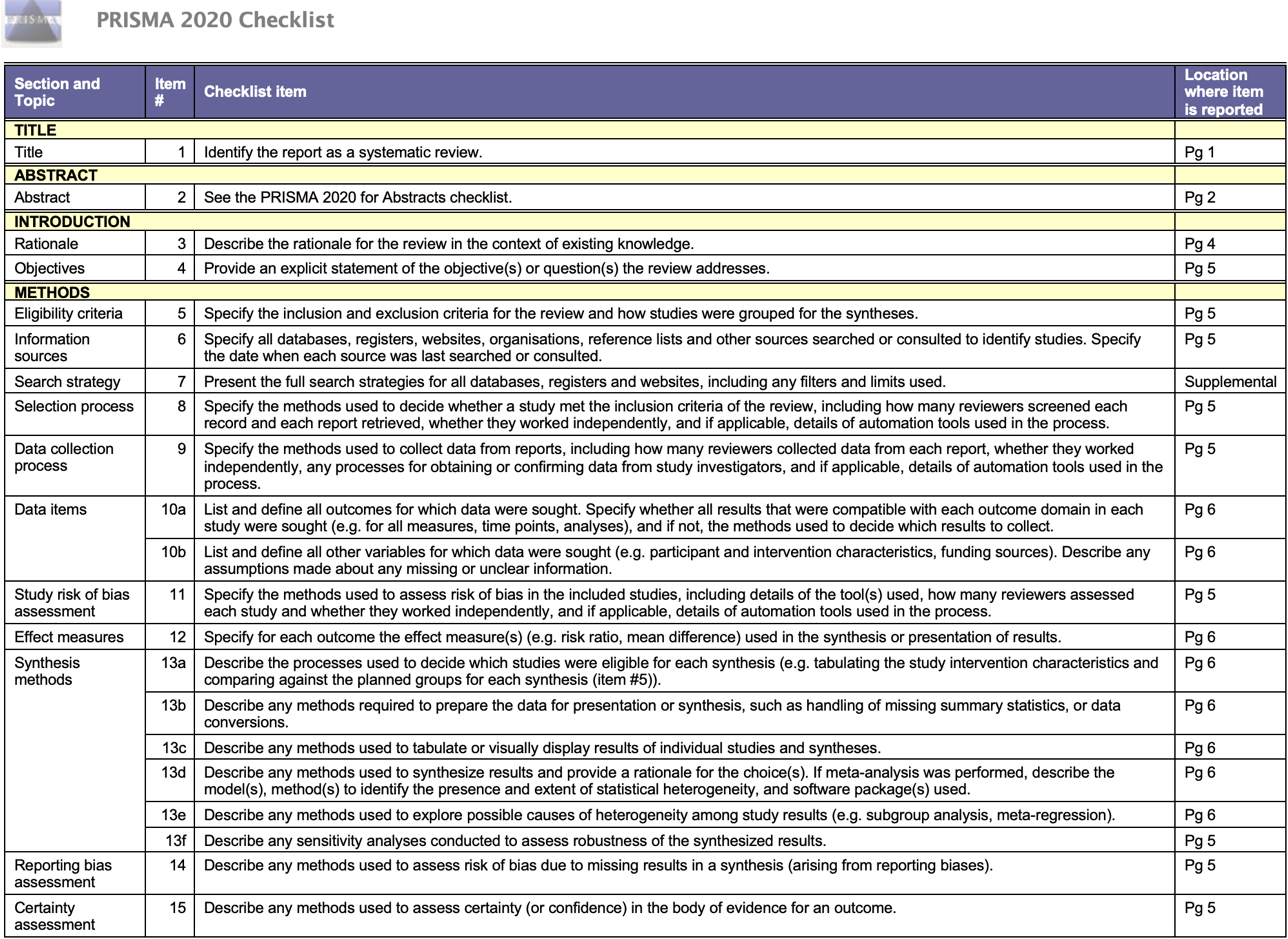

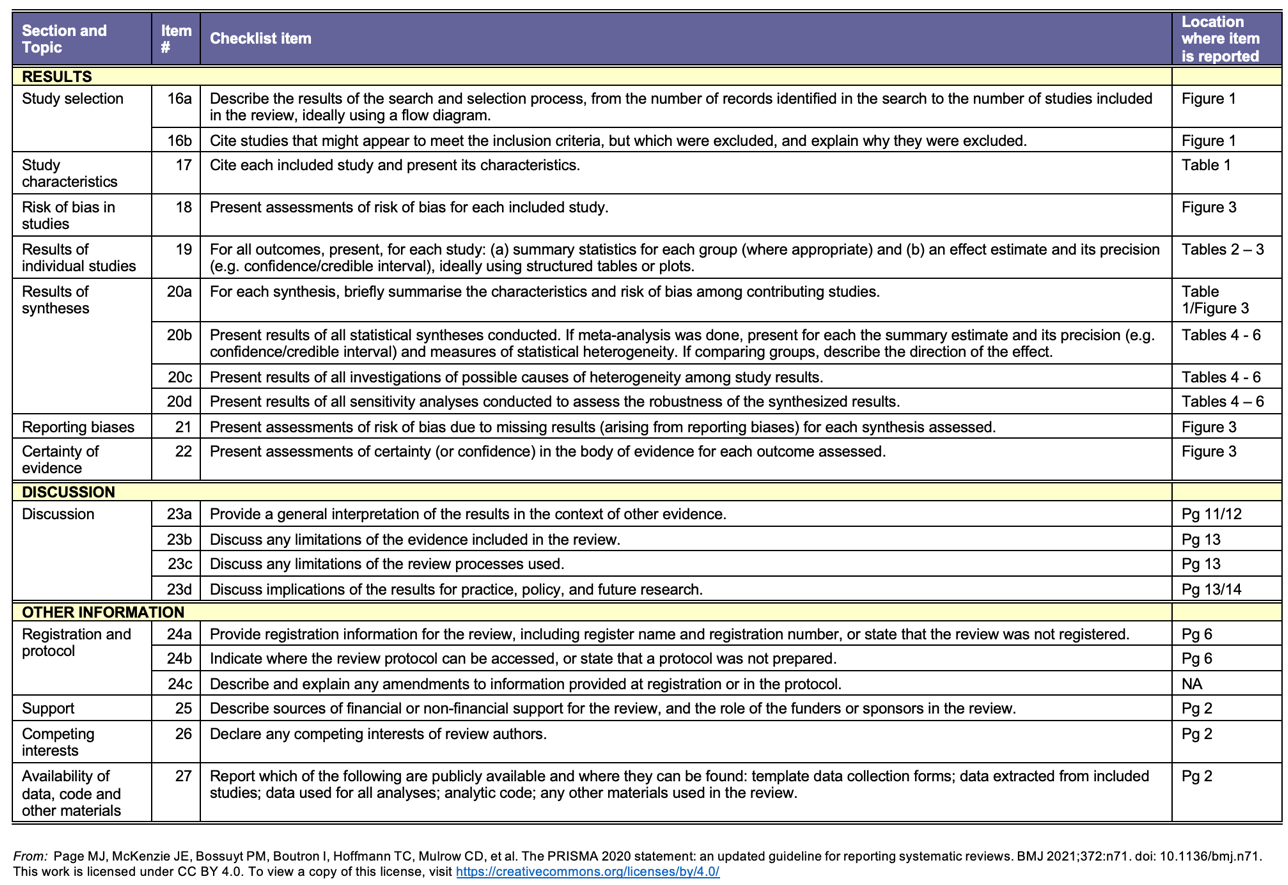

Supplement: S1 Checklist — (DOCX) [file pgph.0004283.s001.docx]

## ***Figure S.3 – Average total cost of TB care by country for median and mean costs***

*
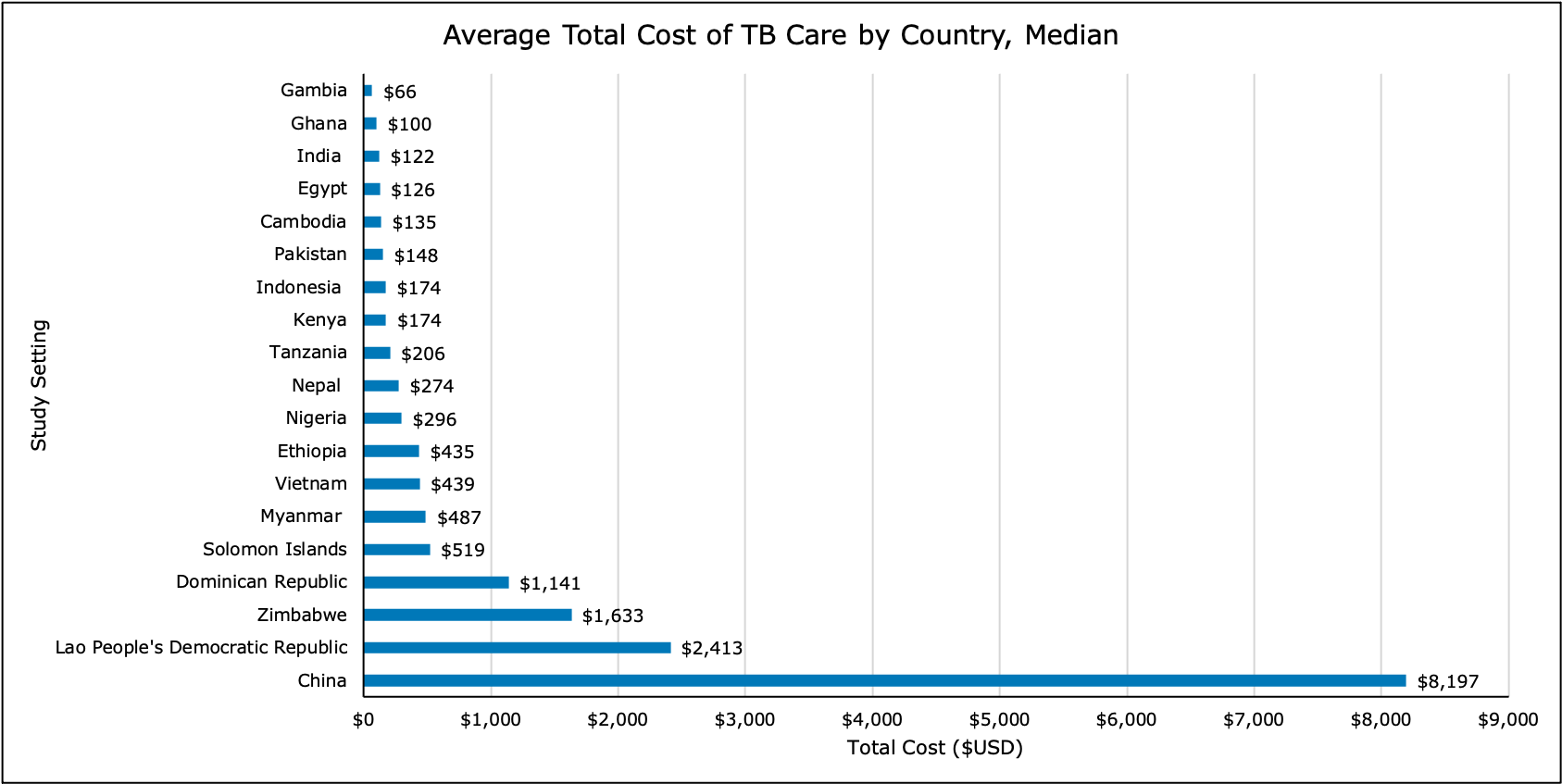
*

Supplement: S3 Fig — (DOCX) [file pgph.0004283.s007.docx]
