# Supplementary material for: The catastrophic cost of TB care: Understanding costs incurred by individuals undergoing TB care in low-, middle-, and high-income settings – A systematic review
Source: PLOS Glob Public Health. 2025 Apr 2;5(4):e0004283. doi: 10.1371/journal.pgph.0004283 (PMC12005564; doi:10.1371/journal.pgph.0004283)
Supplement: S1 Text — (DOCX) [file pgph.0004283.s002.docx]

# ***Search Strategy:***

*Medline and Embase (OVID):*

- 1. Latent Tuberculosis/ or Tuberculosis/ or Mycobacterium tuberculosis/ or Tuberculosis, Multidrug-Resistant/
- 2. tuberculos*.ti,ab,kw.
- 3. (latent adj1 tuberculos*).ti,ab,kw.
- 4. (latent adj2 tuberculos*).ti,ab,kw.
- 5. (latent adj2 infection*).ti,ab,kw.
- 6. (mycobacterium adj1 tuberculos*).ti,ab,kw.
- 7. (mycobacterium adj2 infection*).ti,ab,kw.
- 8. (mdr adj1 tuberculos*).ti,ab,kw.
- 9. (multi* adj2 tuberculos*).ti,ab,kw.
- 10. 1 or 2 or 3 or 4 or 5 or 6 or 7 or 8 or 9
- 11. patient cost*.ti,ab,kw.
- 12. catastrophic cost*.ti,ab,kw.
- 13. 11 or 12
- 14. 10 and 13

*Web of Science/ Web of Knowledge:*

- 1. (Tuberculosis OR TB OR Mycobacterium) (Topic)
- 2. (“Latent tuberculos*” OR “latent TB infection*”) (Topic)
- 3. (MDR* TB” OR “MDR* Tuberculos*” OR “Multidrug-resistant Tuberculos*”) (Topic)
- 4. (1 OR 2 OR 3) (Topic)
- 5.(“Patient cost*” OR “catastrophic cost*”) (Topic)
- 6. (3 AND 5)

*Scopus:*

- 1. (Tuberculos* OR TB OR mycobacterium) (title/abstract/keywords)
- 2. (“Latent tuberculos*” OR “latent TB infection*” OR LTBI) (title/abstract/keywords)
- 3. (“MDR* TB” OR “MDR* Tuberculos*” OR “Multidrug-resistant Tuberculos*”) (title/abstract/keywords)
- 4. (“Patient cost*” or “catastrophic cost*”) (title/abstract/keywords)
- 5. ((1 OR 2 OR 3) AND 4) (title/abstract/keywords)
