# Supplementary material for: The catastrophic cost of TB care: Understanding costs incurred by individuals undergoing TB care in low-, middle-, and high-income settings – A systematic review
Source: PLOS Glob Public Health. 2025 Apr 2;5(4):e0004283. doi: 10.1371/journal.pgph.0004283 (PMC12005564; doi:10.1371/journal.pgph.0004283)
Supplement: S1 Table — (DOCX) [file pgph.0004283.s004.docx]

## ***Table S.1 – Definitions of Key Costing Terms.***

| *Term* | *Definition* | *Source* |
| --- | --- | --- |
| *Total costs* | Costs incurred throughout the whole TB care process including pre- and post-diagnostic costs | ^[1,3,9]^ |
| *Pre-diagnostic costs* | Costs incurred from symptom onset to TB diagnosis | ^[1,3,9]^ |
| *Post-diagnostic costs* | Costs incurred following TB diagnosis to treatment completion | ^[1,3,9]^ |
| *Direct medical costs* | Costs pertaining to medical services, examinations, and treatment | ^[5,14,15]^. |
| *Direct non-medical costs* | Costs involved in but not pertaining to accessing treatment (i.e., accommodations, transportation, food, etc.) | ^[14,15]^ |
| *Indirect costs* | Costs associated with a reduction in time and productivity at work or any form of paid labour due to illness | ^[14,16]^ |
| *Catastrophic costs* | TB related costs exceeding more than 20% of a households annual income pre-TB diagnosis | ^[5]^ |
| *Income Loss* | Income loss consists both of lost wages due to inability to work and income lost to premature death | ^[62]^ |
