# Supplementary material for: The catastrophic cost of TB care: Understanding costs incurred by individuals undergoing TB care in low-, middle-, and high-income settings – A systematic review
Source: PLOS Glob Public Health. 2025 Apr 2;5(4):e0004283. doi: 10.1371/journal.pgph.0004283 (PMC12005564; doi:10.1371/journal.pgph.0004283)
Supplement: S1 Fig — (DOCX) [file pgph.0004283.s005.docx]

## ***Figure S.1 – Temporal Breakdown of Included Studies by Year of Publication***

**Note that 2024 only accounts for partial year data*
