# Supplementary material for: The catastrophic cost of TB care: Understanding costs incurred by individuals undergoing TB care in low-, middle-, and high-income settings – A systematic review
Source: PLOS Glob Public Health. 2025 Apr 2;5(4):e0004283. doi: 10.1371/journal.pgph.0004283 (PMC12005564; doi:10.1371/journal.pgph.0004283)
Supplement: S2 Fig — (DOCX) [file pgph.0004283.s006.docx]

## ***Figure S.2 – Geographical Breakdown of Included Studies by Continent***
