# Supplementary material for: The catastrophic cost of TB care: Understanding costs incurred by individuals undergoing TB care in low-, middle-, and high-income settings – A systematic review
Source: PLOS Glob Public Health. 2025 Apr 2;5(4):e0004283. doi: 10.1371/journal.pgph.0004283 (PMC12005564; doi:10.1371/journal.pgph.0004283)
Supplement: S2 Table — (DOCX) [file pgph.0004283.s008.docx]

## ***Table S2 – Breakdown of the direct medical costs incurred by patients during the pre-diagnostic stage of TB care.***

|  | *Total* | | | *Consultation* | | *Medication* | | *Diagnostic Imaging* | | *Follow-up Tests* | | *Hospitalization* | | *Other* | | |
| --- | --- | --- | --- | --- | --- | --- | --- | --- | --- | --- | --- | --- | --- | --- | --- | --- |
| *Aia, 2022* | DS-TB | | Mean (95% CI): *$9.40(7.20 – 11.50)* |  | |  | |  |  |  |  |  | |  | | |
|  | MDR-TB | | Mean (95% CI): *$16.30 (9.20 – 41.80)* |  |  |  |  |  |  |  |  |  |  |  |  |  |
|  | Total | | Mean (95% CI): *$9.50 (7.40 – 11.60)* |  |  |  |  |  |  |  |  |  |  |  |  |  |
| *Assebe, 2020^18^* |  | | |  | |  | | *Outpatient* | Mean (SD) - $2 (5) | *Outpatient* | Mean (SD) - $8 (13) |  | |  | | |
|  |  |  |  |  |  |  |  |  | Median (IQR) - $1 (0-2) |  | Median (IQR) - $4 (0-12) |  |  |  |  |  |
|  |  |  |  |  |  |  |  | *Inpatient* | Mean (SD) - $11 (16) | *Inpatient* | Mean (SD) - $14 (16) |  |  |  |  |  |
|  |  |  |  |  |  |  |  |  | Median (IQR) - $7 (1-14) |  | Median (IQR) - $7 (3-18) |  |  |  |  |  |
|  |  |  |  |  |  |  |  | *Total* | Mean (SD) - $3 (7) | *Total* | Mean (SD) - $9 (15) |  |  |  |  |  |
|  |  |  |  |  |  |  |  |  | Median (IQR) - $1 (0-2) |  | Median (IQR) - $5 (0-13) |  |  |  |  |  |
| *Aung, 2021^19^* | *MDR-TB* | Median: $19.31 | |  | |  | |  | |  | |  | |  | | |
|  | *DS-TB* | Median: $11.01 | |  |  |  |  |  |  |  |  |  |  |  |  |  |
|  | *Total* | Median: $11.01 | |  |  |  |  |  |  |  |  |  |  |  |  |  |
| *Chandra, 2021 ^(1)21^* | Median (IQR): $23.3 (7.9-50.7) | | |  | |  | |  | |  | |  | |  | | |
| *Chandra, 2021 ^(2) 22^* | Median (IQR): $24 (8.1-57*)* | | | Median (IQR): $0.43 (0-5.6) | | Median (IQR): $2.8 (0 - 12) | |  | | Median (IQR): $ 5.6 (0-19) | | Median (IQR): $ 0 (0) | |  | | |
|  | Mean (SD): $65 (153) | | | Mean (SD): $3.4 (5) | | Mean (SD): $11 (22) | |  |  | Mean (SD): $16 (28) | | Mean (SD): $28 (126) | |  |  |  |
| *Chatterjee, 22023202320232023* |  | | | Mean: $4,883.38 | | *Mean: $0.00* | |  | |  | | *Mean: $3,199.61* | |  | | |
| *Chittamany, 2020^23^* | *DS-TB* | *Median: $63.63* | | *DS-TB* | Median: $60.45 |  | |  | |  | | *DS-TB* | *Median: $6.36* |  | | |
|  | *DR-TB* | *Median: $112.94* | | *DR-TB* | Median: $112.94 |  |  |  |  |  |  | *DR-TB* | *Median: $9.54* |  |  |  |
|  | *Total* | *Median: $63.63* | | *Total* | Median: $60.45 |  |  |  |  |  |  | *Total* | *Median: $6.36* |  |  |  |
| *Diallo, 2022* | DS-TB | | Mean (95% CI): $38.60 (24.13 – 53.13) |  | |  | |  | |  | |  | |  | | |
|  | DR-TB | | Mean (95% CI): $41.40 (-7.30 – 90.10) |  |  |  |  |  |  |  |  |  |  |  |  |  |
|  | Total | | Mean (95% CI): $38.70 (24.35 – 53.01) |  |  |  |  |  |  |  |  |  |  |  |  |  |
| *De Siqueria Filha, 2018^25^* | *TB/HIV* | Mean: $26.94 | | *TB/HIV* | Mean: $14.94 | *TB/HIV* | *Mean: $5.65* |  | | *TB/HIV* | *Mean: $6.35* |  | |  | | |
|  | *LTBI/HIV* | *Mean: $3.7* | | *LTBI/HIV* | Mean: $1.30 | *LTBI/HIV* | *Mean: $0.03* |  |  | *LTBI/HIV* | Mean: $2.43 |  |  |  |  |  |
| *Devoid, 2022* |  | | | Mean (SD): $1.12 (2.82) | | *Mean (SD): $14.17 (25.08)* | | *Mean (SD): $1.19 (2.21)* | | *Mean (SD): $1.84 (4.59)* | | *Mean (SD): $1.26 (17.34)* | | *Other Procedures* | | |
| *Ellaban, 2021^26^* | *Median (IQR): $15.6 (6.3-26.3)* | | |  | |  | |  | |  | |  | |  | | |
| *Florentino, 2022* | Urban DS-TB | | Mean (SD): $16.20 (16.90) |  | |  | |  | |  | |  | |  | |  |
|  | Rural DS-TB | | Mean (SD): $16.40 (27.90) |  |  |  |  |  |  |  |  |  |  |  |  |  |
|  | DR-TB | | Mean (SD): $20.00 (208.60) |  |  |  |  |  |  |  |  |  |  |  |  |  |
|  | Total | | Mean (SD): $16.40 (38.80) |  |  |  |  |  |  |  |  |  |  |  |  |  |
| *Gurung, 2019^33^* | *ACF* | *Median: $15.52* | | ACF | Median: $0 | *ACF* | *Median: $6.40* | *ACF* | *Median: $0.54* | *ACF* | Median: $1.19 |  | | *Medical Supplies* | *ACF* | *Median: $0* |
|  | *PCF* | *Median: $34.30* | | PCF | Median: $0.22 | *PCF* | *Median: $19.86* | *PCF* | *Median: $3.47* | *PCF* | *Median: $2.93* |  |  |  | *PCF* | *Median: $3.58* |
|  | *Total* | *Median: 20.84* | | Total | Median: $0 | *Total* | *Median: $8.79* | *Total* | *Median: $1.74* | *Total* | *Median: $1.95* |  |  |  | *Total* | *Median: $2.9* |
| *Gurung, 2021^32^* | *ACF* | *Mean (95% CI): $41.1 (28.7–53.6)* | |  | |  | |  | |  | |  | |  | | |
|  |  | *Median (IQR): $12.3 (0–55.8)* | |  |  |  |  |  |  |  |  |  |  |  |  |  |
|  | *PCF* | *Mean (95% CI): $53.1(41.6–64.6)* | |  |  |  |  |  |  |  |  |  |  |  |  |  |
|  |  | *Median (IQR): $29.6 (10.2–79.2)* | |  |  |  |  |  |  |  |  |  |  |  |  |  |
|  | *Total* | *Mean (95% CI): $47.2 (38.8–55.7)* | |  |  |  |  |  |  |  |  |  |  |  |  |  |
|  |  | *Median (IQR): $21.7 (3.5–70.3)* | |  |  |  |  |  |  |  |  |  |  |  |  |  |
| *Kaswa, 2021* | DS-TB | | Mean (95% CI): $13.50 (11.10 – 16.00) |  | |  | |  | |  | |  | |  | | |
|  | DR-TB | | Mean (95% CI): $23.00 (14.30 – 31.80) |  |  |  |  |  |  |  |  |  |  |  |  |  |
|  | Total | | Mean (95% CI): $15.30 (12.30 – 18.20) |  |  |  |  |  |  |  |  |  |  |  |  |  |
| *Kilale, 2022* | Mean (SD): $10.30 (15.90) | | |  | |  | |  | |  | |  | |  | | |
|  | Median (IQR); $7.20 (7.20 – 7.20) | | |  |  |  |  |  |  |  |  |  |  |  |  |  |
| *Loureiro,2024* | Mean: $55.24 | | |  | | Mean: $44.80 | | Mean: $3.91 | | Mean: $6.53 | |  | |  | | |
| *Lu, 2020^35^* | *Residents* | Mean: $919.70 | |  | | *Residents* | *Mean: $5,213.79* | *Residents* | *Mean: $731.45* |  | |  | | *Medical Supplies* | *Residents* | *Mean: $422.68* |
|  | *Migrants* | Mean: $1,908.65 | |  |  | *Migrants* | *Mean: $417.88* | *Migrants* | *Mean: $425.38* |  |  |  |  |  | *Migrants* | *Mean: $79.44* |
| *Mauch, 2013^(1) 36^* |  | | | *Ghana* | Mean: $0.44 | *Ghana* | *Mean: $1.76* | *Ghana* | *Mean:0.44* | *Ghana* | *Mean: $0.15* |  | |  | | |
|  |  |  |  |  | Median: $0.00 |  | *Median: $0.59* |  | *Median (IQR): $0 (0 – 0.44)* |  | *Median (IQR): $0 (0 – 0)* |  |  |  |  |  |
|  |  |  |  | *Vietnam* | Mean: $1.80 | *Vietnam* | *Mean: $5.84* | *Vietnam* | *Mean: $2.47* | *Vietnam* | *Mean: $10.55* |  |  |  |  |  |
|  |  |  |  |  | Median: $0.45 |  | *Median: $2.70* |  | *Median (IQR): $0.67 (0.40 – 1.32)* |  | Median (IQR): $2.02 (0.92 – 10.57) |  |  |  |  |  |
|  |  |  |  | *Dominican Republic* | Mean: $2.81 | *Dominican Republic* | *Mean: $0.40* | *Dominican Republic* | *Mean: $3.42* | *Dominican Republic* | *Mean: $1.21* |  |  |  |  |  |
|  |  |  |  |  | Median: $0.00 |  | *Median: $0.00* |  | *Median (IQR): $0 (0 – 1.11)* |  | *Median (IQR): $0 (0 - 0.08)* |  |  |  |  |  |
| *Mauch, 2013 ^(2) 38^* |  | | | New | Median: $7.48 | New | *Median: $14. 97* |  | |  | |  | |  | | |
|  |  |  |  | Retreatment | Median: $1.00 | Retreatment | *Median: $31.29* |  |  |  |  |  |  |  |  |  |
|  |  |  |  | MDR-TB | Median: $20.02 | MDR-TB | *Median: $9.38* |  |  |  |  |  |  |  |  |  |
| *Mauch, 2011^37^* |  | | |  | |  | | *Pulmonary TB* | *Median: $2.92* |  | |  | |  | | |
|  |  |  |  |  |  |  |  | *Extrapulmonary TB* | *Median: $4.93* |  |  |  |  |  |  |  |
| *McAllister, 2020^39^* |  | | | *CHC* | *Median (IQR): $3.39 (1.52 – 6.89)* | *CHC* | *Median (IQR): $ 5.56 (* | *CHC* | *Median (IQR): $ 5.18* | *CHC* | *Median (IQR): $ 5.07* | *CHC* | *Median (IQR): $25.33* |  | | |
|  |  |  |  | *Public Hospital* | *Median (IQR): $5.44 (2.53 – 8.11)* | *Public Hospital* | *Median (IQR): $5.86* | *Public Hospital* | *Median (IQR): $4.56* | *Public Hospital* | *Median (IQR): $3.80* | *Public Hospital* | *Median (IQR): $40.53* |  |  |  |
|  |  |  |  | *Private Hospital* | *Median (IQR): $5.83 (1.52 – 11.65)* | *Private Hospital* | *Median (IQR): $7.08* | *Private Hospital* | *Median (IQR): $7.85* | *Private Hospital* | *Median (IQR): $9.13* | *Private Hospital* | *Median (IQR): $43.07* |  |  |  |
|  |  |  |  | *Private Practice* | *Median (IQR): $5.07 (2.03 – 10.13)* | *Private Practice* | *Median (IQR): $7.67* | *Private Practice* | *Median (IQR): $6.51* | *Private Practice* | *Median (IQR): $ 6.40* | *Private Practice* | *Median (IQR): $48.64* |  |  |  |
| *Morishita, 2016^40^* |  | | | *ACF* | Mean (SD): $0.82 | *ACF* | Mean (SD): $3.88 | *ACF* | Mean (SD): $0.31 | *ACF* | Mean (SD): $  0.36 | *ACF* | Mean (SD): $0.05 |  | | |
|  |  |  |  |  | Median (IQR): $0 (0 – 0.10) |  | Median (IQR): $0.00 |  | Median (IQR): $  0.00 |  | Median (IQR): $  0.00 |  | Median (IQR): $0.00 |  |  |  |
|  |  |  |  | *PCF* | Mean (SD): $0.05 | *PCF* | Mean (SD): $18.66 | *PCF* | Mean (SD): $1.07 | *PCF* | Mean (SD): $  1.17 | *PCF* | Mean (SD): $1.89 |  |  |  |
|  |  |  |  |  | Median (IQR): $0 (0-0) |  | *Median (IQR): $*3.16 |  | Median (IQR): $0.00 |  | Median (IQR): $  0.00 |  | Median (IQR): $0.00 |  |  |  |
| *Muttamba, 2020^43^* | DS-TB | Mean (95% CI): $  3.42 | |  | |  | |  | |  | |  | |  | | |
|  | MDR-TB | Mean (95% CI): $  1.65 | |  |  |  |  |  |  |  |  |  |  |  |  |  |
|  | Total | Mean (95% CI): $  3.40 | |  |  |  |  |  |  |  |  |  |  |  |  |  |
| *Nhung, 2018^44^* | *DS-TB* | Mean (95% CI): $  175.71 | |  | |  | |  | |  | |  | |  | | |
|  | *MDR-TB* | Mean (95% CI): $  680.89 | |  |  |  |  |  |  |  |  |  |  |  |  |  |
|  | *Total* | Mean (95% CI): $  276.75 | |  |  |  |  |  |  |  |  |  |  |  |  |  |
| *Pedrazzoli, 2018^45^* | *DS-TB* | *Median (IQR): $*  8.11 | |  | |  | |  | |  | |  | |  | | |
|  | *MDR-TB* | *Median (IQR): $*  8.42 | |  |  |  |  |  |  |  |  |  |  |  |  |  |
|  | *Total* | *Median (IQR): $*  8.11 | |  |  |  |  |  |  |  |  |  |  |  |  |  |
| *Pedrazzoli, 2021^46^* | *Insured* | *Mean: $*  13.68 | |  | |  | |  | |  | |  | |  | | |
|  |  | *Median (IQR): $*  8.21 | |  |  |  |  |  |  |  |  |  |  |  |  |  |
|  | *Uninsured* | *Mean: $*  8.81 | |  |  |  |  |  |  |  |  |  |  |  |  |  |
|  |  | *Median (IQR): $*  8.21 | |  |  |  |  |  |  |  |  |  |  |  |  |  |
| *Razzaq, 2022* | Median (IQR): $25.00 (12.10 – 35.00) | | |  | |  | |  | |  | |  | |  | | |
| *Ramma, 2015^48^* |  | | | *Inpatient* | *Mean (SD): $0.66 (3.78)* |  | |  | |  | |  | |  | | |
|  |  |  |  |  | *Median: $0* |  |  |  |  |  |  |  |  |  |  |  |
|  |  |  |  | *Outpatient* | *Mean (SD): $*  0.52  (2.08) |  |  |  |  |  |  |  |  |  |  |  |
|  |  |  |  |  | *Median: $0* |  |  |  |  |  |  |  |  |  |  |  |
| *Timire, 2021^53^* | DS-TB | Median (IQR):  $20.41 | |  | |  | |  | |  | |  | |  | | |
|  | DR-TB | Median (IQR):  $10.61 | |  |  |  |  |  |  |  |  |  |  |  |  |  |
|  | Total | Median (IQR):  $20.41 | |  |  |  |  |  |  |  |  |  |  |  |  |  |
| *Ukwaja, 2013 ^(1) 56^* |  | | |  | | *Non-TB Medication* | *Mean (SD):* $13.23 | *Mean (SD):* $4.76 | | *Mean (SD):* $5.82 | |  | |  | | |
| *Viney, 2019^59^* | Mean (SD):  $7.17 | | |  | |  | |  | |  | |  | |  | | |
| *Viney, 2022* | Extra-pulmonary TB | | Median (IQR): $0.00 (0 -0) |  | |  | |  | |  | |  | |  | | |
|  | Pulmonary TB | | Median (IQR): $0.00 (0 – 0) |  |  |  |  |  |  |  |  |  |  |  |  |  |
|  | Total | | Median (IQR): $0.00 (0 – 0) |  |  |  |  |  |  |  |  |  |  |  |  |  |
| *Vo, 2021* | ACF | | Mean (95% CI): $37.00 (18.00 – 56.00) |  | |  | |  | |  | |  | |  | | |
|  |  |  | Median (IQR): $13.00 (4.00 – 31.00) |  |  |  |  |  |  |  |  |  |  |  |  |  |
|  | PCF | | Mean (95% CI): $151.00 (70.00 – 231.00) |  |  |  |  |  |  |  |  |  |  |  |  |  |
|  |  |  | Median (IQR): $75.00 (30.00 – 168.00) |  |  |  |  |  |  |  |  |  |  |  |  |  |
|  | Total | | Mean (95% CI): $90.00 (50.00 – 130.00) |  |  |  |  |  |  |  |  |  |  |  |  |  |
|  |  |  | Median (IQR): $29.00 (11.00 – 81.00) |  |  |  |  |  |  |  |  |  |  |  |  |  |
| *Walcott, 2020^60^* | Mean (SD):  $13.61 | | |  | | *Non-TB Medication* | Mean (SD):  $11.21 | Mean (SD):  $2.40 | |  | |  | |  | | |
|  | Median (IQR):  $7.61 | | |  |  |  | Median (IQR):  $4.40 | *Median (IQR):*  $1.20 | |  |  |  |  |  |  |  |
| *Abbreviations: TB – Tuberculosis, DS-TB – Drug sensitive TB, MDR-TB – Multi-drug resistant TB, DR-TB – Drug resistant TB, RS-TB – Rifampicin sensitive TB, RMR-TB – Rifampicin mono-resistant TB, HIV – Human Immunodeficiency Virus, LTBI – Latent TB Infection, CHC – Community health centre, ACF – Active case finding, PCF – Passive case finding, SD – Standard deviation, IQR – Interquartile range, CI – Confidence Interval* | | | | | | | | | | | | | | | | |
