# Supplementary material for: The catastrophic cost of TB care: Understanding costs incurred by individuals undergoing TB care in low-, middle-, and high-income settings – A systematic review
Source: PLOS Glob Public Health. 2025 Apr 2;5(4):e0004283. doi: 10.1371/journal.pgph.0004283 (PMC12005564; doi:10.1371/journal.pgph.0004283)
Supplement: S3 Table — (DOCX) [file pgph.0004283.s009.docx]

## ***Table S3 – Breakdown of the direct non-medical costs incurred by patients during the pre-diagnostic phase of TB care***

|  | *Total* | | *Transportation* | | *Accommodation* | | *Food* | | *Nutritional Supplements* | | *Other* | | |
| --- | --- | --- | --- | --- | --- | --- | --- | --- | --- | --- | --- | --- | --- |
| *Aia, 2022* | DS-TB | Mean (95% CI): *$23.20 (20.20 – 26.20)* |  | |  | |  | |  | |  | | |
|  | MDR-TB | Mean (95% CI): *$41.70 (21.40 – 76.80)* |  |  |  |  |  |  |  |  |  |  |  |
|  | Total | Mean (95% CI): *$23.50 (20.50 – 26.50)* |  |  |  |  |  |  |  |  |  |  |  |
| *Chandra, 2021 ^(1) 21^* | *Median (IQR): $3.2 (1.1 - 10.7)* | |  | |  | |  | |  | |  | | |
| *Chandra, 2021 ^(2) 22^* | Median (IQR): $3.2 (1.1 - 10.7) | | Median (IQR): $3.1 (1.1 - 8.2) | | Median (IQR): $0 (0) | | *Food during travel* | Median (IQR): $0 (0-0.71) | Median (IQR): $0 (0-0) | |  | | |
|  |  |  |  |  |  |  |  | Mean (SD): $1.1 (2.8) |  |  |  |  |  |
|  | Mean (SD): $16 (70.5) | | Mean (SD): $7 (8.9) | | Mean (SD): $7 (69) | | *Special diet* | Median (IQR): $0 (0) | Mean (SD): $0 (0) | |  |  |  |
|  |  |  |  |  |  |  |  | Mean (SD): $0.74 (3.9) |  |  |  |  |  |
| *Chatterjee, 2023* |  | |  | |  | | *General Population* | *Mean: $0.00* |  | |  | | |
| *Chittamany, 2020^23^* | *DS-TB* | *Median: $17.50* | *DS-TB* | *Median: $6.36* | *DS-TB* | *Median: $0* | *DS-TB* | Median: $1.59 |  | |  | | |
|  | *DR-TB* | *Median: $63.63* | *DR-TB* | *Median: $ 11.14* | *DR-TB* | *Median: $0* | *DR-TB* | Median: $12.73 |  |  |  |  |  |
|  | *Total* | *Median: $17.50* | *Total* | *Median: $6.36* | *Total* | *Median: $0* | *Total* | Median: $1.59 |  |  |  |  |  |
| *Diallo. 2022* | DS-TB | Mean (95% CI): $9.1 (4.93 - 13.3) |  |  |  | |  |  |  | |  | | |
|  | DR-TB | Mean (95% CI): $2.7 (1.61 - 3.78) |  |  |  |  |  |  |  |  |  |  |  |
|  | Total | Mean (95% CI): $8.99 (4.88 - 13.1) |  |  |  |  |  |  |  |  |  |  |  |
| *De Siqueria Filha, 2018^25^* |  | | *TB/HIV* | *Mean - $12.90* |  | | *TB/HIV* | *Mean - $6.07* |  | |  | | |
|  |  |  | *LTBI/HIV* | *Mean - $2.98* |  |  | *LTBI/HIV* | Mean - $10.47 |  |  |  |  |  |
| *Devoid, 2022* |  | | *Mean (SD): $3.49 (6.66)* | |  | | *Mean (SD): $0.81 (2.03)* | |  | |  | | |
| *Ellaban, 2021^26^* |  | | Median (IQR): $5.6 (1.3-12.5) | |  | | Median (IQR): $0.0 (0.0-3.8) | |  | |  | | |
| *Florentino. 2022* | Urban DS-TB | Mean (SD): $2.60 (4.90) |  | |  | |  | |  | |  | | |
|  | Rural DS-TB | Mean (SD): $3.10 (7.80) |  |  |  |  |  |  |  |  |  |  |  |
|  | DR-TB | Mean (SD): $6.50 (76.80) |  |  |  |  |  |  |  |  |  |  |  |
|  | Total | Mean (SD): $3.10 (10.60) |  |  |  |  |  |  |  |  |  |  |  |
| *Gurung, 2019^33^* | ACF | Median: $3.69 | ACF | Median: $3.58 |  | | ACF | NA |  | |  | | |
|  | PCF | Median: $10.53 | PCF | Median: $5.86 |  |  | PCF | Median: $0 |  |  |  |  |  |
|  | Total | Median: $5.86 | Total | Median: $4.02 |  |  | Total | Median: $0 |  |  |  |  |  |
| *Gurung, 2021^32^* | *ACF* | Mean (95% CI): $6.8 (3.7–9.9) |  | |  | |  | |  | |  | | |
|  |  | Median (IQR): $1.4 (0–5.8) |  |  |  |  |  |  |  |  |  |  |  |
|  | *PCF* | Mean (95% CI): $18.4 (11.9–24.8) |  |  |  |  |  |  |  |  |  |  |  |
|  |  | Median (IQR): $5.3 (1.8–14.1) |  |  |  |  |  |  |  |  |  |  |  |
|  | *Total* | Mean (95% CI): $12.7 (9.0–16.4) |  |  |  |  |  |  |  |  |  |  |  |
|  |  | Median (IQR): $3.0 (0.4–10.8) |  |  |  |  |  |  |  |  |  |  |  |
| *Kaswa, 2021* | DS-TB | Mean (95% CI): $2.10 (1.60 – 2.70) | DS-TB | *Mean (95% CI): $0.80 (0.50 – 1.00)* | DS-TB | *Mean (95% CI): $1.00 (0.70 -1.40)* | DS-TB | *Mean (95% CI): $0.10 (0.00 – 0.20)* | DS-TB | *Mean (95% CI): $0.10 (0.10 – 0.20)* |  |  | |
|  | DR-TB | Mean (95% CI): $ 3.50 (1.40 – 5.70) | DR-TB | *Mean (95% CI): $1.50 (0.90 – 2.20)* | DR-TB | *Mean (95% CI): $2.10 (0.50 – 3.70)* | DR-TB | *Mean (95% CI): $0.10 (0.00 – 0.20)* | DR-TB | *Mean (95% CI): $0.10 (0.00 – 0.20)* |  |  |  |
|  | Total | Mean (95% CI): $2.40 (1.60 – 3.20) | Total | *Mean (95% CI): $0.90 (0.60 – 1.20)* | Total | *Mean (95% CI): $1.20 (0.70 – 1.70)* | Total | *Mean (95% CI): $0.10 (0.00 – 0.10)* | Total | *Mean (95% CI): $0.10 (0.10 – 0.20)* |  |  |  |
| *Kilale, 2022* | Mean (SD): $4.20 (6.80) | |  | |  | |  | |  | |  |  | |
|  | Median (IQR): $3.00 (3.00 – 3.00) | |  |  |  |  |  |  |  |  |  |  |  |
| *Loureiro, 2024* | Mean: $25.43 | | Mean: $16.65 | | Mean: $0.82 | | Mean: $6.14 | |  | | *Administrative* | Mean: $1.81 | |
| *Lu, 2020^35^* | *Residents* | *Mean: $167.78* |  | |  | |  | |  | | *Residents* | *Mean: $139.09* | |
|  | *Migrants* | *Mean: $79.44* |  |  |  |  |  |  |  |  | *Migrants* | *Mean: $64.75* | |
| *Mauch, 2013^(1) 36^* |  | | *Ghana* | *Mean: $0.59* | *Ghana* | *Mean: $0.29* | *Ghana* | *Mean: $0.88* |  | |  | | |
|  |  |  |  | *Median (IQR): $0.15 (0 – 0.59)* |  | *Median (IQR): $0 (0-0)* |  | *Median (IQR): $0.15 (0 – 0.59)* |  |  |  |  |  |
|  |  |  | *Vietnam* | *Mean: $1.35* | *Vietnam* | *Mean: $7.18* | *Vietnam* | *Mean: $6.06* |  |  |  |  |  |
|  |  |  |  | *Median (IQR): $0.45 (0.27 – 0.79)* |  | *Median (IQR): $6.51 (1.98 – 13.20)* |  | *Median (IQR): $0.67 (0.27 – 6.60)* |  |  |  |  |  |
|  |  |  | *Dominican Republic* | *Mean: $0.40* | *Dominican Republic* | *Mean: $0* | *Dominican Republic* | *Mean: $0.40* |  |  |  |  |  |
|  |  |  |  | *Median (IQR): $ 0.16 (0.12 – 0.56)* |  | *Median (IQR): $0 (0-0)* |  | *Median (IQR): $0.12 (0 – 0.28)* |  |  |  |  |  |
| *Mauch, 2013 ^(2) 38^* |  | | New | Median: $1.53 | New | Median: $0 | New | Median: $1.53 |  | |  | | |
|  |  |  | Retreatment | Median: $1.26 | Retreatment | Median: $0 | Retreatment | Median: $1.26 |  |  |  |  |  |
|  |  |  | MDR-TB | Median: $1.26 | MDR-TB | Median: $0 | MDR-TB | Median: $0.90 |  |  |  |  |  |
| *McAllister, 2020^39^* |  | | *CHC* | *Median (IQR): $3.55* |  | | *CHC* | *Median (IQR): $1.26* |  | |  | | |
|  |  |  | *Public Hospital* | *Median (IQR): $6.41* |  |  | *Public Hospital* | *Median (IQR): $2.02* |  |  |  |  |  |
|  |  |  | *Private Hospital* | *Median (IQR): $4.12* |  |  | *Private Hospital* | *Median (IQR): $2.03* |  |  |  |  |  |
|  |  |  | *Private Practice* | *Median (IQR): $5.19* |  |  | *Private Practice* | *Median (IQR): $1.01* |  |  |  |  |  |
| *Morishita, 2016^40^* |  | | *ACF* | Mean (SD): $  0.97 |  | | *ACF* | Mean (SD): $  0.20 |  | |  | | |
|  |  |  |  | Median (IQR): $  0.61 |  |  |  | Median (IQR): $  0.00 |  |  |  |  |  |
|  |  |  | *PCF* | Mean (SD): $  3.68 |  |  | *PCF* | Mean (SD): $  1.07 |  |  |  |  |  |
|  |  |  |  | Median (IQR): $  0.92 |  |  |  | Median (IQR): $  0.00 |  |  |  |  |  |
| *Muttamba, 2020^43^* |  | | DS-TB | Mean (95% CI): $  0.84 | DS-TB | Mean (95% CI): $  0.14 | DS-TB | Mean (95% CI): $  0.44 | DS-TB | Mean (95% CI): $  75.68 |  | | |
|  |  |  | MDR-TB | Mean (95% CI): $  2.59 | MDR-TB | Mean (95% CI): $  0.00 | MDR-TB | Mean (95% CI): $  0.28 | MDR-TB | Mean (95% CI): $  505.73 |  |  |  |
|  |  |  | Total | Mean (95% CI): $  0.86 | Total | Mean (95% CI): $  0.14 | Total | Mean (95% CI): $  0.44 | Total | Mean (95% CI): $  90.09 |  |  |  |
| *Nhung, 2018^44^* | *DS-TB* | Mean (95% CI): $  175.71 |  | |  | |  | |  | |  | | |
|  | *MDR-TB* | Mean (95% CI): $  724.81 |  |  |  |  |  |  |  |  |  |  |  |
|  | *Total* | Mean (95% CI): $219.64 |  |  |  |  |  |  |  |  |  |  |  |
| *Pedrazzoli, 2018^45^* | *DS-TB* | *Median (IQR): $*  0.88 |  | |  | |  | |  | |  | | |
|  | *MDR-TB* | *Median (IQR): $*  0.88 |  |  |  |  |  |  |  |  |  |  |  |
|  | *Total* | *Median (IQR): $*  0.88 |  |  |  |  |  |  |  |  |  |  |  |
| *Ramma, 2015^48^* |  | | *Inpatient* | *Mean (SD): $2.50* |  | |  | | *Inpatient* | Mean (SD): $5.95 |  | *Inpatient* | Mean (SD): $  3.21 |
|  |  |  |  | *Median: $0* |  |  |  |  |  | Median (IQR): $  0.71 |  |  | Median (IQR): $0 |
|  |  |  | *Outpatient* | *Mean (SD): $1.70* |  |  |  |  | *Outpatient* | Mean (SD): $  23.25 |  | *Outpatient* | Mean (SD): $  0.99 |
|  |  |  |  | *Median (IQR): $0* |  |  |  |  |  | Median (IQR):  19.85 |  |  | Median: $0 |
| *Razzaq, 2022* | Median (IQR): $5.20 (4.30 – 6.50) | |  |  |  | |  | |  | |  |  |  |
| *Stracker, 2019^51^* |  | | *TB+* | *Mean:*  $45.72 |  | |  | |  | |  | *TB+* | *Mean: $33.47* |
|  |  |  | *Xpert-* | *Mean:*  $2.45 |  |  |  |  |  |  |  | *Xpert -* | *Mean: $11.43* |
| *Timire, 2021^53^* |  | | *DS-TB* | Median (IQR):  $4.08 |  | | *DS-TB* | Median (IQR): $  14.69 | *DS-TB* | Median (IQR):  $1.63 |  | | |
|  |  |  | *DR-TB* | Median (IQR):  $4.08 |  |  | *DR-TB* | Median (IQR): $  7.35 | *DR-TB* | Median (IQR): $0 |  |  |  |
|  |  |  | *Total* | Median (IQR):  $4.08 |  |  | *Total* | Median (IQR): $  14.69 | *Total* | Median (IQR): $0 |  |  |  |
| *Ukwaja, 2013 (1)^56^* |  | | Mean (SD):  $7.94 | | Mean (SD): $0.00 | | Mean (SD):  $2.12 | |  | | *Card/User Fees* | Mean (SD):  $3.17 | |
| *Viney, 2019^59^* | *Mean (SD):* $9.32 | |  | |  | |  | |  | |  | | |
| *Viney, 2022* | Extra-pulmonary TB | Median (IQR): $6.00 (5.00 – 10.00) |  | |  | |  | |  | |  | | |
|  | Pulmonary TB | Median (IQR): $6.00 (4.00 – 6.00) |  |  |  |  |  |  |  |  |  |  |  |
|  | Total | Median (IQR): $6.00 (5.00 – 6.00) |  |  |  |  |  |  |  |  |  |  |  |
| *Vo, 2021* | ACF | Mean (95% CI): $7.00 (2.00 – 12.00) |  | |  | |  | |  | |  | | |
|  |  | Median (IQR): $2.00 (0 – 4.00) |  |  |  |  |  |  |  |  |  |  |  |
|  | PCF | Mean (95% CI): $6.00 (3.00 – 9.00) |  | |  | |  | |  | |  | | |
|  |  | Median (IQR): $3.00 (1.00 – 8.00) |  |  |  |  |  |  |  |  |  |  |  |
|  | Total | Mean (95% CI): $7.00 (4.00 – 9.00) |  | |  | |  | |  | |  | | |
|  |  | Median (IQR): $2.00 (1.00 – 6.00) |  |  |  |  |  |  |  |  |  |  |  |
| *Walcott, 2020^60^* | Mean (SD):  $3.60 | | Mean (SD):  $2.00 | |  | | Mean (SD):  $1.20 | |  | | Phone Call | Mean (SD):  $0.10 | |
|  |  |  |  |  |  |  |  |  |  |  |  | Median (IQR): $0 | |
|  | Mean (SD):  $3.60 | | Median (IQR):  $0.80 | |  |  | Median (IQR): $0 | |  |  | Caregiver | Mean (SD):  $0.40 | |
|  |  |  |  |  |  |  |  |  |  |  |  | Median (IQR): $0 | |
| *Abbreviations: TB – Tuberculosis, DS-TB – Drug sensitive TB, MDR-TB – Multi-drug resistant TB, DR-TB – Drug resistant TB, RS-TB – Rifampicin sensitive TB, RMR-TB – Rifampicin mono-resistant TB, HIV – Human Immunodeficiency Virus, LTBI – Latent TB Infection, CHC – Community health centre, ACF – Active case finding, PCF – Passive case finding, SD – Standard deviation, IQR – Interquartile range, CI – Confidence Interval* | | | | | | | | | | | | | |
