# Supplementary material for: The catastrophic cost of TB care: Understanding costs incurred by individuals undergoing TB care in low-, middle-, and high-income settings – A systematic review
Source: PLOS Glob Public Health. 2025 Apr 2;5(4):e0004283. doi: 10.1371/journal.pgph.0004283 (PMC12005564; doi:10.1371/journal.pgph.0004283)
Supplement: S4 Table — (DOCX) [file pgph.0004283.s010.docx]

## ***Table S4 – Breakdown of the indirect costs incurred by patients during the pre-diagnostic phase of TB care***

|  | *Total* | | | | *Loss of Income* | | | | | | | | | | *Time/Productivity Loss* | | *Caregiver/Guardian Costs* | | *Other* | | |
| --- | --- | --- | --- | --- | --- | --- | --- | --- | --- | --- | --- | --- | --- | --- | --- | --- | --- | --- | --- | --- | --- |
| *Assebe, 2020^18^* |  | | | | *Outpatient* | | | | | | | Mean (SD) - $11 (28) | | |  | |  | |  | | |
|  |  |  |  |  |  |  |  |  |  |  |  | Median (IQR) - $0 (0-5) | | |  |  |  |  |  |  |  |
|  |  |  |  |  | *Inpatient* | | | | | | | Mean (SD) - $0 (0) | | |  |  |  |  |  |  |  |
|  |  |  |  |  |  |  |  |  |  |  |  | Median (IQR) - $0 (0) | | |  |  |  |  |  |  |  |
|  |  |  |  |  | *Total* | | | | | | | Mean (SD) - $11 (26) | | |  |  |  |  |  |  |  |
|  |  |  |  |  |  |  |  |  |  |  |  | Median (IQR) - $0 (0-3) | | |  |  |  |  |  |  |  |
| *Chandra, 2021 ^(1) 21^* | *Public* | | | *Median (IQR): $38.5(16.8-140.3)* |  | | | | | | | | | |  | |  | |  | | |
|  | *Private* | | | *Median (IQR): $70.1(16.6-192.4)* |  |  |  |  |  |  |  |  |  |  |  |  |  |  |  |  |  |
|  | *Total* | | | *Median (IQR): $43.6 (16.8-150.8)* |  |  |  |  |  |  |  |  |  |  |  |  |  |  |  |  |  |
| *Chandra, 2021 ^(2) 22^* | *Median (IQR): $0 (0-15)* | | | |  | | | | | | | | | |  | |  | | *Administrative Burden* | | Median (IQR): $0 (0) |
|  | *Mean (SD): $ 32 (76)* | | | |  |  |  |  |  |  |  |  |  |  |  |  |  |  |  |  | Mean (SD): $0 (0) |
| *Chatterjee, 2023* |  | | | | *Consultation* | | | | | | *Mean: $1,805.03* | | | |  | |  | |  | | |
|  |  |  |  |  | *Hospitalization* | | | | | | *Mean: $3,014.45* | | | |  |  |  |  |  |  |  |
| *Collins, 2018^24^* | *MDR-TB* | | | Median: $0 |  | | | | | | | | | |  | |  | |  | | |
| *De Siqueria Filha, 2018^25^* | *TB/HIV* | | | *Mean - $158.50* | *TB/HIV* | | | | | | | *Mean - $116.44* | | | *TB/HIV* | *Mean - $42.06* |  | |  | | |
|  | *LTBI/HIV* | | | *Mean - $2.91* | *LTBI/HIV* | | | | | | | Mean - $0 | | | *LTBI/HIV* | *Mean - $2.91* |  |  |  |  |  |
| *Devoid, 2022* |  | | | | *Mean (SD): $23.84 (143.21)* | | | | | | | | | |  | | *Mean (SD): $0.94 (1.85)* | |  | | |
| *Ellaban, 2021^26^* | *Median (IQR): $0.0 (0.0-75.0)* | | | |  | | | | | | | | | |  | |  | |  | | |
| *Fuady, 2018^29^* | *TB* | | | *Median: $0.34* | *TB* | *Patient* | | | | | | | | *Median: $0* |  | |  | |  | | |
|  | *MDR-TB* | | | *Median: $1.38* |  | *Guardian* | | | | | | | | *Median: $0* |  |  |  |  |  |  |  |
|  | *TB* | | | *Median: $0.34* | *MDR-TB* | *Patient* | | | | | | | | *Median: $0* |  |  |  |  |  |  |  |
|  |  |  |  |  |  | *Guardian* | | | | | | | | *Median: $0* |  |  |  |  |  |  |  |
| *Fuady, 2020^27^* | Mean: $2.06 | | | |  | | | | | | | | | |  | |  | |  | | |
| *Getahun, 2016^30^* | *Mean: $16.67* | | | |  | | | | | | | | | |  | |  | |  | | |
|  | *Median: $13.19* | | | |  |  |  |  |  |  |  |  |  |  |  |  |  |  |  |  |  |
| *Gurung, 2019^33^* | *ACF* | | | *Median: $68.92* | *ACF* | | | | *Median: $55.78* | | | | | | *ACF* | *Median: $4.78* |  | |  | | |
|  | *PCF* | | | *Median: $50.00* | *PCF* | | | | *Median: $33.32* | | | | | | *PCF* | *Median: $14.54* |  |  |  |  |  |
|  | *Total* | | | *Median: $55.46* | *Total* | | | | *Median: $44.06* | | | | | | *Total* | *Median: $8.47* |  |  |  |  |  |
| *Gurung, 2021^32^* | *ACF* | | | *Mean (95% CI) - $7.5 (5.6–9.5)* |  | | | | | | | | | |  | |  | |  | | |
|  |  |  |  | *Median (IQR) - $4.3 (1.9–8.7)* |  |  |  |  |  |  |  |  |  |  |  |  |  |  |  |  |  |
|  | *PCF* | | | *Mean (95% CI) - $15.3 (11.9–18.6)* |  |  |  |  |  |  |  |  |  |  |  |  |  |  |  |  |  |
|  |  |  |  | *Median (IQR) - $10.0 (5.6–18.0)* |  |  |  |  |  |  |  |  |  |  |  |  |  |  |  |  |  |
|  | *Total* | | | *Mean (95% CI) - $11.5 (9.4–13.5)* |  |  |  |  |  |  |  |  |  |  |  |  |  |  |  |  |  |
|  |  |  |  | *Median (IQR) - $6.7 (3.3–13.6)* |  |  |  |  |  |  |  |  |  |  |  |  |  |  |  |  |  |
| *Kaswa, 2021* |  | | | | DS-TB | | | | | *Mean (95% CI): $3.20 (1.70 – 5.20)* | | | | |  | |  |  |  | | |
|  |  |  |  |  | DR-TB | | | | | *Mean (95% CI): $4.00 (2.00 – 6.00)* | | | | |  |  |  |  |  |  |  |
|  |  |  |  |  | Total | | | | | *Mean (95% CI): $3.60 (1.80 – 5.30)* | | | | |  |  |  |  |  |  |  |
| *Kilale, 2022* | *Mean (SD): $8.90 (44.60)* | | | |  | | | |  | | | | | |  | |  |  |  | | |
|  | *Median (IQR): $3.10 (1.60 – 5.60)* | | | |  |  |  |  |  |  |  |  |  |  |  |  |  |  |  |  |  |
| *Lu, 2020^35^* |  | | | | *Residents* | | | | *Mean: $610.64* | | | | | |  | | *Residents* | *Mean: $23.68* |  | | |
|  |  |  |  |  | *Migrants* | | | | *Mean: $310.27* | | | | | |  |  | *Migrants* | *Mean: $14.69* |  |  |  |
| *Mauch, 2013^(1) 36^* | *Ghana* | | | *Mean; $55.78* | *Ghana* | | | | *Mean; $55.78* | | | | | |  | |  | |  | | |
|  |  |  |  | *Median (IQR): $24.89 (6.30 – 49.78)* |  |  |  |  | *Median (IQR): $24.89 (6.30 – 49.78)* | | | | | |  |  |  |  |  |  |  |
|  | *Vietnam* | | | *Mean: $186.30* | *Vietnam* | | | | *Mean: $186.30* | | | | | |  |  |  |  |  |  |  |
|  |  |  |  | Median (IQR): $161.84 (107.29 – 230.97) |  |  |  |  | Median (IQR): $161.84 (107.29 – 230.97) | | | | | |  |  |  |  |  |  |  |
|  | *Dominican Republic* | | | *Mean: $211.12* | *Dominican Republic* | | | | *Mean: $211.12* | | | | | |  |  |  |  |  |  |  |
|  |  |  |  | *Median (IQR): $133.78 (55.24 – 238.24)* |  |  |  |  | *Median (IQR): $133.78 (55.24 – 238.24)* | | | | | |  |  |  |  |  |  |  |
| *Mauch, 2013 ^(2) 38^* | New | | | *Median: $654.98* | New | | | | *Median: $654.98* | | | | | |  | |  | |  | | |
|  | Retreatment | | | *Median: $199.18* | Retreatment | | | | *Median: $199.18* | | | | | |  |  |  |  |  |  |  |
|  | MDR-TB | | | *Median: $2836.59* | MDR-TB | | | | *Median: $2836.59* | | | | | |  |  |  |  |  |  |  |
| *Mauch, 2011^37^* | *Median: $69.16* | | | |  | | | |  | | | | | |  | |  | |  | | |
| *Morishita, 2016^40^* | *ACF* | | | Mean (SD): $  9.48 | *Sick Leave* | | *ACF* | | | | | | Mean (SD): $  8.67 | |  | | *ACF* | Mean (SD): $  0.56 |  | | |
|  |  |  |  |  |  |  |  |  |  |  |  |  | Median (IQR): $  0.00 | |  |  |  |  |  |  |  |
|  |  |  |  | Median (IQR): $  0.00 |  |  | *PCF* | | | | | | Mean (SD): $  19.63 | |  |  |  | Median (IQR): $  0.20 |  |  |  |
|  |  |  |  |  |  |  |  |  |  |  |  |  | Median (IQR): $  0.00 | |  |  |  |  |  |  |  |
|  | *PCF* | | | Mean (SD): $  30.13 | *Care Seeking* | | *ACF* | | | | | | Mean (SD): $  0.82 | |  |  | *PCF* | Mean (SD): $3.26 |  |  |  |
|  |  |  |  |  |  |  |  |  |  |  |  |  | Median (IQR): $  0.00 | |  |  |  |  |  |  |  |
|  |  |  |  | Median (IQR): $  0.71 |  |  | *PCF* | | | | | | Mean (SD): $  10.50 | |  |  |  | Median (IQR): $  0.56 |  |  |  |
|  |  |  |  |  |  |  |  |  |  |  |  |  | Median (IQR): $  0.00 | |  |  |  |  |  |  |  |
| *Muniyandi, 2020^42^* | *Mean (SD): $153.61 (304.00)* | | | |  | | | |  | | | | | |  | |  | |  | | |
|  | *Median (IQR): $0.00 (0.00 – 1709.74)* | | | |  |  |  |  |  |  |  |  |  |  |  |  |  |  |  |  |  |
| *Muttamba, 2020^43^* |  | | | | DS-TB | | | | Mean (95% CI): $  0.68 | | | | | |  | |  | |  | | |
|  |  |  |  |  | MDR-TB | | | | Mean (95% CI): $  0.61 | | | | | |  |  |  |  |  |  |  |
|  |  |  |  |  | Total | | | | Mean (95% CI): $  0.68 | | | | | |  |  |  |  |  |  |  |
| *Pedrazzoli, 2021^46^* | *Insured* | | | *Mean: $*  1.43 |  | | | | | | | | | |  | |  | |  | | |
|  |  |  |  | *Median (IQR): $*  0.49 |  |  |  |  |  |  |  |  |  |  |  |  |  |  |  |  |  |
|  | *Uninsured* | | | *Mean: $*  3.34 |  |  |  |  |  |  |  |  |  |  |  |  |  |  |  |  |  |
|  |  |  |  | *Median (IQR): $*  0.67 |  |  |  |  |  |  |  |  |  |  |  |  |  |  |  |  |  |
| *Pham, 2023* |  | | | | *MDR-TB* | | | Median (IQR): $32.80 (20.80 – 83.97) | | | | | | |  | |  | | *Ambulatory care visits* | Median (IQR): $25.08 (13.00 – 54.10) | |
| *Ramma, 2015^48^* | *Inpatient* | | | Mean (SD): $  114.79 | *Seeking Care* | | *Inpatient* | | | | | | *Mean (SD): $*  5.15 | |  | | *Inpatient*  *Outpatient* | *Mean (SD): $*  3.17  *Median (IQR): $*  0.99 |  | | |
|  |  |  |  |  |  |  |  |  |  |  |  |  | *Median (IQR): $*  0.43 | |  |  |  |  |  |  |  |
|  |  |  |  |  |  |  | *Outpatient* | | | | | | *Mean (SD): $*  3.92 | |  |  |  | *Mean (SD): $*  13.33  *Median (IQR): $*  0.00 |  |  |  |
|  |  |  |  |  |  |  |  |  |  |  |  |  | *Median (IQR): $*  1.37 | |  |  |  |  |  |  |  |
|  | *Outpatient* | | | *Mean (SD): $*  29.87 | *Hospitalization* | | *Inpatient* | | | | | | *Mean (SD): $*  106.42 | |  |  | *Inpatient*  *Outpatient* | *Mean (SD): $*  3.17  *Median (IQR): $*  0.99 |  |  |  |
|  |  |  |  |  |  |  |  |  |  |  |  |  | *Median (IQR): $*  70.41 | |  |  |  |  |  |  |  |
|  |  |  |  |  |  |  | *Outpatient* | | | | | | *Mean (SD): $*  12.62 | |  |  |  | *Mean (SD): $*  13.33 |  |  |  |
|  |  |  |  |  |  |  |  |  |  |  |  |  | *Median (IQR): $*  3.17 | |  |  |  |  |  |  |  |
| *Ukwaja, 2013 (1)^56^* | Mean (SD): $271.46 | | | | Mean (SD): $270.40 | | | | | | | | | | *Value of time* | *Mean (SD):*  $1.06 |  | |  | | |
| *Van der Hof, 2016^58^* | Ethiopia | DS-TB | | Median (IQR):  $0.00 |  | | | | | | | | | |  | |  | |  | | |
|  |  | MDR-TB | | Median (IQR):  $0.00 |  |  |  |  |  |  |  |  |  |  |  |  |  |  |  |  |  |
|  | *Indonesia* | DS-TB | | Median (IQR):  $1.29 |  |  |  |  |  |  |  |  |  |  |  |  |  |  |  |  |  |
|  |  | MDR-TB | | Median (IQR):  $0.97 |  |  |  |  |  |  |  |  |  |  |  |  |  |  |  |  |  |
|  | *Kazakhstan* | DS-TB | | Median (IQR):  $1.07 |  |  |  |  |  |  |  |  |  |  |  |  |  |  |  |  |  |
|  |  | MDR-TB | | - |  |  |  |  |  |  |  |  |  |  |  |  |  |  |  |  |  |
| *Vo, 2021* | ACF | | Mean (95% CI): $5.00 (0 – 10.00) | |  | | | | | | | | | |  |  |  | |  | | |
|  |  |  | Median (IQR): $1.00 (0.50 – 1.00) | |  |  |  |  |  |  |  |  |  |  |  |  |  |  |  |  |  |
|  | PCF | | Mean (95% CI): $20.00 (3.00 -36.00) | |  |  |  |  |  |  |  |  |  |  |  |  |  |  |  |  |  |
|  |  |  | Median (IQR): $2.00 (1.00 -5.00) | |  |  |  |  |  |  |  |  |  |  |  |  |  |  |  |  |  |
|  | Total | | Mean (95% CI): $12.00 (4.00 – 20.00) | |  |  |  |  |  |  |  |  |  |  |  |  |  |  |  |  |  |
|  |  |  | Median (IQR): $1.00 (1.00 – 3.00) | |  |  |  |  |  |  |  |  |  |  |  |  |  |  |  |  |  |
| *Walcott, 2020^60^* |  | | | |  | | | | | | | | | | *Patients (n=49)* | Mean (SD):  $15.62 |  | |  | | |
|  |  |  |  |  |  |  |  |  |  |  |  |  |  |  |  | *Median (IQR):* $6.81 |  |  |  |  |  |
|  |  |  |  |  |  |  |  |  |  |  |  |  |  |  | *All* | Mean (SD):  $7.61 |  |  |  |  |  |
|  |  |  |  |  |  |  |  |  |  |  |  |  |  |  |  | Median (IQR): $0 |  |  |  |  |  |
| *Abbreviations: TB – Tuberculosis, DS-TB – Drug sensitive TB, MDR-TB – Multi-drug resistant TB, DR-TB – Drug resistant TB, RS-TB – Rifampicin sensitive TB, RMR-TB – Rifampicin mono-resistant TB, HIV – Human Immunodeficiency Virus, LTBI – Latent TB Infection, ACF – Active case finding, PCF – Passive case finding, SD – Standard deviation, IQR – Interquartile range, CI – Confidence Interval* | | | | | | | | | | | | | | | | | | | | | |
