# Supplementary material for: The catastrophic cost of TB care: Understanding costs incurred by individuals undergoing TB care in low-, middle-, and high-income settings – A systematic review
Source: PLOS Glob Public Health. 2025 Apr 2;5(4):e0004283. doi: 10.1371/journal.pgph.0004283 (PMC12005564; doi:10.1371/journal.pgph.0004283)
Supplement: S5 Table — (DOCX) [file pgph.0004283.s011.docx]

## ***Table S5 – Breakdown of the direct medical costs incurred by patients during the post-diagnostic phase of TB care***

|  | *Total* | | | | *Consultation* | | | *Medication* | | | | | | | *Diagnostic Imaging* | | *Follow-up Tests* | | | | *Hospitalization* | | | | | *Other* | | | | | |
| --- | --- | --- | --- | --- | --- | --- | --- | --- | --- | --- | --- | --- | --- | --- | --- | --- | --- | --- | --- | --- | --- | --- | --- | --- | --- | --- | --- | --- | --- | --- | --- |
| *Aia, 2022* | *DS-TB* | | *Mean (95% CI): $26.10 (11.80 – 40.40)* | |  | | |  | | | | | | |  | |  | | | |  | | | | |  | | | | | |
|  | *MDR-TB* | | *Mean (95% CI): $3.00 (2.70 – 8.70)* | |  |  |  |  |  |  |  |  |  |  |  |  |  |  |  |  |  |  |  |  |  |  |  |  |  |  |  |
|  | *Total* | | *Mean (95% CI): $25.60 (11.70 – 39.60)* | |  |  |  |  |  |  |  |  |  |  |  |  |  |  |  |  |  |  |  |  |  |  |  |  |  |  |  |
| *Aung, 2021^19^* | *MDR-TB* | *Median (min-max): $199.19* | | |  | | |  | | | | | | |  | |  | | | |  | | | | |  | | | | | |
|  | *DS-TB* | *Median (min-max): $72.89* | | |  |  |  |  |  |  |  |  |  |  |  |  |  |  |  |  |  |  |  |  |  |  |  |  |  |  |  |
|  | *Total* | *Median (min-max): $72.89* | | |  |  |  |  |  |  |  |  |  |  |  |  |  |  |  |  |  |  |  |  |  |  |  |  |  |  |  |
| *Chandra, 2021 ^(2) 22^* | *Median (IQR): $0* | | | |  | | | *Median (IQR): $0* | | | | | | | *Median (IQR): $0* | | *Median (IQR): $0* | | | | *Median (IQR): $0* | | | | |  | | | | | |
|  | *Mean (SD): $1.70 (10.60)* | | | |  | | | *Mean (SD): $1.20 (10.40)* | | | | | | | *Mean (SD): $0 (0)* | | *Mean (SD): $0.50 (2.30)* | | | | *Mean (SD): $0 (0)* | | | | |  |  |  |  |  |  |
| *Chatterjee, 2023* |  | | | | *Intensive Phase* | | *Mean: $117.28* | *Intensive Phase* | | | | | *Mean: $328.41* | |  | |  | | |  | *Intensive Phase* | | | *Mean: $0.00* | |  |  | | |  | |
|  |  |  |  |  | *Continuation Phase* | | *Mean: $1,042.18* | *Continuation Phase* | | | | | *Mean: $645.29* | |  |  |  |  |  |  | *Continuation Phase* | | | *Mean: $1,557.33* | |  |  |  |  |  |  |
|  |  |  |  |  | *Post-Treatment Phase* | | *Mean: $811.49* | *Post-Treatment Phase* | | | | | *Mean: $59.53* | |  |  |  |  |  |  | *Post-Treatment Phase* | | | *Mean: $9.12* | |  |  |  |  |  |  |
| *Chittamany, 2020 ^23^* | *DS-TB* | *Median (IQR): $42.95* | | |  | | | *DS-TB* | | | | | *Median (IQR): $0 (0 – 49.31)* | |  | | *DS-TB* | | | *Median (IQR): $0 (0 – 4.77)* | *DS-TB* | | | | *Median (IQR): $20.68 (20.68 – 47.72)* | *DOT* | *DS-TB* | | | *Median (IQR): $0 (0-0)* | |
|  | *DR-TB* | *Median (IQR): $334.06* | | |  |  |  | *DR-TB* | | | | | *Median (IQR): $209.00 (109.79 – 3102.01)* | |  |  | *DR-TB* | | | *Median (IQR): $0 (0 – 3.18)* | *MDR-TB* | | | | *Median (IQR): $330.88* |  | *DR-TB* | | | *Median (IQR): $0 (0-0)* | |
|  | *Total* | *Median (IQR): $46.13* | | |  |  |  | *Total* | | | | | *Median (IQR): $0 (0 – 49.31)* | |  |  | *Total* | | | *Median (IQR): $0 (0 – 4.77)* | *Total* | | | | *Median (IQR): $20.68 (20.68 – 47.72)* |  | *Total* | | | *Median (IQR): $0 (0-0)* | |
| *Collins, 2018*^24^* |  | | | |  | | |  | | | | | | |  | | *Addis Ababa (MDR)* | | | *Median (IQM): $228.73* | *Addis Ababa (MDR)* | | | | *Median (IQM): $1.25* |  | | | | | |
|  |  |  |  |  |  |  |  |  |  |  |  |  |  |  |  |  | *Gonder (MDR)* | | | *Median (IQM): $146.23* | *Gonder (MDR)* | | | | *Median (IQM): $11.25* |  |  |  |  |  |  |
| *De Siqeruia Filha, 2018^25^* | *TB/HIV* | *Mean: $32.80* | | | *TB/HIV* | *Mean: $24.90* | | *TB/HIV* | | | | | *Mean: $6.48* | |  | | *TB/HIV* | | | *Mean: $1.42* |  | | | | |  | | | | | |
|  | *LTBI/HIV* | *Mean: $22.05* | | | *LTBI/HIV* | *Mean: $0.17* | | *LTBI/HIV* | | | | | *Mean: $9.57* | |  |  | *LTBI/HIV* | | | *Mean: $12.31* |  |  |  |  |  |  |  |  |  |  |  |
| *Devoid, 2022* | *Intensive Phase* | | *Mean: $10.26* | | *Intensive Phase* | | | | *Mean (SD): 0.01 (0.03)* | | | | | |  | |  | | |  | *Intensive Phase* | | | *Mean (SD): 0.01 (0.08)* | |  | | | | | |
|  | *Continuation Phase* | | *Mean: $17.13* | | *Continuation Phase* | | | | *-* | | | | | |  |  |  | | |  | *Continuation Phase* | | | *Mean (SD): 0.09 (1.43)* | |  |  |  |  |  |  |
| *Diallo, 2022* | DS-TB | | Mean (95% CI): $84.3 (37 - 131) | |  | | |  | | | | | | |  | |  | | | |  | | | | |  | | | | | |
|  | DR-TB | | Mean (95% CI): $103.3 (–52 - –259) | |  |  |  |  |  |  |  |  |  |  |  |  |  |  |  |  |  |  |  |  |  |  |  |  |  |  |  |
|  | Total | | Mean (95% CI): $84.6 (39 - 130) | |  |  |  |  |  |  |  |  |  |  |  |  |  |  |  |  |  |  |  |  |  |  |  |  |  |  |  |
| *Ellaban, 2021^26^* | *First two months of treatment (intensive phase)* | *Median (IQR): $0.00 (0.00 – 0.00)* | | |  | | |  | | | | | | |  | |  | | | |  | | | | |  | | | | | |
|  | *Second two months of treatment* | *Median (IQR): $0.00 (0.00 – 0.00)* | | |  |  |  |  |  |  |  |  |  |  |  |  |  |  |  |  |  |  |  |  |  |  |  |  |  |  |  |
|  | *Third two months of treatment* | *Median (IQR): $0.00 (0.00 – 0.00)* | | |  |  |  |  |  |  |  |  |  |  |  |  |  |  |  |  |  |  |  |  |  |  |  |  |  |  |  |
| *Florentino, 2022* |  |  | | |  | | | *Urban DS-TB* | | | | | *Mean (SD): $0.70 (20.50)* | |  | | *Urban DS-TB* | | | *Mean (SD): $45.80 (156.30)* | *Urban DS-TB* | | | *Mean (SD): $8.70 (90.60)* | |  |  | | |  | |
|  |  |  |  |  |  |  |  | *Rural DS-TB* | | | | | *Mean (SD): $0 (0)* | |  |  | *Rural DS-TB* | | | *Mean (SD): $74.40 (271.70)* | *Rural DS-TB* | | | *Mean (SD): $14.70 (112.20)* | |  |  |  |  |  |  |
|  |  |  |  |  |  |  |  | *DR-TB* | | | | | *Mean (SD): $0 (0)* | |  |  | *DR-TB* | | | *Mean (SD): $135.80 (913.70)* | *DR-TB* | | | *Mean (SD): $62.60 (730.00)* | |  |  |  |  |  |  |
|  |  |  |  |  |  |  |  | *Total* | | | | | *Mean (SD): $0.10 (5.20)* | |  |  | *Total* | | | *Mean (SD): $70.70 (353.50)* | *Total* | | | *Mean (SD): $14.50 (147.00)* | |  |  |  |  |  |  |
| *Fuady, 2018^29^* | *DS-TB* | *Median (IQR): $0.00* | | | *DS-TB* | *Median (IQR): $0.00* | |  | | | | | | |  | | *DS-TB* | | | *Median (IQR): $0.00 (0-0)* | *DS-TB* | | *Median (IQR): $0.00 (0-0)* | | | *Adverse events* | *DS-TB* | | | *Median (IQR): $0.00 (0-0)* | |
|  | *MDR-TB* | *Median (IQR): $5.16* | | | *MDR-TB* | *Median (IQR): $0.00 (0-0)* | |  |  |  |  |  |  |  |  |  | *MDR-TB* | | | *Median (IQR): $0.00 (0.00)* | *MDR-TB* | | *Median (IQR): $2.75* | | |  | *MDR-TB* | | | *Median (IQR): $0.00 (0-0)* | |
| *Getahun, 2016^30^* |  | | | | *Mean (SD): $27.28* | | |  | | | | | | |  | | *Unscheduled Additional Follow-up* | | | *Mean (SD): $9.73* | *Mean (SD): $76.94* | | | | |  | | | | | |
|  |  |  |  |  | *Median (R): $26.76* | | |  |  |  |  |  |  |  |  |  |  |  |  | *Median (R): $6.64* | *Median (R): $51.56* | | | | |  |  |  |  |  |  |
| *Gospodarevskaya, 2014^† 31^* |  | | | |  | | | *Tanzania* | | *First two months of treatment* | | | *Mean: $3.28* | |  | | *Tanzania* | *First two months of treatment* | | *-* | *Tanzania* | *First two months of treatment* | | | *Mean: $5.28* |  | | | | | |
|  |  |  |  |  |  |  |  |  |  | *Most recent two months of treatment* | | | *Mean: $1.90* | |  |  |  | *Most recent two months of treatment* | | *Mean: $0.29* |  | *Most recent two months of treatment* | | | *Mean: $0.00* |  |  |  |  |  |  |
|  |  |  |  |  |  |  |  |  |  | *Total treatment* | | | *Mean: $7.07* | |  |  |  | *Total treatment* | | *Mean: $0.58* |  | *Total treatment* | | | *Mean: $5.28* |  |  |  |  |  |  |
|  |  |  |  |  |  |  |  | *Bangladesh* | | *First two months of treatment* | | | *Mean: $3.06* | |  |  | *Bangladesh* | *First two months of treatment* | | *-* | *Bangladesh* | *First two months of treatment* | | | *Mean: $18.88* |  |  |  |  |  |  |
|  |  |  |  |  |  |  |  |  |  | *Most recent two months of treatment* | | | *Mean: $2.16* | |  |  |  | *Most recent two months of treatment* | | *Mean: $2.40* |  | *Most recent two months of treatment* | | | *Mean: $0.00* |  |  |  |  |  |  |
|  |  |  |  |  |  |  |  |  |  | *Total treatment* | | | *Mean: $7.37* | |  |  |  | *Total treatment* | | *Mean: $4.80* |  | *Total treatment* | | | *Mean: $18.88* |  |  |  |  |  |  |
| *Gurung, 2019 ^33^* | *ACF* |  | | |  | | | *ACF* | | | *-* | | | |  | |  | | | |  | | | | |  | | | | | |
|  | *PCF* | *Median (IQR): $0.00* | | |  | | | *PCF* | | | *Median (IQR): $0.00* | | | |  |  |  |  |  |  |  |  |  |  |  |  |  |  |  |  |  |
|  | *Total* | *-* | | |  | | | *Total* | | | *-* | | | |  |  |  |  |  |  |  |  |  |  |  |  |  |  |  |  |  |
| *Gurung, 2021^32^* | *ACF* | *Mean (95% CI): $19.50 (13.50 – 25.50)* | | |  | | |  | | | | | | |  | |  | | | |  | | | | |  | | | | | |
|  |  | *Median (IQR): $10.80 (6.30 – 20.70)* | | |  |  |  |  |  |  |  |  |  |  |  |  |  |  |  |  |  |  |  |  |  |  |  |  |  |  |  |
|  | *PCF* | *Mean (95% CI): $21.10 (14.90 – 27.40)* | | |  |  |  |  |  |  |  |  |  |  |  |  |  |  |  |  |  |  |  |  |  |  |  |  |  |  |  |
|  |  | *Median (IQR): $ 12.20 (7.20 – 21.20)* | | |  |  |  |  |  |  |  |  |  |  |  |  |  |  |  |  |  |  |  |  |  |  |  |  |  |  |  |
|  | *Total* | *Mean 95% CI): $20.30 (16.00 – 24.60)* | | |  |  |  |  |  |  |  |  |  |  |  |  |  |  |  |  |  |  |  |  |  |  |  |  |  |  |  |
|  |  | *Median (IQR): $11.80 (6.60 – 20.70)* | | |  |  |  |  |  |  |  |  |  |  |  |  |  |  |  |  |  |  |  |  |  |  |  |  |  |  |  |
| *Kaswa, 2022* | DS-TB | | *Mean (95% CI): $52.00 (23.10 – 80.10)* | |  | | |  | | | | | | |  | |  | | | |  | | | | |  | | | | | |
|  | DR-TB | | *Mean (95% CI): $92.00 (29.70 – 153.70)* | |  |  |  |  |  |  |  |  |  |  |  |  |  |  |  |  |  |  |  |  |  |  |  |  |  |  |  |
|  | Total | | *Mean (95% CI): 59.00 (31.00 – 86.70)* | |  |  |  |  |  |  |  |  |  |  |  |  |  |  |  |  |  |  |  |  |  |  |  |  |  |  |  |
| *Kilale, 2022* | *Mean (SD): $29.30 (144.40)* | | | |  | | |  | | | | | | |  | |  | | | |  | | | | |  | | | | | |
|  | *Median (IQR): 0.00 (0.00 – 4.20)* | | | |  |  |  |  |  |  |  |  |  |  |  |  |  |  |  |  |  |  |  |  |  |  |  |  |  |  |  |
| *Kirubi, 2021^34^* | *Median (IQR): $10.86* | | | |  | | |  | | | | | | |  | |  | | | |  | | | | |  | | | | | |
| *Loureiro, 2024* | *Mean: $4.42* | | | |  | | |  | | | | | | |  | | *Mean: $2.31* | | | | *Mean: $2.11* | | | | |  | | | | | |
| *Lu, 2020^35^* | *Residents* | *Mean: $3,321.78* | | |  | | | *Residents* | | | *Mean: $2,185.94* | | | | *Residents* | *Mean: $867.54* |  | | | |  | | | | | *Medical Supplies* | | *Residents* | | *Mean: $268.30* | |
|  | *Migrants* | *Mean: $1,938.93* | | |  |  |  | *Migrants* | | | *Mean: $1,321.70* | | | | *Migrants* | *Mean: $540.79* |  |  |  |  |  |  |  |  |  |  |  | *Migrants* | | *Mean: $36.90* | |
| *Mauch, 2013^(1) 36^* |  | | | |  | | | *Ghana* | | | *Mean: $7.29* | | | |  | | *Ghana* | | | *Mean: $0.15* | *Ghana* | | | | *Mean: $6.15* | *DOTS* | | *Ghana* | | *Mean: $3.95* | |
|  |  |  |  |  |  |  |  |  |  |  | *Median (IQR): $0.29* | | | |  |  |  |  |  | *Median (IQR): $0.00* |  |  |  |  | *Median (IQR): $2.34* |  |  |  |  | *Median (IQR): $0.00* | |
|  |  |  |  |  |  |  |  | *Vietnam* | | | *Mean: $0.74* | | | |  |  | *Vietnam* | | | *Mean: $1.12* | *Vietnam* | | | | *Mean: $26.49* |  |  | *Vietnam* | | *Mean: $4.04* | |
|  |  |  |  |  |  |  |  |  |  |  | *Median (IQR): $0.13* | | | |  |  |  |  |  | *Median (IQR): $0.67* |  |  |  |  | *Median (IQR): $9.88* |  |  |  |  | *Median (IQR): $1.80* | |
|  |  |  |  |  |  |  |  | *Dominican Republic* | | | *Mean: $3.52* | | | |  |  | *Dominican Republic* | | | *Mean: $3.62* | *Dominican Republic* | | | | *Mean: $18.88* |  |  | *Dominican Republic* | | *Mean: $1.00* | |
|  |  |  |  |  |  |  |  |  |  |  | *Median (IQR): $0.80* | | | |  |  |  |  |  | *Median (IQR): $1.61* |  |  |  |  | *Median (IQR): $0.00* |  |  |  |  | *Median (IQR): $0.80* | |
| *Mauch, 2013^(2) 38^* |  | | | |  | | | *New* | | | *Median: $5.59* | | | |  | | *New* | | | *Median: $14.97* | *New* | | | | *Median: $55.00* | *DOTS* | | *New* | | *Median: $20.92* | |
|  |  |  |  |  |  |  |  | *Retreatment* | | | *Median: $11.27* | | | |  |  | *Retreatment* | | | *Median: $29.48* | *Retreatment* | | | | *Median: $13.34* |  |  | *Retreatment* | | *Median: $25.79* | |
|  |  |  |  |  |  |  |  | *MDR* | | | *Median: $12.53* | | | |  |  | *MDR* | | | *Median: $9.38* | *MDR* | | | | *Median: $50.04* |  |  | *MDR* | | *Median: $52.48* | |
| *Mauch, 2011^37^* |  | | | |  | | |  | | | | | | |  | |  | | | |  | | | | | *DOTS* | | | | *Median: $0.00* | |
| *McAllister, 2020^39^* |  | | | | *CHC* | *Median (IQR): $1.83* | | *CHC* | | | | *-* | | | *CHC* | *Median (IQR): $13.85* | *CHC* | | *Median (IQR): $4.05* | | *CHC* | | | | *Median (IQR): $32.94* |  | | | | | |
|  |  |  |  |  | *Public Hospital* | *Median (IQR): $7.60* | | *Public Hospital* | | | | *Median (IQR): $4.61* | | | *Public Hospital* | *Median (IQR): $8.61* | *Public Hospital* | | *Median (IQR): $5.83* | | *Public Hospital* | | | | *Median (IQR): $50.66* |  |  |  |  |  |  |
|  |  |  |  |  | *Private Hospital* | *Median (IQR): $9.12* | | *Private Hospital* | | | | *Median (IQR): $15.20* | | | *Private Hospital* | *Median (IQR): $5.83* | *Private Hospital* | | *Median (IQR): $6.70* | | *Private Hospital* | | | | *Median (IQR): $278.66* |  |  |  |  |  |  |
|  |  |  |  |  | *Private Practice* | *Median (IQR): $17.10* | | *Private Practice* | | | | *Median (IQR): $48.14* | | | *Private Practice* | *Median (IQR): $10.13* | *Private Practice* | | *Median (IQR): $14.19* | | *Private Practice* | | | | *-* |  |  |  |  |  |  |
| *Morishita, 2016^40^* |  | | | |  | | |  | | | | | | |  | |  | | | | *ACF* | | | | *Mean (SD): $0.00* |  | | | | | |
|  |  |  |  |  |  |  |  |  |  |  |  |  |  |  |  |  |  |  |  |  |  |  |  |  | *Median (IQR): $0.00* |  |  |  |  |  |  |
|  |  |  |  |  |  |  |  |  |  |  |  |  |  |  |  |  |  |  |  |  | *PCF* | | | | *Mean (SD): $1.38* |  |  |  |  |  |  |
|  |  |  |  |  |  |  |  |  |  |  |  |  |  |  |  |  |  |  |  |  |  |  |  |  | *Median (IQR): $0.00* |  |  |  |  |  |  |
| *Mudzengi, 2017^41^* | *Study Clinic* | *TB/HIV* | | *Mean (SD): $0.00 (0.00)* |  | | |  | | | | | | |  | |  | | | |  | | | | |  | | | | | |
|  |  | *TB* | | *Mean (SD): $0.00 (0.00)* |  |  |  |  |  |  |  |  |  |  |  |  |  |  |  |  |  |  |  |  |  |  |  |  |  |  |  |
|  |  | *HIV* | | *Mean (SD): $0.00 (0.00)* |  |  |  |  |  |  |  |  |  |  |  |  |  |  |  |  |  |  |  |  |  |  |  |  |  |  |  |
|  | *Other Facilities* | *TB/HIV* | | *Mean (SD): $*  0.82 |  |  |  |  |  |  |  |  |  |  |  |  |  |  |  |  |  |  |  |  |  |  |  |  |  |  |  |
|  |  | *TB* | | *Mean (SD): $0.03* |  |  |  |  |  |  |  |  |  |  |  |  |  |  |  |  |  |  |  |  |  |  |  |  |  |  |  |
|  |  | *HIV* | | *Mean (SD): $0.42* |  |  |  |  |  |  |  |  |  |  |  |  |  |  |  |  |  |  |  |  |  |  |  |  |  |  |  |
| *Muniyandi, 2020^42^* |  | | | |  | | |  | | | | | | |  | | *Mean (SD): $0.06 (0.50)* | | | | *Hospitalization* | | | | *Mean (SD): $34.90 (146.53)* |  | | | | | |
|  |  |  |  |  |  |  |  |  |  |  |  |  |  |  |  |  |  |  |  |  |  |  |  |  | *Median (IQR): $0.00 (0.00 – 1824.35)* |  |  |  |  |  |  |
|  |  |  |  |  |  |  |  |  |  |  |  |  |  |  |  |  | *Median (Range): $0.00 (0.00 – 7.22)* | | | | *Escort* | | | | *Mean (SD): $3.81 (15.36)* |  |  |  |  |  |  |
|  |  |  |  |  |  |  |  |  |  |  |  |  |  |  |  |  |  |  |  |  |  |  |  |  | *Median (IQR): $0.00 (0.00 – 140.33)* |  |  |  |  |  |  |
|  |  |  |  |  |  |  |  |  |  |  |  |  |  |  |  |  |  |  |  |  | *Visitors* | | | | *Mean (SD): $0.56 (3.89)* |  |  |  |  |  |  |
|  |  |  |  |  |  |  |  |  |  |  |  |  |  |  |  |  |  |  |  |  |  |  |  |  | *Median (IQR): $0.00 (0.00 – 60.14)* |  |  |  |  |  |  |
| *Muttamba, 2020^43^* | *MDR-TB* | *Mean (95% CI): $31.51 (5.01 – 58.06)* | | |  | | |  | | | | | | |  | |  | | | |  | | | | |  | | | | | |
|  | *DS-TB* | *Mean (95% CI): $6.49 (3.68 – 9.29)* | | |  |  |  |  |  |  |  |  |  |  |  |  |  |  |  |  |  |  |  |  |  |  |  |  |  |  |  |
|  | *Total* | *Mean (95% CI): $7.41 (4.48 – 10.37)* | | |  |  |  |  |  |  |  |  |  |  |  |  |  |  |  |  |  |  |  |  |  |  |  |  |  |  |  |
| *Nhung, 2018^44^* | *MDR-TB* | *Mean (95% CI): $2,793.83* | | |  | | |  | | | | | | |  | |  | | | |  | | | | |  | | | | | |
|  | *DS-TB* | *Mean (95% CI): $412.92* | | |  |  |  |  |  |  |  |  |  |  |  |  |  |  |  |  |  |  |  |  |  |  |  |  |  |  |  |
|  | *Total* | *Mean (95% CI): $601.81* | | |  |  |  |  |  |  |  |  |  |  |  |  |  |  |  |  |  |  |  |  |  |  |  |  |  |  |  |
| *Pedrazzoli, 2018^45^* | *MDR-TB* | *Median (IQR): $12.37* | | |  | | |  | | | | | | |  | |  | | | |  | | | | |  | | | | | |
|  | *DS-TB* | *Median (IQR); $22.49* | | |  |  |  |  |  |  |  |  |  |  |  |  |  |  |  |  |  |  |  |  |  |  |  |  |  |  |  |
|  | *Total* | *Median (IQR): $21.27* | | |  |  |  |  |  |  |  |  |  |  |  |  |  |  |  |  |  |  |  |  |  |  |  |  |  |  |  |
| *Pedrazzoli, 2021^46^* | *Uninsured* | *Mean (SD): $40.42* | | |  | | |  | | | | | | |  | |  | | | |  | | | | |  | | | | | |
|  |  | *Median (IQR): $25.22* | | |  |  |  |  |  |  |  |  |  |  |  |  |  |  |  |  |  |  |  |  |  |  |  |  |  |  |  |
|  | *Insured* | *Mean (SD): $43.15* | | |  |  |  |  |  |  |  |  |  |  |  |  |  |  |  |  |  |  |  |  |  |  |  |  |  |  |  |
|  |  | *Median (IQR): $25.22* | | |  |  |  |  |  |  |  |  |  |  |  |  |  |  |  |  |  |  |  |  |  |  |  |  |  |  |  |
| *Prasanna, 2018^47^* |  | | | | *Study population* | *Median (IQR): $0.00* | | *Study population* | | | | | *Median (IQR): $0.00* | | *Study population* | *Median (IQR): $0.00* |  | | | | *Study population* | | | | *Median (IQR): $0.00* |  | | | | | |
|  |  |  |  |  | *Those who incurred costs* | *Median (IQR): $3.70* | | *Those who incurred costs* | | | | | *Median (IQR): $12.82* | | *Those who incurred costs* | *Median (IQR): $13.81* |  |  |  |  | *Those who incurred costs* | | | | *Median (IQR): $61.53* |  |  |  |  |  |  |
| *Ramma, 2015^48^* |  | | | | *Inpatient* | *Mean (SD): $0.66* | |  | | | | | | |  | |  | | | |  | | | | |  | | | | | |
|  |  |  |  |  |  | *Median (IQR): $0.00* | |  |  |  |  |  |  |  |  |  |  |  |  |  |  |  |  |  |  |  |  |  |  |  |  |
|  |  |  |  |  | *Outpatient* | *Mean (SD): $0.52* | |  |  |  |  |  |  |  |  |  |  |  |  |  |  |  |  |  |  |  |  |  |  |  |  |
|  |  |  |  |  |  | *Median (IQR): $0.00* | |  |  |  |  |  |  |  |  |  |  |  |  |  |  |  |  |  |  |  |  |  |  |  |  |
|  |  |  |  |  | *Intensive Phase* | *Mean (SD); $0.80* | |  |  |  |  |  |  |  |  |  |  |  |  |  |  |  |  |  |  |  |  |  |  |  |  |
|  |  |  |  |  |  | *Median (IQR): $0.00* | |  |  |  |  |  |  |  |  |  |  |  |  |  |  |  |  |  |  |  |  |  |  |  |  |
|  |  |  |  |  | *Continuation Phase* | *Mean (SD): $0.66* | |  |  |  |  |  |  |  |  |  |  |  |  |  |  |  |  |  |  |  |  |  |  |  |  |
|  |  |  |  |  |  | *Median (IQR): $0.00* | |  |  |  |  |  |  |  |  |  |  |  |  |  |  |  |  |  |  |  |  |  |  |  |  |
| *Razzaq, 20220* | *Diagnostics* | | *Median (IQR): $8.00 (0.30 – 18.20)* | |  |  | |  | | | | | |  |  |  |  | | |  |  | | | |  |  |  | |  | | |
|  | *Intensive Phase* | | *Median (IQR): $0.00 (0 – 0)* | |  |  |  |  |  |  |  |  |  |  |  |  |  |  |  |  |  |  |  |  |  |  |  |  |  |  |  |
|  | *Continuation Phase* | | *Median (IQR); $2.70 (1.30 – 9.00)* | |  |  |  |  |  |  |  |  |  |  |  |  |  |  |  |  |  |  |  |  |  |  |  |  |  |  |  |
| *Rupani, 2020*^49^* | *Private Provider* | *Median (IQR): $30.00 (10-76)* | | | *Private Provider* | *Median (IQR) $3.00 (0 – 7)* | | *Private Provider* | | | | | | *Median (IQR): $14.00 (5-55)* | *Private Provider* | *Median (IQR): $4.00 (0-7)* | *Private Provider* | | | *Median (IQR): $4.00 (0-7)* | *Private Provider* | | | | *Median (IQR): $0 (0-0)* | *Prescribed Nutrition* | *Private Provider* | | *Median (IQR): $0.00 (0-0)* | | |
|  | *Public Provider* | *Median (IQR): $0.00 (0-0)* | | | *Public Provider* | *Median (IQR): $0.00 (0 – 0)* | | *Public Provider* | | | | | | *Median (IQR): $0.00 (0-0)* | *Public Provider* | *Median (IQR): $0.00 (0-0)* | *Public Provider* | | | *Median (IQR); $0.00 (0-0)* | *Public Provider* | | | | *Median (IQR): $0.00 (0-0)* |  | *Public Provider* | | *Median (IQR): $0.00 (0-0)* | | |
|  | *Total* | *Median (IQR): $0.00 (0-0)* | | | *Total* | *Median (IQR): $0.00 (0-0)* | | *Total* | | | | | | *Median (IQR): $0.00 (0-0)* | *Total* | *Median (IQR): $0.00 (0-0)* | *Total* | | | *Median (IQR): $0.00 (0-0)* | *Total* | | | | *Median (IQR): $0.00 (0-0)* |  | *Total* | | *Median (IQR): $0.00 (0-0)* | | |
| *Rupani, 2022* | *TB* | *Median (IQR): $0.00 (0 – 15.00)* | | |  |  | | *TB* | | | | | | *Median (IQR): $0.00 (0 – 7.00)* |  |  | *TB* | | | *Median (IQR): $0.00 (0 – 4.00)* | *TB* | | | | *Median (IQR): $0.00 (0 – 15.00)* |  |  | |  | | |
|  | *TB/HIV* | *Median (IQR): $0.00 (0 – 25.00)* | | |  |  | | *TB/HIV* | | | | | | *Median (IQR): $0.00 (0 – 15.00)* |  |  | *TB/HIV* | | | *Median (IQR): $0.00 (0 – 4.00)* | *TB/HIV* | | | | *Median (IQR): $0.00 (0 – 1.70)* |  |  | |  | | |
| *Shin, 2020^50^* |  | | | | *Inpatient (Initial)* | *Mean (SD): $*0.07 | | *Inpatient (Initial)* | | | | | | *Mean (SD): $0.00* |  | |  | | | |  | | | | |  | | | | | |
|  |  |  |  |  | *Inpatient (Recurrent)* | *Mean (SD):* $0.27 | | *Inpatient (Recurrent)* | | | | | | *Mean (SD):* $1.13 |  |  |  |  |  |  |  |  |  |  |  |  |  |  |  |  |  |
|  |  |  |  |  | *Outpatient (HIV)* | *Mean (SD):* $0.90 | | *Outpatient (HIV)* | | | | | | *Mean (SD):* $0.31 |  |  |  |  |  |  |  |  |  |  |  |  |  |  |  |  |  |
|  |  |  |  |  | *Outpatient (TB)* | *-* | | *Outpatient (TB)* | | | | | | *-* |  |  |  |  |  |  |  |  |  |  |  |  |  |  |  |  |  |
| *Sweeney, 2018*^52^* | *Mean: $17.70* | | | | *Mean: $4.79* | | | *Mean: $2.91* | | | | | | | *Mean: $0.00* | | *Mean: $0.36* | | | | *Mean: $0.51* | | | | | *Traditional Healer* | | | | *Mean: $5.67* | |
|  |  |  |  |  |  |  |  |  |  |  |  |  |  |  |  |  |  |  |  |  |  |  |  |  |  | *DOT* | | | | *Mean: $0.00* | |
| *Timire, 2021^53^* | *DS-TB* | *Median (IQR): $72.29* | | |  | | |  | | | | | | |  | |  | | | |  | | | | |  | | | | | |
|  | *DR-TB* | *Median (IQR: $168.99* | | |  |  |  |  |  |  |  |  |  |  |  |  |  |  |  |  |  |  |  |  |  |  |  |  |  |  |  |
|  | *Total* | *Median (IQR): $74.45* | | |  |  |  |  |  |  |  |  |  |  |  |  |  |  |  |  |  |  |  |  |  |  |  |  |  |  |  |
| *Tomeny, 2020*^54^* | DS-TB | Mean:  $2.45 | | |  | | |  | | | | | | |  | |  | | | |  | | | | |  | | | | | |
|  | MDR-TB | Mean:  $2.19 | | |  |  |  |  |  |  |  |  |  |  |  |  |  |  |  |  |  |  |  |  |  |  |  |  |  |  |  |
| *Trajman, 2016*^55^* | *Minimum Wage* | *Mean (SD): $26.83 (34.35)* | | |  | | |  | | | | | | |  | |  | | | |  | | | | |  | | | | | |
|  | *Reported Income* | *Mean (SD): $34.35 (51.69)* | | |  |  |  |  |  |  |  |  |  |  |  |  |  |  |  |  |  |  |  |  |  |  |  |  |  |  |  |
| *Ukwaja, 2013^(1) 56^* |  | | | | *Mean: $0.53* | | |  | | | | | | |  | |  | | | | *Mean: $3.17* | | | | |  | | | | | |
| *Viney, 2019^59^* | *Mean (95% CI): $293.09* | | | |  | | |  | | | | | | |  | |  | | | |  | | | | |  | | | | | |
| *Viney, 2022* | Extra-pulmonary TB | | Median (IQR): $58.00 (0.00 – 76.00) | |  | | | Extra-pulmonary TB | | | | | Median (IQR): $0.00 (0 – 0) | |  | | Extra-pulmonary TB | | | Median (IQR): $0.00 (0 – 0) | Extra-pulmonary TB | | | Median (IQR): $58.00 (0 – 75.00) | | *DOT* | | Extra-pulmonary TB | | | Median (IQR): $0.00 (0 – 0) |
|  | Pulmonary TB | | Median (IQR): $36.00 (0.00 – 69.00) | |  |  |  | Pulmonary TB | | | | | Median (IQR): $0.00 (0 – 0) | |  |  | Pulmonary TB | | | Median (IQR): $0.00 (0 – 0) | Pulmonary TB | | | Median (IQR): $33.00 (0 – 62.00) | |  |  | Pulmonary TB | | | Median (IQR): $0.00 (0 – 0) |
|  | Total | | Median (IQR): $37.00 (0.00 – 71.00) | |  |  |  | Total | | | | | Median (IQR): $0.00 (0 – 0) | |  |  | Total | | | Median (IQR): $0.00 (0 – 0) | Total | | | Median (IQR): $36.00 (0 – 68.00) | |  |  | Total | | | Median (IQR): $0.00 (0 – 0) |
| *Vo, 2021* | ACF | | Mean (95% CI): $54 (39 – 70) | |  | | |  | | | | | | |  | |  | | | |  | | | | |  | | | | | |
|  |  |  | Median (IQR): $34 (22-61) | |  |  |  |  |  |  |  |  |  |  |  |  |  |  |  |  |  |  |  |  |  |  |  |  |  |  |  |
|  | PCF | | Mean (95% CI): $384 (76-693) | |  |  |  |  |  |  |  |  |  |  |  |  |  |  |  |  |  |  |  |  |  |  |  |  |  |  |  |
|  |  |  | Median (IQR): $76 (37-154) | |  |  |  |  |  |  |  |  |  |  |  |  |  |  |  |  |  |  |  |  |  |  |  |  |  |  |  |
|  | Total | | Mean (95% CI): $209 (63-355) | |  |  |  |  |  |  |  |  |  |  |  |  |  |  |  |  |  |  |  |  |  |  |  |  |  |  |  |
|  |  |  | Median (IQR): $47 (24-93) | |  |  |  |  |  |  |  |  |  |  |  |  |  |  |  |  |  |  |  |  |  |  |  |  |  |  |  |
| *Wang,2020^61^* | *Direct medical costs* | *Mean: $7,850.66* | | |  | | |  | | | | | | |  | |  | | | |  | | | | |  | | | | | |
|  |  | *Median (IQR): $6,900.75* | | |  |  |  |  |  |  |  |  |  |  |  |  |  |  |  |  |  |  |  |  |  |  |  |  |  |  |  |
|  | *OOP medical costs* | *Mean: $4,914.93* | | |  |  |  |  |  |  |  |  |  |  |  |  |  |  |  |  |  |  |  |  |  |  |  |  |  |  |  |
|  |  | *Median (IQR): $4,147.59* | | |  |  |  |  |  |  |  |  |  |  |  |  |  |  |  |  |  |  |  |  |  |  |  |  |  |  |  |
| *Yang, 2020*^62^* | *Hospitalized* | RS-TB | | Mean (SD): $  3,714.23 |  | | |  | | | | | | |  | |  | | | |  | | | | |  | | | | | |
|  |  |  |  | Median (IQR): $  2,521.78 |  |  |  |  |  |  |  |  |  |  |  |  |  |  |  |  |  |  |  |  |  |  |  |  |  |  |  |
|  |  | RMR-TB | | Mean (SD): $  15,000.89 |  |  |  |  |  |  |  |  |  |  |  |  |  |  |  |  |  |  |  |  |  |  |  |  |  |  |  |
|  |  |  |  | Median (IQR): $  7,644.25 |  |  |  |  |  |  |  |  |  |  |  |  |  |  |  |  |  |  |  |  |  |  |  |  |  |  |  |
|  |  | MDR-TB | | Mean (SD): $  17,139.85 |  |  |  |  |  |  |  |  |  |  |  |  |  |  |  |  |  |  |  |  |  |  |  |  |  |  |  |
|  |  |  |  | Median (IQR): $  14,093.21 |  |  |  |  |  |  |  |  |  |  |  |  |  |  |  |  |  |  |  |  |  |  |  |  |  |  |  |
|  | *Non-Hospitalized* | RS-TB | | Mean (SD): $  1,247.24 |  |  |  |  |  |  |  |  |  |  |  |  |  |  |  |  |  |  |  |  |  |  |  |  |  |  |  |
|  |  |  |  | Median (IQR): $  919.38 |  |  |  |  |  |  |  |  |  |  |  |  |  |  |  |  |  |  |  |  |  |  |  |  |  |  |  |
|  |  | RMR-TB | | Mean (SD): $  1,744.25 |  |  |  |  |  |  |  |  |  |  |  |  |  |  |  |  |  |  |  |  |  |  |  |  |  |  |  |
|  |  |  |  | Median (IQR): $  1,352.92 |  |  |  |  |  |  |  |  |  |  |  |  |  |  |  |  |  |  |  |  |  |  |  |  |  |  |  |
|  |  | MDR-TB | | Mean (SD): $  1,517.11 |  |  |  |  |  |  |  |  |  |  |  |  |  |  |  |  |  |  |  |  |  |  |  |  |  |  |  |
|  |  |  |  | Median (IQR): $  617.24 |  |  |  |  |  |  |  |  |  |  |  |  |  |  |  |  |  |  |  |  |  |  |  |  |  |  |  |
| *Abbreviations: TB – Tuberculosis, DS-TB – Drug sensitive TB, MDR-TB – Multi-drug resistant TB, SSM – Sputum smear microscopy, LJ – Löwenstein Jensen solid culture, LPA – Line probe assay, DR-TB – Drug resistant TB, RS-TB – Rifampicin sensitive TB, RMR-TB – Rifampicin mono-resistant TB, HIV – Human Immunodeficiency Virus, LTBI – Latent TB Infection, CHC -Community health centres, ACF – Active case finding, PCF – Passive case finding, SES – Socioeconomic status, DOT – Directly observed therapy, SD – Standard deviation, IQR – Interquartile range, CI – Confidence Interval*  **Costs reported are a combination of pre- and post-diagnostic costs* | | | | | | | | | | | | | | | | | | | | | | | | | | | | | | | |
