# Supplementary material for: The catastrophic cost of TB care: Understanding costs incurred by individuals undergoing TB care in low-, middle-, and high-income settings – A systematic review
Source: PLOS Glob Public Health. 2025 Apr 2;5(4):e0004283. doi: 10.1371/journal.pgph.0004283 (PMC12005564; doi:10.1371/journal.pgph.0004283)
Supplement: S6 Table — (DOCX) [file pgph.0004283.s012.docx]

## ***Table S6 – Breakdown of the direct non-medical costs incurred by patients during the post-diagnostic phase of TB care***

|  | *Total* | | | | | | *Transportation* | | | | | | | *Accommodation* | | | | | *Food* | | | | | | | *Nutritional Supplements* | | | | | | | | | | | *Other* | | | | | |  |  |
| --- | --- | --- | --- | --- | --- | --- | --- | --- | --- | --- | --- | --- | --- | --- | --- | --- | --- | --- | --- | --- | --- | --- | --- | --- | --- | --- | --- | --- | --- | --- | --- | --- | --- | --- | --- | --- | --- | --- | --- | --- | --- | --- | --- | --- |
| *Aia, 2022* | DS-TB | | | *Mean (95% CI): $137 (112.10 – 162.70)* | | | DS-TB | | *Mean (95% CI): $49.60 (40.50 – 58.80)* | | | | | DS-TB | | *Mean (95% CI): $25.60 (7.20 – 43.90)* | | | DS-TB | *Mean (95% CI): $62.10 (52.70 – 71.60)* | | | | | |  | | | | | | | | | | |  | | | | | |  |  |
|  | MDR-TB | | | *Mean (95% CI): $1,424 (330.50 – 2,516.50)* | | | MDR-TB | | *Mean (95% CI): $739 (266.70 – 1,744.40)* | | | | | MDR-TB | | *Mean (95% CI): $178 (92.20 – 263.30)* | | | MDR-TB | *Mean (95% CI): $507 (309.10 – 704.70)* | | | | | |  |  |  |  |  |  |  |  |  |  |  |  |  |  |  |  |  |  |  |
|  | Total | | | *Mean (95% CI): $162 (128.70 – 194.80)* | | | Total | | *Mean (95% CI): $62.70 (42.30 – 83.20)* | | | | | Total | | *Mean (95% CI): $28.50 (10.40 – 46.50)* | | | Total | *Mean (95% CI): $28.50 (10.40 – 46.50)* | | | | | |  | | | | | | | | | | |  | | | | | |  |  |
| *Aung, 2021^19^* |  | | | | | | *MDR-TB* | | *Median (min-max): $118.81* | | | | | *MDR-TB* | | *Median (min-max): $24.63* | | | *MDR-TB* | *Median (min-max): $626.98* | | | | | |  | | | | | | | | | | |  | | | | | |  |  |
|  |  |  |  |  |  |  | *DS-TB* | | *Median (min-max): $25.98* | | | | | *DS-TB* | | *Median (min-max): $1.53* | | | *DS-TB* | *Median (min-max): $92.56* | | | | | |  |  |  |  |  |  |  |  |  |  |  |  |  |  |  |  |  |  |  |
|  |  |  |  |  |  |  | *Total* | | *Median (min-max): $24.63* | | | | | *Total* | | *Median (min-max): $1.53* | | | *Total* | *Median (min-max): $101.04* | | | | | |  |  |  |  |  |  |  |  |  |  |  |  |  |  |  |  |  |  |  |
| *Bogdanova, 2019^20^* | *Culture Algorithm* | *BacTAlert (SSM+)* | | *Mean: $0.24* | | |  | | | | | | |  | | | | |  | | | | | | |  | | | | | | | | | | |  | | | | | |  |  |
|  |  | *LJ (SSM+)* | | *Mean: $0.24* | | |  |  |  |  |  |  |  |  |  |  |  |  |  |  |  |  |  |  |  |  |  |  |  |  |  |  |  |  |  |  |  |  |  |  |  |  |  |  |
|  |  | *LJ (SSM-)* | | *Mean: $0.27* | | |  |  |  |  |  |  |  |  |  |  |  |  |  |  |  |  |  |  |  |  |  |  |  |  |  |  |  |  |  |  |  |  |  |  |  |  |  |  |
|  | LPA Algorithm | LPA (SSM+) | | Mean: $0.24 | | |  |  |  |  |  |  |  |  |  |  |  |  |  |  |  |  |  |  |  |  |  |  |  |  |  |  |  |  |  |  |  |  |  |  |  |  |  |  |
|  |  | LPA+ Bactec MGIT (SSM-) | | Mean: $0.27 | | |  |  |  |  |  |  |  |  |  |  |  |  |  |  |  |  |  |  |  |  |  |  |  |  |  |  |  |  |  |  |  |  |  |  |  |  |  |  |
|  |  | LPA+LJ(SSM-) | | Mean: $0.27 | | |  |  |  |  |  |  |  |  |  |  |  |  |  |  |  |  |  |  |  |  |  |  |  |  |  |  |  |  |  |  |  |  |  |  |  |  |  |  |
| *Chandra, 2021^(2)22^* | *Median (IQR): $13.00(1.4-63)* | | | | | | *Median (IQR): 40.6 (0-2.50)* | | | | | | | *Median (IQR): $0* | | | | | *Food during travel* | *Median (IQR): $0* | | | | | |  | | | | | | | | | | |  |  |  |  |  |  |  |  |
|  |  |  |  |  |  |  |  |  |  |  |  |  |  |  |  |  |  |  |  | *Mean (SD): $0.15 (1.10)* | | | | | |  |  |  |  |  |  |  |  |  |  |  |  |  |  |  |  |  |  |  |
|  | Mean (SD): $41 (56) | | | | | | Mean (SD): $5 (5.20) | | | | | | | Mean (SD): $0 (0) | | | | | *Special diet* | *Median (IQR): $0 (0-35)* | | | | | |  |  |  |  |  |  |  |  |  |  |  |  |  |  |  |  |  |  |  |
|  |  |  |  |  |  |  |  |  |  |  |  |  |  |  |  |  |  |  |  | *Mean (SD): $27.00 (49)* | | | | | |  |  |  |  |  |  |  |  |  |  |  |  |  |  |  |  |  |  |  |
| *Chatterjee, 2023* |  | | | | | |  | | | | | | |  | | | | |  | | | | | | | *Intensive Phase* | | *Mean:*  *$3,679.14* | | | | | | | | |  | | | | | |  |  |
|  |  |  |  |  |  |  |  |  |  |  |  |  |  |  |  |  |  |  |  |  |  |  |  |  |  | *Continuation Phase* | | *Mean: $4,488.28* | | | | | | | | |  |  |  |  |  |  |  |  |
|  |  |  |  |  |  |  |  |  |  |  |  |  |  |  |  |  |  |  |  |  |  |  |  |  |  | *Post-Treatment Phase* | | *Mean: $214.29* | | | | | | | | |  |  |  |  |  |  |  |  |
| *Chittamany, 2020^23^* | *DS-TB* | | | *Median (IQR): $520.18* | | | *DS-TB* | | *Median (IQR): $52.50* | | | | | *DS-TB* | | *Median (IQR): $0 (0-0)* | | | *DS-TB* | *Median (IQR): $0* | | | | | | *DS-TB* | | | | | *Median (IQR): $364.29* | | | | | | *DS-TB* | | | *Median (IQR): $0* | | | |  |
|  | *DR-TB* | | | *Median (IQR): $423.15* | | | *DR-TB* | | *Median (IQR): $135.22* | | | | | *DR-TB* | | *Median (IQR): $0 (0-0)* | | | *DR-TB* | *Median (IQR): $39.77* | | | | | | *DR-TB* | | | | | *Median (IQR): $256.11* | | | | | | *DR-TB* | | | *Median (IQR): $15.91* | | | |  |
|  | *Total* | | | *Median (IQR): $520.18* | | | *Total* | | *Median (IQR): $52.50* | | | | | *Total* | | *Median (IQR): $0 (0-0)* | | | *Total* | *Median (IQR): $ 1.59* | | | | | | *Total* | | | | | *Median (IQR):$354.74* | | | | | | *Total* | | | *Median (IQR): $0* | | | |  |
| *Collins, 2018^24^* |  | | | | | | *MDR-TB* | | *Addis Ababa* | | *Median (IQM): $143.74* | | |  | | | | | *MDR-TB* | *Addis Ababa* | | | *Median (IQM): $489.95* | | |  | | | | | | | | | | |  | | | | | |  |  |
|  |  |  |  |  |  |  |  |  | *Gonder* | | *Median (IQM): $11.25* | | |  | | | | |  | *Gonder* | | | *Median (IQM): $66.24* | | |  |  |  |  |  |  |  |  |  |  |  |  |  |  |  |  |  |  |  |
| *Diallo, 2022* |  | | | |  | | DS-TB | | Mean (95% CI): $19.1 (12.49 to 25.72) | | | | | DS-TB | | Mean (95% CI): $0.24 (0.01 to –0.486) | | | DS-TB | Mean (95% CI): $26.5 (13.09 to 39.95) | | | | | | DS-TB | | | Mean (95% CI): $44.4 (15.06 to 73.71) | | | | | | | |  | | | | | |  |  |
|  |  |  |  |  |  |  | DR-TB | | Mean (95% CI): $28.6 (6.42 to 50.79) | | | | | DR-TB | | Mean (95% CI): $0.0 (0.0 to 0.0 | | | DR-TB | Mean (95% CI): $38.9 (–19.68 to –97.42) | | | | | | DR-TB | | | Mean (95% CI): $33.3 (–35.66 to 102) | | | | | | | |  |  |  |  |  |  |  |  |
|  |  |  |  |  |  |  | Total | | Mean (95% CI): $19.3 (12.8 to 25.74) | | | | | Total | | Mean (95% CI): $0.24 (,0.01 to 0.47) | | | Total | Mean (95% CI): $26.8 (13.2 to 40.26) | | | | | | Total | | | Mean (95% CI): $44.19 (15.21 to 73.17) | | | | | | | |  |  |  |  |  |  |  |  |
| *De Siqueria Filha, 2018^25^* | *TB/HIV* | | | | *Mean: $45.60* | | *TB/HIV* | | *Mean: $22.99* | | | | |  | | | | | *TB/HIV* | *Mean: $ 22.61* | | | | | |  | | | | | | | | | | |  | | | | | |  |  |
|  | *LTBI/HIV* | | | | *Mean: $31.66* | | *LTBI/HIV* | | *Mean: $1.63* | | | | |  |  |  |  |  | *LTBI/HIV* | *Mean: $1.63* | | | | | |  |  |  |  |  |  |  |  |  |  |  |  |  |  |  |  |  |  |  |
| *Devoid, 2022* |  | | | | | | *Intensive Phase* | | *Mean (SD): $5.78 (7.28)* | | | | |  | | | | | *Intensive Phase* | *Mean (SD): $3.98 (9.29)* | | | | | |  | | | | | | | | | | |  | | | | | |  |  |
|  |  |  |  |  |  |  | *Continuation Phase* | | *Mean (SD): $9.25 (13.08)* | | | | |  |  |  |  |  | *Continuation Phase* | *Mean (SD): $7.79 (15.55)* | | | | | |  |  |  |  |  |  |  |  |  |  |  |  |  |  |  |  |  |  |  |
| *Ellaban 2021^26^* |  | | |  | | | *First two months of treatment (intensive phase)* | | *Median (IQR): $5.00 (2.60 – 9.40)* | | | | |  | | | | | *First two months of treatment (intensive phase)* | *Median (IQR): $0.00 (0.00 – 1.60)* | | | | | |  | | | | | | | | | | |  | | | | | |  |  |
|  |  | | |  | | | *Second two months of treatment* | | *Median (IQR): $3.80 (1.90 – 5.60)* | | | | |  |  |  |  |  | *Second two months of treatment* | *Median (IQR): $0.00 (0.00 - 0.00)* | | | | | |  |  |  |  |  |  |  |  |  |  |  |  |  |  |  |  |  |  |  |
|  |  | | |  | | | *Third two months of treatment* | | *Median (IQR): $1.90 (1.30 – 3.80)* | | | | |  |  |  |  |  | *Third two months of treatment* | *Median (IQR): $0.00 (0.00 – 0.00)* | | | | | |  |  |  |  |  |  |  |  |  |  |  |  |  |  |  |  |  |  |  |
| *Florentino, 2022* |  | | | | | | *Urban DS-TB* | | *Mean (SD): $35.80 (79.20)* | | | | | *Urban DS-TB* | | *Mean (SD): $7.20 (101.90)* | | | *Urban DS-TB* | *Mean (SD): $18.80 (63.10)* | | | | | | *Urban DS-TB* | | | *Mean (SD): $ 113.30 (174.60)* | | | | | | | |  | | | | | |  |  |
|  |  |  |  |  |  |  | *Rural DS-TB* | | *Mean (SD): $93.00 (370.20)* | | | | | *Rural DS-TB* | | *Mean (SD): $1.90 (33.00)* | | | *Rural DS-TB* | *Mean (SD): $44.10 (125.30)* | | | | | | *Rural DS-TB* | | | *Mean (SD): $104.80 (136.60)* | | | | | | | |  |  |  |  |  |  |  |  |
|  |  |  |  |  |  |  | *DR-TB* | | *Mean (SD): $774.20 (967.50)* | | | | | *DR-TB* | | *Mean (SD): $13.10 (92.80)* | | | *DR-TB* | *Mean (SD): $356.20 (469.70)* | | | | | | *DR-TB* | | | *Mean (SD): $472.60 (511.80)* | | | | | | | |  |  |  |  |  |  |  |  |
|  |  |  |  |  |  |  | *Total* | | *Mean (SD): $94.10 (476.50* | | | | | *Total* | | *Mean (SD): $2.90 (49.30)* | | | *Total* | *Mean (SD): $44.80 (164.30)* | | | | | | *Total* | | | *Mean (SD): $111.90 (196)* | | | | | | | |  |  |  |  |  |  |  |  |
| *Fuady, 2018^29^* | *DS-TB* | | | *Median (IQR): $11.36* | | | *DS-TB* | | *Median (IQR): $2.41* | | | | |  | | | | | *DS-TB* | *Median (IQR): $0.00* | | | | | | *DS-TB* | | | | | *Median (IQR): $4.47 (0.00 – 23.06)* | | | | | |  | | | | | |  |  |
|  | *MDR-TB* | | | *Median (IQR): $294.56* | | | *MDR-TB* | | *Median (IQR): $138.68* | | | | |  |  |  |  |  | *MDR-TB* | *Median (IQR): $92.57* | | | | | | *MDR-TB* | | | | | *Median (IQR): $61.59 (1.38 – 119.41)* | | | | | |  |  |  |  |  |  |  |  |
| *Getahun, 2016^30^* |  | | | | | | Intensive Phase | | Mean (SD): $6.27 | | | | |  | | | | | Intensive Phase | Mean (SD): $13.73 | | | | | |  | | | | | | | | | | | Additional Food | | | Mean (SD): $72.36 | | | |  |
|  |  |  |  |  |  |  |  |  | Median (R): $4.90 | | | | |  |  |  |  |  |  | Median (R): $12.27 | | | | | |  |  |  |  |  |  |  |  |  |  |  |  |  |  |  |  |  |  |  |
|  |  |  |  |  |  |  | Continuation Phase | | Mean (SD): $2.28 | | | | |  |  |  |  |  | Continuation Phase | Mean (SD): $5.00 | | | | | |  |  |  |  |  |  |  |  |  |  |  |  |  |  |  |  |  |  |  |
|  |  |  |  |  |  |  |  |  | Median (R): $1.78 | | | | |  |  |  |  |  |  | Median (R): $4.46 | | | | | |  |  |  |  |  |  |  |  |  |  |  |  |  |  | Median (R): $97.00 | | | |  |
|  |  |  |  |  |  |  | Total | | Mean (SD): $8.55 | | | | |  |  |  |  |  | Total | Mean (SD): $11.27 | | | | | |  |  |  |  |  |  |  |  |  |  |  |  |  |  |  |  |  |  |  |
|  |  |  |  |  |  |  |  |  | Median (R): $6.69 | | | | |  |  |  |  |  |  | Median (R): $8.91 | | | | | |  |  |  |  |  |  |  |  |  |  |  |  |  |  |  |  |  |  |  |
| *Gospodarevskaya, 2014^31^* |  | | | | | |  | | | | | | |  | | | | | *Tanzania* | First two months of treatment | | | | Mean: $2.89 | |  | | | | | | | | | | |  | | | | | |  |  |
|  |  |  |  |  |  |  |  |  |  |  |  |  |  |  |  |  |  |  |  | Most recent two months of treatment | | | | Mean: $1.65 | |  | | | | | | | | | | |  |  |  |  |  |  |  |  |
|  |  |  |  |  |  |  |  |  |  |  |  |  |  |  |  |  |  |  |  | Total treatment costs | | | | Mean: $6.19 | |  | | | | | | | | | | |  |  |  |  |  |  |  |  |
|  |  |  |  |  |  |  |  |  |  |  |  |  |  |  |  |  |  |  | *Bangladesh* | First two months of treatment | | | | Mean: $20.80 | |  | | | | | | | | | | |  |  |  |  |  |  |  |  |
|  |  |  |  |  |  |  |  |  |  |  |  |  |  |  |  |  |  |  |  | Most recent two months of treatment | | | | Mean: $14.09 | |  | | | | | | | | | | |  |  |  |  |  |  |  |  |
|  |  |  |  |  |  |  |  |  |  |  |  |  |  |  |  |  |  |  |  | Total treatment cost | | | | Mean: $48.97 | |  | | | | | | | | | | |  |  |  |  |  |  |  |  |
| *Gurung, 2019^33^* | *ACF* | | | *Median (IQR): $0.00* | | | *ACF* | | *Median (IQR): $0.00* | | | | |  | | | | | *ACF* | *Median (IQR): $0.00* | | | | | | *ACF* | | | | | | | | | *Median (IQR): $14.76* | |  | | | | | |  |  |
|  | *PCF* | | | *Median (IQR): $1.41* | | | *PCF* | | *Median (IQR): $0.43* | | | | |  |  |  |  |  | *PCF* | *Median (IQR): $0.00* | | | | | | *PCF* | | | | | | | | | *Median (IQR): $16.82* | |  |  |  |  |  |  |  |  |
|  | *Total* | | | *Median (IQR): $0.00* | | | *Total* | | *Median (IQR): $0.00* | | | | |  |  |  |  |  | *Total* | *Median (IQR): $0.00* | | | | | | *Total* | | | | | | | | | *Median (IQR): $16.17* | |  |  |  |  |  |  |  |  |
| *Gurung, 2021^32^* | *ACF* | | | *Mean (95% CI): $7.60 (5.10 – 10.20)* | | |  | | | | | | |  | | | | |  | | | | | | |  | | | | | | | | | | |  | | | | | |  |  |
|  |  |  |  | *Median (IQR) $1.90 (0.30 – 9.40)* | | |  |  |  |  |  |  |  |  |  |  |  |  |  |  |  |  |  |  |  |  |  |  |  |  |  |  |  |  |  |  |  |  |  |  |  |  |  |  |
|  | *PCF* | | | *Mean (95% CI): $9.50 (6.80 – 12.10)* | | |  |  |  |  |  |  |  |  |  |  |  |  |  |  |  |  |  |  |  |  |  |  |  |  |  |  |  |  |  |  |  |  |  |  |  |  |  |  |
|  |  |  |  | *Median (IQR): $3.40 (0.70 – 13.20)* | | |  |  |  |  |  |  |  |  |  |  |  |  |  |  |  |  |  |  |  |  |  |  |  |  |  |  |  |  |  |  |  |  |  |  |  |  |  |  |
|  | *Total* | | | *Mean (95% CI): $8.60 (6.70 – 10.40)* | | |  |  |  |  |  |  |  |  |  |  |  |  |  |  |  |  |  |  |  |  |  |  |  |  |  |  |  |  |  |  |  |  |  |  |  |  |  |  |
|  |  |  |  | *Median (IQR): $2.70 (0.70 – 10.80)* | | |  |  |  |  |  |  |  |  |  |  |  |  |  |  |  |  |  |  |  |  |  |  |  |  |  |  |  |  |  |  |  |  |  |  |  |  |  |  |
| *Kaswa, 2022* | DS-TB | | | *Mean (95% CI): $171.00 (129.30 – 213.40)* | | | DS-TB | *Mean (95% CI): $73.00 (49.30 – 96.50)* | | | | | | DS-TB | *Mean (95% CI): $2.80 (1.40 – 4.10)* | | | | DS-TB | *Mean (95% CI): $58.00 (32.80 – 84.10)* | | | | | | DS-TB | | | | *Mean (95% CI): $37.00 (23.20 – 51.20)* | | | | | | | |  | | | | | |  |
|  | DR-TB | | | *Mean (95% CI): $568.00 (328.20 – 808.40)* | | | DR-TB | *Mean (95% CI): $295.00 (174.90 – 415.70)* | | | | | | DR-TB | *Mean (95% CI): $13.00 (-14.70 – 40.10)* | | | | DR-TB | *Mean (95% CI): $184.00 (67.80 – 300.30)* | | | | | | DR-TB | | | | *Mean (95% CI): $76.00 (31.30 – 121.00)* | | | | | | | |  | | | | | |  |
|  | Total | | | *Mean (95% CI): $243.00 (186.90 – 299.30)* | | | Total | *Mean (95% CI): $113.00 (81.50 – 144.80)* | | | | | | Total | *Mean (95% CI): $4.60 (-0.80 – 9.90)* | | | | Total | *Mean (95% CI): $81.00 (48.10 – 114.20)* | | | | | | Total | | | | *Mean (95% CI): $44.00 (27.50 – 61.10)* | | | | | | | |  | | | | | |  |
| *Kilale, 2022* |  | | |  | | | *Mean (SD): $30.10 (37.10)* | | | | | | | *Mean (SD): $0.70 (3.30)* | | | | | *Mean (SD): $13.80 (36.50)* | | | | | | | *Mean (SD): $85.80 (91.70)* | | | | | | | | | | |  | | | | | |  |  |
|  |  |  |  |  |  |  | *Median (IQR): $19.80 (9.20 – 39.10)* | | | | | | | *Median (IQR): $0.00 (0.00 -0.00)* | | | | | *Median (IQR): 5.00 (0.00 – 14.30)* | | | | | | | *Median (IQR): $72.80 (0.00 – 121.40)* | | | | | | | | | | |  |  |  |  |  |  |  |  |
| *Kirubi, 2021^34^* | *Median (IQR): $170.85* | | | | | |  | | | | | | |  | | | | |  | | | | | | |  | | | | | | | | | | |  | | | | | |  |  |
| *Loureiro, 2024* | *Mean: $58.62* | | | | | | Mean: $21.19 | | | | | | | Mean: $0.00 | | | | | Mean: $6.61 | | | | | | |  | | | | | | | | | | |  | | | | | |  |  |
|  |  |  |  |  |  |  |  |  |  |  |  |  |  |  |  |  |  |  | Special Food | | Mean: $30.82 | | | | |  |  |  |  |  |  |  |  |  |  |  |  |  |  |  |  |  |  |  |
| *Lu, 2020^35^* | *Residents* | | | *Mean: $230.53* | | |  | | | | | | |  | | | | |  | | | | | | |  | | | | | | | | | | |  | | | | | |  |  |
|  | *Migrants* | | | *Mean: $113.31* | | |  |  |  |  |  |  |  |  |  |  |  |  |  |  |  |  |  |  |  |  |  |  |  |  |  |  |  |  |  |  |  |  |  |  |  |  |  |  |
| *Maciel, 2023* |  | | | | | | DS-TB | | | | | | Mean (95% CI): $75.95 (59.77 – 92.25) | DS-TB | | | | Mean (95% CI): $4.05 (2.31 – 5.66) | DS-TB | | | Mean (95% CI): $13.76 (8.55 – 19.09) | | | | DS-TB | | | | | | | | Mean (95% CI): $292.94 (214.67 – 371.09) | | |  | | | | | |  |  |
|  |  |  |  |  |  |  | DR-TB | | | | | | Mean (95% CI): $220.45 (134.68 – 306.23) | DR-TB | | | | Mean (95% CI): $7.63 (2.43 – 17.69) | DR-TB | | | Mean (95% CI): $54.91 (26.36 – 83.47) | | | | DR-TB | | | | | | | | Mean (95% CI): $982.05 (599.75 – 1,364.35) | | |  |  |  |  |  |  |  |  |
|  |  |  |  |  |  |  | Total | | | | | | Mean (95% CI): $91.56 (71.56 – 111.56) | Total | | | | Mean (95% CI): $4.39 (2.43 – 6.36) | Total | | | Mean (95% CI): $18.27 (11.33 – 25.09) | | | | Total | | | | | | | | Mean (95% CI): $367.16 (269.01 – 465.42) | | |  |  |  |  |  |  |  |  |
| *Mafirakureva, 2023 ^2^* |  | | | | | | *Cameroon* | | | | | | Median (IQR): $17.47 (3.30 – 48.46) | *Cameroon* | | | | Median (IQR): $0.00 (0.00 – 0.00) | *Cameroon* | | | Median (IQR): $0.00 (0.00 – 0.00) | | | | *Cameroon* | | | | | | | | Median (IQR): $0.00 (0.00 – 5.89) | | |  | | | | | |  |  |
|  |  |  |  |  |  |  | *Kenya* | | | | | | Median (IQR): $2.26 (0.00 – 22.27) | *Kenya* | | | | Median (IQR): $0.00 (0.00 -0.00) | *Kenya* | | | Median (IQR): $0.00 (0.00 – 0.00) | | | | *Kenya* | | | | | | | | Median (IQR): $31.16 (0.00 – 84.73) | | |  |  |  |  |  |  |  |  |
| *Mauch, 2013^(1) 36^* |  | | | | | |  | | | | | | |  | | | | | *Ghana* | *Mean: $2.49* | | | | | |  | | | | | | | | | | |  | | | | | |  |  |
|  |  |  |  |  |  |  |  |  |  |  |  |  |  |  |  |  |  |  |  | *Median (IQR): $1.61* | | | | | |  |  |  |  |  |  |  |  |  |  |  |  |  |  |  |  |  |  |  |
|  |  |  |  |  |  |  |  |  |  |  |  |  |  |  |  |  |  |  | *Vietnam* | *Mean: $4.94* | | | | | |  |  |  |  |  |  |  |  |  |  |  |  |  |  |  |  |  |  |  |
|  |  |  |  |  |  |  |  |  |  |  |  |  |  |  |  |  |  |  |  | *Median (IQR): $2.69* | | | | | |  |  |  |  |  |  |  |  |  |  |  |  |  |  |  |  |  |  |  |
|  |  |  |  |  |  |  |  |  |  |  |  |  |  |  |  |  |  |  | *Dominican Republic* | *Mean: $4.22* | | | | | |  |  |  |  |  |  |  |  |  |  |  |  |  |  |  |  |  |  |  |
|  |  |  |  |  |  |  |  |  |  |  |  |  |  |  |  |  |  |  |  | *Median (IQR): $1.61* | | | | | |  |  |  |  |  |  |  |  |  |  |  |  |  |  |  |  |  |  |  |
| *Mauch, 2013^(2) 38^* |  | | | | | |  | | | | | | |  | | | | | *New* | *Median: $17.49* | | | | | |  | | | | | | | | | | |  | | | | | |  |  |
|  |  |  |  |  |  |  |  |  |  |  |  |  |  |  |  |  |  |  | *Retreatment* | *Median: $18.12* | | | | | |  |  |  |  |  |  |  |  |  |  |  |  |  |  |  |  |  |  |  |
|  |  |  |  |  |  |  |  |  |  |  |  |  |  |  |  |  |  |  | *MDR* | *Median: $22.54* | | | | | |  |  |  |  |  |  |  |  |  |  |  |  |  |  |  |  |  |  |  |
| *McAllister, 2020^39^* |  | | | | | | *CHC* | | *Median (IQR): $5.47* | | | | | | | | | |  | *CHC* | | | | | *Median (IQR): $2.58* |  | | | | | | | | | | |  | | | | | |  |  |
|  |  |  |  |  |  |  | *Public Hospital* | | *Median (IQR): $15.45* | | | | | | | | | |  | *Public Hospital* | | | | | *Median (IQR): $6.41* |  |  |  |  |  |  |  |  |  |  |  |  |  |  |  |  |  |  |  |
|  |  |  |  |  |  |  | *Private Hospital* | | *Median (IQR): $8.31* | | | | | | | | | |  | *Private Hospital* | | | | | *Median (IQR): $6.59* |  |  |  |  |  |  |  |  |  |  |  |  |  |  |  |  |  |  |  |
|  |  |  |  |  |  |  | *Private Practice* | | *Median (IQR): $10.64* | | | | | | | | | |  | *Private Practice* | | | | | *Median (IQR): $2.31* |  |  |  |  |  |  |  |  |  |  |  |  |  |  |  |  |  |  |  |
| *Morishita, 2016^40^* |  | | | | | | *DOT* | | *ACF* | | *Mean (SD): $4.84* | | |  | | | | | *ACF* | *Mean (SD): $33.50* | | | | | |  | | | | | | | | | | |  | | | | | |  |  |
|  |  |  |  |  |  |  |  |  |  |  | *Median (IQR): $0.00* | | |  |  |  |  |  |  |  |  |  |  |  |  |  |  |  |  |  |  |  |  |  |  |  |  |  |  |  |  |  |  |  |
|  |  |  |  |  |  |  |  |  | *PCF* | | *Mean (SD): $15.35* | | |  |  |  |  |  |  |  |  |  |  |  |  |  |  |  |  |  |  |  |  |  |  |  |  |  |  |  |  |  |  |  |
|  |  |  |  |  |  |  |  |  |  |  | *Median (IQR): $0.00* | | |  |  |  |  |  |  | *Median (IQR): $22.94* | | | | | |  |  |  |  |  |  |  |  |  |  |  |  |  |  |  |  |  |  |  |
|  |  |  |  |  |  |  | *Drug pick-up* | | *ACF* | | *Mean (SD): $9.58* | | |  |  |  |  |  |  |  |  |  |  |  |  |  |  |  |  |  |  |  |  |  |  |  |  |  |  |  |  |  |  |  |
|  |  |  |  |  |  |  |  |  |  |  | *Median (IQR): $7.65* | | |  |  |  |  |  |  |  |  |  |  |  |  |  |  |  |  |  |  |  |  |  |  |  |  |  |  |  |  |  |  |  |
|  |  |  |  |  |  |  |  |  | *PCF* | | *Mean (SD): $8.57* | | |  |  |  |  |  | *PCF* | *Mean (SD): $47.36* | | | | | |  |  |  |  |  |  |  |  |  |  |  |  |  |  |  |  |  |  |  |
|  |  |  |  |  |  |  |  |  |  |  | *Median (IQR): $6.12* | | |  |  |  |  |  |  |  |  |  |  |  |  |  |  |  |  |  |  |  |  |  |  |  |  |  |  |  |  |  |  |  |
|  |  |  |  |  |  |  | *Follow-up examination* | | *ACF* | | *Mean (SD): $0.46* | | |  |  |  |  |  |  |  |  |  |  |  |  |  |  |  |  |  |  |  |  |  |  |  |  |  |  |  |  |  |  |  |
|  |  |  |  |  |  |  |  |  |  |  | *Median (IQR): $0.00* | | |  |  |  |  |  |  | *Median (IQR): $67.55* | | | | | |  |  |  |  |  |  |  |  |  |  |  |  |  |  |  |  |  |  |  |
|  |  |  |  |  |  |  |  |  | *PCF* | | *Mean (SD): $0.41* | | |  |  |  |  |  |  |  |  |  |  |  |  |  |  |  |  |  |  |  |  |  |  |  |  |  |  |  |  |  |  |  |
|  |  |  |  |  |  |  |  |  |  |  | *Median (IQR): $0.00* | | |  |  |  |  |  |  |  |  |  |  |  |  |  |  |  |  |  |  |  |  |  |  |  |  |  |  |  |  |  |  |  |
| *Mudzengi, 2017^41^* |  | | | | | | *Study Clinic* | | *TB/HIV* | | *Mean (SD): $*  1.98 | | |  | | | | | *Hospital* | *TB/HIV* | | | *Mean (SD): $*  0.13 | | |  | | | | | | | | | | |  | | | | | |  |  |
|  |  |  |  |  |  |  |  |  | *TB* | | *Mean (SD): $*  0.81 | | |  |  |  |  |  |  | *TB* | | | *Mean (SD): $*0.00 | | |  |  |  |  |  |  |  |  |  |  |  |  |  |  |  |  |  |  |  |
|  |  |  |  |  |  |  |  |  | *HIV* | | *Mean (SD): $*  0.60 | | |  |  |  |  |  |  | *HIV* | | | *Mean (SD): $*  0.02 | | |  |  |  |  |  |  |  |  |  |  |  |  |  |  |  |  |  |  |  |
|  |  |  |  |  |  |  | *Other Facilities* | | *TB/HIV* | | *Mean (SD): $*  0.30 | | |  |  |  |  |  | *Special Diet* | *TB/HIV* | | | *Mean (SD): $*  6.32 | | |  |  |  |  |  |  |  |  |  |  |  |  |  |  |  |  |  |  |  |
|  |  |  |  |  |  |  |  |  | *TB* | | *Mean (SD): $*  0.02 | | |  |  |  |  |  |  | *TB* | | | *Mean (SD): $*  3.88 | | |  |  |  |  |  |  |  |  |  |  |  |  |  |  |  |  |  |  |  |
|  |  |  |  |  |  |  |  |  | *HIV* | | *Mean (SD): $*  0.12 | | |  |  |  |  |  |  | *HIV* | | | *Mean (SD): $*  4.69 | | |  |  |  |  |  |  |  |  |  |  |  |  |  |  |  |  |  |  |  |
| *Muniyandi, 2020^42^* |  | | | | | | *Mean (SD): $1.16 (1.92)* | | | | | | |  | | | | | *Mean (SD): $0.30 (0.98)* | | | | | | |  | | | | | | | | | | |  | | | | | |  |  |
|  |  |  |  |  |  |  | *Median (IQR): $0.00 (0.00 – 16.04)* | | | | | | |  |  |  |  |  | *Median (IQR): $0.00 (0.00 – 12.03)* | | | | | | |  |  |  |  |  |  |  |  |  |  |  |  |  |  |  |  |  |  |  |
| *Muttamba, 2020^43^* |  | | | | | | *MDR-TB* | | *Mean (95% CI): $408.03 (358.78 – 457.68)* | | | | | *MDR-TB* | | *Mean (95% CI): $0.16* | | | *MDR-TB* | *Mean (95% CI): $199.41* | | | | | *MDR-TB* | *Mean (95% CI): $505.73* | | | | | | | | | | |  | | | | | |  |  |
|  |  |  |  |  |  |  | *DS-TB* | | *Mean (95% CI): $17.58* | | | | | *DS-TB* | | *Mean (95% CI): $0.56* | | | *DS-TB* | *Mean (95% CI): $12.25* | | | | | *DS-TB* | *Mean (95% CI): $75.68* | | | | | | | | | | |  |  |  |  |  |  |  |  |
|  |  |  |  |  |  |  | *Total* | | *Mean (95% CI): $31.99* | | | | | *Total* | | *Mean (95% CI): $0.56* | | | *Total* | *Mean (95% CI): $19.18* | | | | | *Total* | *Mean (95% CI): $219.36* | | | | | | | | | | |  |  |  |  |  |  |  |  |
| *Nhung, 2018^44^* | *MDR-TB* | | | *Mean (95% CI): $8,649.44* | | | *MDR-TB* | | *Mean (95% CI): $1,730.77* | | | | | *MDR-TB* | | *Mean (95% CI); $188.89* | | | *MDR-TB* | *Mean (95% CI): $4,647.59* | | | | | |  | | | | | | | | | | |  | | | | | |  |  |
|  | *DS-TB* | | | *Mean (95% CI): $1,629.73* | | | *DS-TB* | | *Mean (95% CI): $158.14* | | | | | *DS-TB* | | *Mean (95% CI): $20.65* | | | *DS-TB* | *Mean (95% CI); $1,010.35* | | | | | |  |  |  |  |  |  |  |  |  |  |  |  |  |  |  |  |  |  |  |
|  | *Total* | | | *Mean (95% CI): $2,196.41* | | | *Total* | | *Mean (95% CI): $285.53* | | | | | *Total* | | *Mean (95% CI): $34.26* | | | *Total* | *Mean (95% CI): $1300.27* | | | | | |  |  |  |  |  |  |  |  |  |  |  |  |  |  |  |  |  |  |  |
| *Pedrazzoli, 2018^45^* | *MDR-TB* | | | *Median (IQR): $387.86* | | | *MDR-TB* | | *Median (IQR): $8.24* | | | | | *MDR-TB* | | *Median (IQR): $0.00* | | | *MDR-TB* | *Median (IQR): $69.26* | | | | | *MDR-TB* | *Median (IQR): $144.05* | | | | | | | | | | |  | | | | | |  |  |
|  | *DS-TB* | | | *Median (IQR): $146.39* | | | *DS-TB* | | *Median (IQR): $5.56* | | | | | *DS-TB* | | *Median (IQR): $0.00* | | | *DS-TB* | *Median (IQR): $20.79* | | | | | *DS-TB* | *Median 9IQR): $30.39* | | | | | | | | | | |  |  |  |  |  |  |  |  |
|  | *Total* | | | *Median (IQR): $157.81* | | | *Total* | | *Median (IQR): $5.56* | | | | | *Total* | | *Median (IQR): $0.00* | | | *Total* | *Median (IQR): $23.76* | | | | | *Total* | *Median (IQR): $39.81* | | | | | | | | | | |  |  |  |  |  |  |  |  |
| *Pedrazzoli, 2021^46^* | *Uninsured* | | | *Mean (SD): $137.97* | | |  | | | | | | |  | | | | |  | | | | | | |  | | | | | | | | | | |  | | | | | |  |  |
|  |  |  |  | *Median (IQR): $45.89* | | |  |  |  |  |  |  |  |  |  |  |  |  |  |  |  |  |  |  |  |  |  |  |  |  |  |  |  |  |  |  |  |  |  |  |  |  |  |  |
|  | *Insured* | | | *Mean (SD): $133.11* | | |  |  |  |  |  |  |  |  |  |  |  |  |  |  |  |  |  |  |  |  |  |  |  |  |  |  |  |  |  |  |  |  |  |  |  |  |  |  |
|  |  |  |  | *Median (IQR): $46.19* | | |  |  |  |  |  |  |  |  |  |  |  |  |  |  |  |  |  |  |  |  |  |  |  |  |  |  |  |  |  |  |  |  |  |  |  |  |  |  |
| *Pham, 2023* |  | | |  | | | *MDR-TB* | | Median (IQR): $50.50 (30.20 – 76.92) | | | | | *MDR-TB* | | | | Median (IQR): $0.00 | *MDR-TB* | Median (IQR): $36.82 (3.27 – 122.13) | | | | | | *MDR-TB* | | | | Median (IQR): $386.54 (195.11 – 679.37) | | | | | | | *MDR-TB* | | | | Median (IQR): $0.00 (0.00 – 4.70) | |  |  |
| *Prasanna, 2018^47^* |  | | | | | | *Study population* | | *Median (IQR): $6.66* | | | | | *Study population* | | | | *Median (IQR): $0.00* | *Study population* | *Median (IQR): $0.00* | | | | | | *Study population* | | | | *Median (IQR): $18.37* | | | | | | | *Comorbidities* | | | *Study population* | | *Median (IQR): $0.00* | |  |
|  |  |  |  |  |  |  | *Those who incurring costs* | | *Median (IQR): $8.14* | | | | | *Those who incurring costs* | | | | *Median (IQR): $110.11* | *Those who incurring costs* | *Median (IQR): $8.14* | | | | | | *Those who incurring costs* | | | | *Median (IQR): $22.07* | | | | | | |  |  |  | *Those who incurring costs* | | *Median (IQR): $17.39* | |  |
| *Ramma, 2015^48^* |  | | | | | | *Inpatients* | | *Mean (SD): $2.50* | | | | | | | | | |  |  | | | | | | *Inpatients* | | | | *Mean (SD): $5.95* | | | | | | | *Miscellaneous* | | | *Inpatients* | | *Mean (SD): $3.21* | |  |
|  |  |  |  |  |  |  |  |  | *Median (IQR): $0.00* | | | | | | | | | |  |  |  |  |  |  |  |  |  |  |  | *Median (IQR): $0.71* | | | | | | |  |  |  |  |  | *Median (IQR): $0.00* | |  |
|  |  |  |  |  |  |  | *Outpatients* | | *Mean (SD): $1.70* | | | | | | | | | |  |  |  |  |  |  |  | *Outpatients* | | | | *Mean (SD): $23.25* | | | | | | |  |  |  | *Outpatients* | | *Mean (SD): $0.99* | |  |
|  |  |  |  |  |  |  |  |  | *Median (IQR): $0.00* | | | | | | | | | |  |  |  |  |  |  |  |  |  |  |  | *Median (IQR): $19.85* | | | | | | |  |  |  |  |  | *Median (IQR): $0.00* | |  |
|  |  |  |  |  |  |  | *Intensive Phase* | | *Mean (SD): $0.99* | | | | | | | | | |  |  |  |  |  |  |  | *Intensive Phase* | | | | *Mean (SD): $7.70* | | | | | | |  |  |  | *Intensive Phase* | | *Mean (SD): $4.35* | |  |
|  |  |  |  |  |  |  |  |  | *Median (IQR): $0.00* | | | | | | | | | |  |  |  |  |  |  |  |  |  |  |  | *Median (IQR): $1.98* | | | | | | |  |  |  |  |  | *Median (IQR): $0.00* | |  |
|  |  |  |  |  |  |  | *Continuation Phase* | | *Mean (SD): $4.96* | | | | | | | | | |  |  |  |  |  |  |  | *Continuation Phase* | | | | *Mean (SD): $20.79* | | | | | | |  |  |  | *Continuation Phase* | | *Mean (SD): $1.37* | |  |
|  |  |  |  |  |  |  |  |  | *Median (IQR): $0.00* | | | | | | | | | |  |  |  |  |  |  |  |  |  |  |  | *Median (IQR): $16.54* | | | | | | |  |  |  |  |  | *Median (IQR): $0.00* | |  |
| *Razzaq, 2022* | *Diagnostics* | | | | | *Median (IQR): $4.20 (3.00 – 12.10)* |  | | | | | | | | | | | |  |  | | | | | |  | | | | | |  | | | | |  | | |  | |  | | |
|  | *Intensive Phase* | | | | | *Median (IQR): $3.10 (1.70 – 5.50)* |  | | | | | | | | | | | |  |  | | | | | |  | | | | | |  | | | | |  | | |  | |  | | |
|  | *Continuation Phase* | | | | | *Median (IQR): $3.50 (1.30 – 7.80)* |  | | | | | | | | | | | |  |  | | | | | |  | | | | | |  | | | | |  | | |  | |  | | |
| *Rupani, 2020^49^* | *Private Provider* | *Median (IQR): $4.00 (2-5)* | | | | | *DOT* | | *Private Provider* | | *Median (IQR): $0.00 (0-0)* | | | *Private Provider* | | | | | *Medina (IQR): $0.00 (0-0)* | *Private Provider* | | | | | *Median (IQR): $0.00 (0-0)* | | | |  | | | | | | | | | |  | | | |  |  |
|  |  |  |  |  |  |  |  |  | *Public Provider* | | *Median (IQR): $0.00 (0-0)* | | |  |  |  |  |  |  |  |  |  |  |  |  |  |  |  |  |  |  |  |  |  |  |  |  |  |  |  |  |  |  |  |
|  | *Public Provider* | *Median (IQR): $3.00 (2-4)* | | | | |  |  | *Total* | | *Median (IQR): $0.00 (0-0)* | | | *Public Provider* | | | | | *Median (IQR): $0.00 (0-0)* | *Public Provider* | | | | | *Median (IQR): $0.00 (0-0)* | | | |  |  |  |  |  |  |  |  |  |  |  |  |  |  |  |  |
|  |  |  |  |  |  |  | *Travel to attend health facility* | | *Private Provider* | | *Median (IQR): $4.00 (2-5)* | | |  |  |  |  |  |  |  |  |  |  |  |  |  |  |  |  |  |  |  |  |  |  |  |  |  |  |  |  |  |  |  |
|  | *Total* | *Median (IQr): $3.00 (2-4)* | | | | |  |  | *Public Provider* | | *Median (IQR): $3.00 (2-4)* | | | *Total* | | | | | *Median (IQR): $0.00 (0-0)* | *Total* | | | | | *Median (IQR): 0.00 (0-0)* | | | |  |  |  |  |  |  |  |  |  |  |  |  |  |  |  |  |
|  |  |  |  |  |  |  |  |  | *Total* | | *Median (IQR): $3.00 (2 – 4)* | | |  |  |  |  |  |  |  |  |  |  |  |  |  |  |  |  |  |  |  |  |  |  |  |  |  |  |  |  |  |  |  |
| *Rupani, 2022* | *TB* | *Median (IQR): $29.00 (20.00 – 53.00)* | | | | | *TB* | | *Median (IQR): $29.00 (20.00 – 52.00)* | | | | | | | | | |  |  | | | | | |  | | | | | | | | | | |  | | | | | |  |  |
|  | *TB/HIV* | *Median (IQR): $47.00 (30.00 – 76.00)* | | | | | *TB/HIV* | | *Median (IQR): $47.00 (30.00 – 76.00)* | | | | | | | | | |  |  |  |  |  |  |  |  |  |  |  |  |  |  |  |  |  |  |  |  |  |  |  |  |  |  |
| *Shin, 2020^50^* |  | | | | | | *Inpatient (Initial)* | | Mean (SD):  $3.19 | | | | | *Inpatient (Initial)* | | - | | | *Inpatient (Initial)* | Mean (SD):  $6.55 | | | | | |  | | | | | | | | | | *Childcare* | *Inpatient (Initial)* | | | *Mean (SD):*  $2.03 | | | |  |
|  |  |  |  |  |  |  | *Inpatient (Recurrent)* | | Mean (SD):  $2.06 | | | | | *Inpatient (Recurrent)* | | - | | | *Inpatient (Recurrent)* | Mean (SD):  $10.18 | | | | | |  |  |  |  |  |  |  |  |  |  |  | *Inpatient (Recurrent)* | | | *Mean (SD):*  $3.68 | | | |  |
|  |  |  |  |  |  |  | *Outpatient (HIV)* | | *Mean (SD):*  $0.25 | | | | | *Outpatient (HIV)* | | Mean (SD): $0.61 | | | *Outpatient (HIV)* | Mean (SD):  $0.40 | | | | | |  |  |  |  |  |  |  |  |  |  |  | *Outpatient (HIV)* | | | *Mean (SD):*  $0.29 | | | |  |
|  |  |  |  |  |  |  | *Outpatient (TB)* | | Mean (SD):  $0.26 | | | | | *Outpatient (TB)* | | Mean (SD):  $0.39 | | | *Outpatient (TB)* | Mean (SD):  $0.51 | | | | | |  |  |  |  |  |  |  |  |  |  |  | *Outpatient (TB)* | | | *Mean (SD):*  $0.18 | | | |  |
| *Stracker, 2019^51^* |  | | | | | |  | | | | | | |  | | | | |  | | | | | | |  | | | | | | | *Non-transport OOP costs* | | | | *TB+* | | | *Mean: $8.98* | | | | |
|  |  |  |  |  |  |  |  |  |  |  |  |  |  |  |  |  |  |  |  |  |  |  |  |  |  |  |  |  |  |  |  |  |  |  |  |  | *Xpert-* | | | *Mean: $0.82* | | | | |
| *Sweeney, 2018*^52^* | *Mean: $23.18* | | | | | |  | | | | | | |  | | | | |  | | | | | | |  | | | | | | | | | | |  | | | | | |  |  |
| *Timire, 2021^53^* |  | | | | | | *DS-TB* | | | *Median (IQR): $26.12* | | | | *DS-TB* | | | *Median (IQR) $0.00 (0-0)* | | *DS-TB* | *Median (IQR): $20.41* | | | | *DS-TB* | | *Median (IQR): $293.89* | | | |  | | | | | | | | | | | | |  |  |
|  |  |  |  |  |  |  | *DR-TB* | | | *Median (IQR): $124.09* | | | | *DR-TB* | | | *Median (IQR): $0.00 (0-0)* | | *DR-TB* | *Median (IQR): $81.64* | | | | *DR-TB* | | *Median (IQR): $783.72* | | | |  |  |  |  |  |  |  |  |  |  |  |  |  |  |  |
|  |  |  |  |  |  |  | *Total* | | | *Median (IQR): $26.12* | | | | *Total* | | | *Median (IQR): $0.00 (0-0_* | | *Total* | *Median (IQR): $20.41* | | | | *Total* | | *Median (IQR): $293.89* | | | |  |  |  |  |  |  |  |  |  |  |  |  |  |  |  |
| *Tomeny, 2020*^54^* |  | | | | | | DS-TB | | | Mean: $0.78 | | | | DS-TB | | | *Mean:*  $0.04 | | DS-TB | *Mean:*  $0.07 | | | | DS-TB | | *Mean:*  $3.64 | | | |  | | | | | | | | | | | | |  |  |
|  |  |  |  |  |  |  | MDR-TB | | | Mean:  $17.23 | | | | MDR-TB | | | *Mean:*  $1.30 | | MDR-TB | *Mean:*  $2.60 | | | | MDR-TB | | *Mean:*  $12.77 | | | |  |  |  |  |  |  |  |  |  |  |  |  |  |  |  |
| *Trajman, 2016** |  | | | | | | Minimum Wage | | | Mean (SD):  $2.62 | | | |  | | | | | Minimum Wage | Mean (SD):  $0.65 | | | | Minimum Wage | | Mean (SD):  $26.83 | | | | *Non-Transport & Food Services* | | | | | | | *Minimum Wage* | | | *Mean (SD):*  $0.98 | | | | |
|  |  |  |  |  |  |  | Reported Income | | | Mean (SD):  $1.64 | | | |  |  |  |  |  | Reported Income | Mean (SD):  $0.65 | | | | Reported Income | | Mean (SD):  $34.35 | | | |  |  |  |  |  |  |  | *Reported Income* | | | *Mean (SD):*  $0.16 | | | | |
| *Ukwaja, 2013^(1)56^* |  | | | | | | *Mean: $14.82* | | | | | | |  | | | | | *Mean: $2.65* | | | | | | |  | | | | | | | | | | |  | | | | | |  |  |
| *Viney, 2019^59^* | *Mean (95% CI): $1,889.77* | | | | | | *Mean (95% CI): $511.52* | | | | | | | *Mean (95% CI): $19.95* | | | | | *Mean (95% CI): $1358.29* | | | | | | |  | | | | | | | | | | |  | | | | | |  |  |
| *Viney, 2022* | Extra-pulmonary TB | | Median (IQR): $501.00 (212.00 – 1,323.00) | | | | Extra-pulmonary TB | | | | | *Median (IQR): $76.00 (65.00 – 167.00)* | | Extra-pulmonary TB | *Median (IQR): $58.00 (0 – 72.00)* | | | | Extra-pulmonary TB | *Median (IQR): $75.00 (59.00 – 160.00)* | | | | | | Extra-pulmonary TB | *Median (IQR): $320.00 (0 – 880.00)* | | | | | | | | | | |  | | | | | |  |
|  | Pulmonary TB | | Median (IQR): $395.00 (122.00 – 826.00) | | | | Pulmonary TB | | | | | *Median (IQR): $30.00 (10.00 – 107.00)* | | Pulmonary TB | *Median (IQR): $31.00 (0 – 59.00)* | | | | Pulmonary TB | *Median (IQR): $49.00 (11.00 – 134.00)* | | | | | | Pulmonary TB | *Median (IQR): $64.00 (0 – 427.00)* | | | | | | | | | | |  |  |  |  |  |  |  |
|  | Total | | Median (IQR): $435.00 (146.00 – 961.00) | | | | Total | | | | | *Median (IQR): $46.00 (13.00 – 123.00)* | | Total | *Median (IQR): $32.00 (0 – 65.00)* | | | | Total | *Median (IQR): $64.00 (15.00 – 142.00)* | | | | | | Total | *Median (IQR): $85.00 (0 – 427.00)* | | | | | | | | | | |  |  |  |  |  |  |  |
| *Vo, 2021* | ACF | | Mean (95% CI): $44 (31-57) | | | |  | | | | | | |  | | | | |  | | | | | | |  | | | | | | | | | | |  | | | | | |  |  |
|  | PCF | | Median (IQR): $27 (12-59) | | | |  |  |  |  |  |  |  |  |  |  |  |  |  |  |  |  |  |  |  |  |  |  |  |  |  |  |  |  |  |  |  |  |  |  |  |  |  |  |
|  | Total | | Mean (95% CI): $43 (14-71) | | | |  |  |  |  |  |  |  |  |  |  |  |  |  |  |  |  |  |  |  |  |  |  |  |  |  |  |  |  |  |  |  |  |  |  |  |  |  |  |
| *Wang, 2020^61^* | *Mean: $505.48* | | | | | |  | | | | | | |  | | | | |  | | | | | | |  | | | | | | | | | | |  | | | | | |  |  |
|  | *Median (IQR): $460.40* | | | | | |  |  |  |  |  |  |  |  |  |  |  |  |  |  |  |  |  |  |  |  |  |  |  |  |  |  |  |  |  |  |  |  |  |  |  |  |  |  |
| *Abbreviations: TB – Tuberculosis, DS-TB – Drug sensitive TB, MDR-TB – Multi-drug resistant TB, SSM – Sputum smear microscopy, LJ – Löwenstein Jensen solid culture, LPA – Line probe assay, DR-TB – Drug resistant TB, RS-TB – Rifampicin sensitive TB, RMR-TB – Rifampicin mono-resistant TB, HIV – Human Immunodeficiency Virus, LTBI – Latent TB Infection, CHC – Community health centre, ACF – Active case finding, PCF – Passive case finding, SES – Socioeconomic status, DOT – Directly observed therapy, SD – Standard deviation, IQR – Interquartile range, CI – Confidence Interval*  **Costs reported are a combination of pre- and post-diagnostic costs* | | | | | | | | | | | | | | | | | | | | | | | | | | | | | | | | | | | | | | | | | | |  |  |
