# Supplementary material for: The catastrophic cost of TB care: Understanding costs incurred by individuals undergoing TB care in low-, middle-, and high-income settings – A systematic review
Source: PLOS Glob Public Health. 2025 Apr 2;5(4):e0004283. doi: 10.1371/journal.pgph.0004283 (PMC12005564; doi:10.1371/journal.pgph.0004283)
Supplement: S7 Table — (DOCX) [file pgph.0004283.s013.docx]

## ***Table S7 – Breakdown of the indirect costs incurred by patients during the post-diagnostic phase of TB care***

|  | *Total* | | | | | | | | *Loss of Income* | | | | | | | *Time/Productivity Loss* | | *Caregiver/Guardian Costs* | | | | | | | | | | | | | | *Other* | | | |
| --- | --- | --- | --- | --- | --- | --- | --- | --- | --- | --- | --- | --- | --- | --- | --- | --- | --- | --- | --- | --- | --- | --- | --- | --- | --- | --- | --- | --- | --- | --- | --- | --- | --- | --- | --- |
| *Aia, 2022* | DS-TB | | | | | *Mean (95% CI): $375 (316.90 – 433.80)* | | | DS-TB | | | *Mean (95% CI): $375 (316.90 – 433.80)* | | | |  | |  | | | | | | | | | | | | | |  | | | |
|  | MDR-TB | | | | | *Mean (95% CI): $1,603 (263.90 – 3,469.90)* | | | MDR-TB | | | *Mean (95% CI): $1,603 (263.90 – 3,469.90)* | | | |  |  |  |  |  |  |  |  |  |  |  |  |  |  |  |  |  |  |  |  |
|  | Total | | | | | *Mean (95% CI): $399 (332.10 – 465.20)* | | | Total | | | *Mean (95% CI): $399 (332.10 – 465.20)* | | | |  |  |  |  |  |  |  |  |  |  |  |  |  |  |  |  |  |  |  |  |
| *Assebe, 2020^18^* |  | | | | | | | | *Outpatient* | | | Mean (SD) - $11.82 (27.94) | | | |  | |  | | | | | | | | | | | | | |  | | | |
|  |  |  |  |  |  |  |  |  |  |  |  | Median (IQR) - $0.00 (0-4.30) | | | |  |  |  |  |  |  |  |  |  |  |  |  |  |  |  |  |  |  |  |  |
|  |  |  |  |  |  |  |  |  | *Inpatient* | | | Mean (SD) - $13.23 (33.31) | | | |  |  |  |  |  |  |  |  |  |  |  |  |  |  |  |  |  |  |  |  |
|  |  |  |  |  |  |  |  |  |  |  |  | Median (IQR) - $0.00 | | | |  |  |  |  |  |  |  |  |  |  |  |  |  |  |  |  |  |  |  |  |
|  |  |  |  |  |  |  |  |  | *Total* | | | Mean (SD) - $10.75 (27.94) | | | |  |  |  |  |  |  |  |  |  |  |  |  |  |  |  |  |  |  |  |  |
|  |  |  |  |  |  |  |  |  |  |  |  | Median (IQR) - $0.00 (0-3.22) | | | |  |  |  |  |  |  |  |  |  |  |  |  |  |  |  |  |  |  |  |  |
| *Aung, 2021^19^* |  | | | | | | | |  | | | | | | |  | | *MDR-TB* | | | *Median (min-max): $506.36* | | | | | | | | | | |  | | | |
|  |  |  |  |  |  |  |  |  |  |  |  |  |  |  |  |  |  | *DS-TB* | | | *Median (min-max): $0* | | | | | | | | | | |  |  |  |  |
|  |  |  |  |  |  |  |  |  |  |  |  |  |  |  |  |  |  | *Total* | | | *Median (min-max): $0* | | | | | | | | | | |  |  |  |  |
| *Chandra, 2021^(2)22^* | *Median (IQR): $0 (0 – 105)* | | | | | | | |  | | | | | | |  | |  | | | | | | | | | | | | | |  | | | |
|  | *Mean (SD): $114 (230)* | | | | | | | |  |  |  |  |  |  |  |  |  |  |  |  |  |  |  |  |  |  |  |  |  |  |  |  |  |  |  |
| *Chatterjee, 2023* |  | |  | | | | | | *Intensive Phase* | | *Consultation* | | | | | *Mean:*  *$38.12* | |  | | | | | | | | | | | | | |  | | | |
|  |  |  |  |  |  |  |  |  |  |  | *Medication* | | | | | *Mean:*  *$774.90* | |  |  |  |  |  |  |  |  |  |  |  |  |  |  |  |  |  |  |
|  |  |  |  |  |  |  |  |  | *Continuation Phase* | | *Consultation* | | | | | *Mean:*  *$450.02* | |  |  |  |  |  |  |  |  |  |  |  |  |  |  |  |  |  |  |
|  |  |  |  |  |  |  |  |  |  |  | *Hospitalization* | | | | | *Mean:*  *$291.94* | |  |  |  |  |  |  |  |  |  |  |  |  |  |  |  |  |  |  |
|  |  |  |  |  |  |  |  |  |  |  | *Medication* | | | | | *Mean: $*  *$1,119.02* | |  |  |  |  |  |  |  |  |  |  |  |  |  |  |  |  |  |  |
|  |  |  |  |  |  |  |  |  | *Post-Treatment Phase* | | *Consultation* | | | | | *Mean: $*  *$326.41* | |  |  |  |  |  |  |  |  |  |  |  |  |  |  |  |  |  |  |
|  |  |  |  |  |  |  |  |  |  |  | *Hospitalization* | | | | | *Mean:*  *$204.11* | |  |  |  |  |  |  |  |  |  |  |  |  |  |  |  |  |  |  |
|  |  |  |  |  |  |  |  |  |  |  | *Medication* | | | | | *Mean:*  *$108.79* | |  |  |  |  |  |  |  |  |  |  |  |  |  |  |  |  |  |  |
| *Chittamany, 2020^23^* | *DS-TB* | | *Median (IQR): $50.90* | | | | | |  | | | | | | |  | |  | | | | | | | | | | | | | |  | | | |
|  | *DR-TB* | | *Median (IQR): $2,106.19* | | | | | |  |  |  |  |  |  |  |  |  |  |  |  |  |  |  |  |  |  |  |  |  |  |  |  |  |  |  |
|  | *Total* | | *Median (IQR): $90.67* | | | | | |  |  |  |  |  |  |  |  |  |  |  |  |  |  |  |  |  |  |  |  |  |  |  |  |  |  |  |
| *Diallo, 2022* | DS-TB | | Mean (95% CI): $752.6 (595.3 - 909.9) | | | | | |  | | | | | | |  | |  | | | | | | | | | | | | | |  | | | |
|  | DR-TB | | Mean (95% CI): $459.7 (14.75 - 904.7) | | | | | |  | | | | | | |  | |  | | | | | | | | | | | | | |  | | | |
|  | Total | | Mean (95% CI): $747.6 (592.9 - 902.2) | | | | | |  | | | | | | |  | |  | | | | | | | | | | | | | |  | | | |
| *De Siqueria Filha, 2018^25^* | *TB/HIV* | | *Mean: $212.80* | | | | | | *TB/HIV* | | | | | | *Mean: $143.41* |  | | *TB/HIV* | | | *Mean: $69.38* | | | | | | | | | | |  | | | |
|  | *LTBI/HIV* | | *Mean: $12.45* | | | | | | *LTBI/HIV* | | | | | | *Mean: $0* |  | | *LTBI/HIV* | | | *Mean: $12.45* | | | | | | | | | | |  |  |  |  |
| *Devoid, 2022* |  | |  | | | | | | *Intensive Phase* | | | | | | *Mean (SD): $15.30 (48.91)* |  | | *Intensive Phase* | | | *Mean (SD): $1.96 (12.26)* | | | | | | | | | | |  | | | |
|  |  |  |  |  |  |  |  |  | *Continuation Phase* | | | | | | *Mean (SD): $15.73 (37.78)* |  |  | *Continuation Phase* | | | *Mean (SD): $0.36 (1.71)* | | | | | | | | | | |  |  |  |  |
| *Ellaban, 2021^26^* | *First two months of treatment (intensive phase)* | | *Median (IQR_: $0.00 (0.00 – 93.80)* | | | | | |  | | | | | | |  | |  | | | | | | | | | | | | | |  | | | |
|  | *Second two months of treatment* | | *Median (IQR): $0.00 (0.00 – 75.0)* | | | | | |  |  |  |  |  |  |  |  |  |  |  |  |  |  |  |  |  |  |  |  |  |  |  |  |  |  |  |
|  | *Third two months of treatment* | | *Median (IQR): $0.00 (0.00 - 62.50)* | | | | | |  |  |  |  |  |  |  |  |  |  |  |  |  |  |  |  |  |  |  |  |  |  |  |  |  |  |  |
| *Florentino. 2022* | *Urban DS-TB* | | *Mean (SD): $330.10 (987.40)* | | | | | |  | | | | | | |  | |  | | | | | | | | | | | | | |  | | | |
|  | *Rural DS-TB* | | *Mean (SD): $210.00 (553.30)* | | | | | |  |  |  |  |  |  |  |  |  |  |  |  |  |  |  |  |  |  |  |  |  |  |  |  |  |  |  |
|  | *DR-TB* | | *Mean (SD): $1,078.50 (3,000.70)* | | | | | |  |  |  |  |  |  |  |  |  |  |  |  |  |  |  |  |  |  |  |  |  |  |  |  |  |  |  |
|  | *Total* | | *Mean (SD): $242.90 (765.50)* | | | | | |  |  |  |  |  |  |  |  |  |  |  |  |  |  |  |  |  |  |  |  |  |  |  |  |  |  |  |
| *Fuady, 2018^29^* | *DS-TB* | | *Median (IQR): $2.75 (0.00 – 154.16)* | | | | | | *Patient* | | *DS-TB* | | *Median (IQR): $0.69 (0.00 – 154.16)* | | |  | |  | | | | | | | | | | | | | |  | | | |
|  |  |  |  |  |  |  |  |  |  |  | *MDR-TB* | | *Median (IQR): $462.48 (0.69- 886.77)* | | |  |  |  |  |  |  |  |  |  |  |  |  |  |  |  |  |  |  |  |  |
|  |  |  |  |  |  |  |  |  | *Guardian* | | *DS-TB* | | *Median (IQR): $0.00 (0-0)* | | |  |  |  |  |  |  |  |  |  |  |  |  |  |  |  |  |  |  |  |  |
|  |  |  |  |  |  |  |  |  |  |  | *MDR-TB* | | *Median (IQR): $0.00 (0-0)* | | |  |  |  |  |  |  |  |  |  |  |  |  |  |  |  |  |  |  |  |  |
|  | *MDR-TB* | | *Median (IQR): $462.48 (0.69- 886.77)* | | | | | | *Total* | | *DS-TB* | | *Median (IQR): $40.26* | | |  |  |  |  |  |  |  |  |  |  |  |  |  |  |  |  |  |  |  |  |
|  |  |  |  |  |  |  |  |  |  |  | *MDR-TB* | | *Median (IQR): $949.74* | | |  |  |  |  |  |  |  |  |  |  |  |  |  |  |  |  |  |  |  |  |
| *Getahun, 2016^30^* | *Mean (SD): $63.21* | | | | | | | | *Mean (SD): $47.81* | | | | | | |  | | *Mean (SD): $1.74* | | | | | | | | | | | | | | *Hospital Stay* | *Mean (SD): $19.08* | | |
|  | *Median (R): $51.97* | | | | | | | | *Median (R): $35.54* | | | | | | |  |  | *Median (R): $1.12* | | | | | | | | | | | | | |  | *Median (R): $14.91* | | |
| *Gospodarevskaya, 2014^31^* |  | | | | | | | | *Tanzania* | *Patients* | *First two months of treatment* | | | | *Mean: $6.38* |  | | *Tanzania* | | | *First two months of treatment* | | | | *Mean: $1.58* | | | | | | |  | | | |
|  |  |  |  |  |  |  |  |  |  |  | *Most recent two months of treatment* | | | | *Mean: $4.61* |  |  |  |  |  |  |  |  |  |  |  |  |  |  |  |  |  |  |  |  |
|  |  |  |  |  |  |  |  |  |  |  | *Total treatment* | | | | *Mean: $15.61* |  |  |  |  |  | *Most recent two months of treatment* | | | | *Mean: $1.72* | | | | | | |  |  |  |  |
|  |  |  |  |  |  |  |  |  |  | *Caregivers* | *First two months of treatment* | | | | *Mean: $0.66* |  |  |  |  |  |  |  |  |  |  |  |  |  |  |  |  |  |  |  |  |
|  |  |  |  |  |  |  |  |  |  |  | *Most recent two months of treatment* | | | | *Mean: $0.45* |  |  |  |  |  |  |  |  |  |  |  |  |  |  |  |  |  |  |  |  |
|  |  |  |  |  |  |  |  |  |  |  | *Total treatment* | | | | *Mean: $1.56* |  |  |  |  |  | *Total treatment* | | | | *Mean: $5.02* | | | | | | |  |  |  |  |
|  |  |  |  |  |  |  |  |  | *Bangladesh* | *Patients* | *First two months of treatment* | | | | *Mean: $12.41* |  |  | *Bangladesh* | | | *First two months of treatment* | | | | *Mean: $2.16* | | | | | | |  |  |  |  |
|  |  |  |  |  |  |  |  |  |  |  | *Most recent two months of treatment* | | | | *Mean: $9.65* |  |  |  |  |  |  |  |  |  |  |  |  |  |  |  |  |  |  |  |  |
|  |  |  |  |  |  |  |  |  |  |  | *Total treatment* | | | | *Mean: $31.71* |  |  |  |  |  | *Most recent two months of treatment* | | | | *Mean: $1.14* | | | | | | |  |  |  |  |
|  |  |  |  |  |  |  |  |  |  | *Caregivers* | *First two months of treatment* | | | | *Mean; $0.36* |  |  |  |  |  |  |  |  |  |  |  |  |  |  |  |  |  |  |  |  |
|  |  |  |  |  |  |  |  |  |  |  | *Most recent two months of treatment* | | | | *Mean: $0.18* |  |  |  |  |  |  |  |  |  |  |  |  |  |  |  |  |  |  |  |  |
|  |  |  |  |  |  |  |  |  |  |  | *Total treatment* | | | | *Mean: $0.72* |  |  |  |  |  | *Total treatment* | | | | *Mean: $4.44* | | | | | | |  |  |  |  |
| *Gurung, 2019^33^* | *ACF* | | *Median (IQR): $59.58* | | | | | | *ACF* | | *Median (IQR): $19.64* | | | | | *ACF* | | *Median (IQR): $32.45* | | |  | | | | | | | | | | |  | | | |
|  | *PCF* | | *Median (IQR): $64.68* | | | | | | *PCF* | | *Median (IQR): $10.42* | | | | | *PCF* | | *Median (IQR): $33.64* | | |  |  |  |  |  |  |  |  |  |  |  |  |  |  |  |
|  | *Total* | | *Median (IQR): $59.80* | | | | | | *Total* | | *Median (IQR): $18.56* | | | | | *Total* | | *Median (IQR): $32.45* | | |  |  |  |  |  |  |  |  |  |  |  |  |  |  |  |
| *Gurung, 2021^32^* | *ACF* | | *Mean (95% CI): $38.70 (33.20 – 44.10)* | | | | | |  | | | | | | |  | | | | |  | | | | | | | | | | |  | | | |
|  |  |  | *Median (IQR): $29.60 (21.20 – 50.10)* | | | | | |  |  |  |  |  |  |  |  |  |  |  |  |  |  |  |  |  |  |  |  |  |  |  |  |  |  |  |
|  | *PCF* | | *Mean (95% CI): $44.10 (33.40 – 54.80)* | | | | | |  |  |  |  |  |  |  |  |  |  |  |  |  |  |  |  |  |  |  |  |  |  |  |  |  |  |  |
|  |  |  | *Median (IQR): $27.90 (17.00 – 51.50)* | | | | | |  |  |  |  |  |  |  |  |  |  |  |  |  |  |  |  |  |  |  |  |  |  |  |  |  |  |  |
|  | *Total* | | *Mean (95% CI): $41.40 (35.40 – 47.30)* | | | | | |  |  |  |  |  |  |  |  |  |  |  |  |  |  |  |  |  |  |  |  |  |  |  |  |  |  |  |
|  |  |  | *Median (IQR): $29.10 (19.50 – 50.20)* | | | | | |  |  |  |  |  |  |  |  |  |  |  |  |  |  |  |  |  |  |  |  |  |  |  |  |  |  |  |
| *Kaswa, 2021* |  | | | | | | | | DS-TB | | | | | | *Mean (95% CI): $157.00 (106.20 – 208.60)* |  | | | | |  | | | | | | | | | | |  | | | |
|  |  |  |  |  |  |  |  |  | DR-TB | | | | | | *Mean (95% CI): $533.00 (320.00 – 746.60)* |  |  |  |  |  |  |  |  |  |  |  |  |  |  |  |  |  |  |  |  |
|  |  |  |  |  |  |  |  |  | Total | | | | | | *Mean (95% CI): $549.00 (427.40 – 669.80)* |  |  |  |  |  |  |  |  |  |  |  |  |  |  |  |  |  |  |  |  |
| *Kilale, 2022* | *Mean (SD): $79.70 (635.80)* | | | | | | | |  | | | | | | |  | | | | |  | | | | | | | | | | |  | | | |
|  | *Median (IQR): $16.80 (6.30 – 38.20)* | | | | | | | |  |  |  |  |  |  |  |  |  |  |  |  |  |  |  |  |  |  |  |  |  |  |  |  |  |  |  |
| *Kirubi, 2021^34^* | *Median (IQR): $35.55* | | | | | | | | *Median (IQR): $28.64* | | | | | | |  | | | | |  | | | | | | | | | | |  | | | |
| *Loureiro, 2024* |  | | | | | | | | *Mean: $1,119.87* | | | | | | |  | | | | | *Mean: $387.28* | | | | | | | | | | |  | | | |
| *Lu, 2020^35^* |  | | | | | | | | *Residents* | | *Mean: $1270.44* | | | | |  | | | | | *Care-seeking* | *Residents* | | | *Mean: $71.65* | | | | | | |  | | | |
|  |  |  |  |  |  |  |  |  | *Migrants* | | *Mean: $715.56* | | | | |  |  |  |  |  |  | *Migrants* | | | *Mean: $32.68* | | | | | | |  |  |  |  |
| *Mauch, 2013^(1)^ ^36^* | *Ghana* | | *Mean: $1.76* | | | | | |  | | | | | | |  | | | | |  | | | | | | | | | | |  | | | |
|  |  |  | *Median (IQR): $0.00* | | | | | |  |  |  |  |  |  |  |  |  |  |  |  |  |  |  |  |  |  |  |  |  |  |  |  |  |  |  |
|  | *Vietnam* | | *Mean: $5.84* | | | | | |  |  |  |  |  |  |  |  |  |  |  |  |  |  |  |  |  |  |  |  |  |  |  |  |  |  |  |
|  |  |  | *Median (IQR): $1.57* | | | | | |  |  |  |  |  |  |  |  |  |  |  |  |  |  |  |  |  |  |  |  |  |  |  |  |  |  |  |
|  | *Dominican Republic* | | *Mean: $13.86* | | | | | |  |  |  |  |  |  |  |  |  |  |  |  |  |  |  |  |  |  |  |  |  |  |  |  |  |  |  |
|  |  |  | *Median (IQR): $11.25* | | | | | |  |  |  |  |  |  |  |  |  |  |  |  |  |  |  |  |  |  |  |  |  |  |  |  |  |  |  |
| *Mauch, 2013^(2) *38^* | New | | *Median: $654.98* | | | | | |  | | | | | | |  | | | | | New | | | | | | | | *Median: $105.50* | | |  | | | |
|  | Retreatment | | *Median: $199.18* | | | | | |  |  |  |  |  |  |  |  |  |  |  |  | Retreatment | | | | | | | | *Median: $65.82* | | |  |  |  |  |
|  | MDR-TB | | *Median: $2,836.59* | | | | | |  |  |  |  |  |  |  |  |  |  |  |  | MDR-TB | | | | | | | | *Median: $158.70* | | |  |  |  |  |
| *McAllister, 2020^39^* | *CHC* | | *Median (IQR): $102.69* | | | | | | *CHC* | | | | | *Median (IQR): $108.93* | |  | | | | |  | | | | | | | | | | |  | | | |
|  | *Public Hospital* | | *Median (IQR): $169.81* | | | | | | *Public Hospital* | | | | | *Median (IQR): $179.36* | |  |  |  |  |  |  |  |  |  |  |  |  |  |  |  |  |  |  |  |  |
|  | *Private Hospital* | | *Median (IQR): $233.39* | | | | | | *Private Hospital* | | | | | *Median (IQR): $133.25* | |  |  |  |  |  |  |  |  |  |  |  |  |  |  |  |  |  |  |  |  |
|  | *Private Practice* | | *Median (IQR): $198.81* | | | | | | *Private Practice* | | | | | *Median (IQR): $70.93* | |  |  |  |  |  |  |  |  |  |  |  |  |  |  |  |  |  |  |  |  |
| *Morishita, 2016^40^* |  | | | | | | | | *Patient* | *ACF* | *Mean (SD): $67.55* | | | | |  | | | | |  | | | | | | | | | | |  | | | |
|  |  |  |  |  |  |  |  |  |  |  | *Median (IQR): $0.00* | | | | |  |  |  |  |  |  |  |  |  |  |  |  |  |  |  |  |  |  |  |  |
|  |  |  |  |  |  |  |  |  |  | *PCF* | *Mean (SD): $98.70* | | | | |  |  |  |  |  |  |  |  |  |  |  |  |  |  |  |  |  |  |  |  |
|  |  |  |  |  |  |  |  |  |  |  | *Median (IQR): $0.00* | | | | |  |  |  |  |  |  |  |  |  |  |  |  |  |  |  |  |  |  |  |  |
|  |  |  |  |  |  |  |  |  | *Guardian* | *ACF* | *Mean (SD): $28.60* | | | | | | |  | | |  | | | | | | | | | | |  | | | |
|  |  |  |  |  |  |  |  |  |  |  | *Median (IQR): $0.00* | | | | | | |  |  |  |  |  |  |  |  |  |  |  |  |  |  |  |  |  |  |
|  |  |  |  |  |  |  |  |  |  | *PCF* | *Mean (SD): $14.02* | | | | | | |  |  |  |  |  |  |  |  |  |  |  |  |  |  |  |  |  |  |
|  |  |  |  |  |  |  |  |  |  |  | *Median (IQR): $0.00* | | | | | | |  |  |  |  |  |  |  |  |  |  |  |  |  |  |  |  |  |  |
|  | *TB/HIV* | | *Mean (SD): $*  25.17 | | | | | | *Job Loss* | *TB/HIV* | *Mean (SD): $*7.41 | | | | | | |  | | | *Study Clinic* | | *TB/HIV* | | *Mean (SD): $*  0.54 | | | | | | |  | | | |
|  |  |  |  |  |  |  |  |  |  | *TB* | *Mean (SD): $*8.55 | | | | | | |  |  |  |  |  | *TB* | | *Mean (SD): $*  0.11 | | | | | | |  |  |  |  |
|  | *TB* | | *Mean (SD): $*28.12 | | | | | |  | *HIV* | *Mean (SD): $*1.44 | | | | | | |  |  |  |  |  | *HIV* | | *Mean (SD): $*  1.88 | | | | | | |  |  |  |  |
|  |  |  |  |  |  |  |  |  | *Care Seeking* | *TB/HIV* | *Mean (SD): $*14.64 | | | | | | |  | | | *Other Facilities* | | *TB/HIV* | | *Mean (SD): $*  0.45 | | | | | | |  |  |  |  |
|  | *HIV* | | *Mean (SD): $*10.64 | | | | | |  | *TB* | *Mean (SD): $*16.64 | | | | | | |  |  |  |  |  | *TB* | | *Mean (SD): $*  0.02 | | | | | | |  |  |  |  |
|  |  |  |  |  |  |  |  |  |  | *HIV* | *Mean (SD): $*6.64 | | | | | | |  |  |  |  |  | *HIV* | | *Mean (SD): $*  0.11 | | | | | | |  |  |  |  |
| *Muniyandi, 2020^42^* | *Mean (SD): $320.68 (677.17)* | | | | | | | | *Mean (SD): $272.73 (576.89)* | | | | | | | | |  | | | *Mean (SD): $41.88 (354.65)* | | | | | | | | | | | *Mean (SD): $5.11 (40.04)* | | | |
|  | *Median (IQR): $0.00 (0.00 – 6114.57)* | | | | | | | | *Median (IQR): $0.00 (0.00 – 4323.95)* | | | | | | | | |  |  |  | *Median (IQR): $0.00 (0.00.-5412.90)* | | | | | | | | | | | *Median (IQR): $0.00 (0.00 – 701.67)* | | | |
| *Muttamba, 2020^43^* | *MDR-TB* | | *Mean (95% CI): $488.11* | | | | | |  | | | | | | | | |  | | | *MDR-TB* | | | | | | *Mean (95% CI): $46.05* | | | | |  | | | |
|  | *DS-TB* | | *Mean (95% CI): $46.05* | | | | | |  |  |  |  |  |  |  |  |  |  |  |  | *DS-TB* | | | | | | *Mean (95% CI): $10.09* | | | | |  |  |  |  |
|  | *Total* | | *Mean (95% CI): $62.47* | | | | | |  |  |  |  |  |  |  |  |  |  |  |  | *Total* | | | | | | *Mean (95% CI): $11.17* | | | | |  |  |  |  |
| *Nhung, 2018^*44^* | *MDR-TB* | | *Mean (95% CI); $6,044.51* | | | | | | *MDR-TB* | | | | | | *Mean (95% CI); $6044.51* | | |  | | |  | | | | | | | | | | |  | | | |
|  | *DS-TB* | | *Mean (95% CI): $2,231.55* | | | | | | *DS-TB* | | | | | | *Mean (95% CI): $2231.55* | | |  |  |  |  |  |  |  |  |  |  |  |  |  |  |  |  |  |  |
|  | *Total* | | *Mean (95% CI): $2,534.65* | | | | | | *Total* | | | | | | *Mean (95% CI): $2534.65* | | |  |  |  |  |  |  |  |  |  |  |  |  |  |  |  |  |  |  |
| *Pedrazzoli, 2018^45^* |  | | | | | | | | *MDR-TB* | | | | | | Median (IQR): $0.00 | | |  | | | *MDR-TB* | | | Median (IQR): $0.00 | | | | | | | |  | | | |
|  |  |  |  |  |  |  |  |  | *DS-TB* | | | | | | Median (IQR): $0.00 | | |  |  |  | *DS-TB* | | | Median (IQR): $0.00 | | | | | | | |  |  |  |  |
|  |  |  |  |  |  |  |  |  | *Total* | | | | | | Median (IQR): $0.00 | | |  |  |  | *Total* | | | Median (IQR): $0.00 | | | | | | | |  |  |  |  |
| *Pedrazzoli, 2021^46^* |  | | | | | | | | *Uninsured* | | | | | | *Mean (SD): $93.90* | | |  | | |  | | | | | | | | | | |  | | | |
|  |  |  |  |  |  |  |  |  |  |  |  |  |  |  | *Median (IQR): $0.00* | | |  |  |  |  |  |  |  |  |  |  |  |  |  |  |  |  |  |  |
|  |  |  |  |  |  |  |  |  | *Insured* | | | | | | *Mean (SD): $97.55* | | |  |  |  |  |  |  |  |  |  |  |  |  |  |  |  |  |  |  |
|  |  |  |  |  |  |  |  |  |  |  |  |  |  |  | *Median (IQR): $0.00* | | |  |  |  |  |  |  |  |  |  |  |  |  |  |  |  |  |  |  |
| *Pham, 2023* |  | | | | | | | | *MDR-TB* | | | | | | *Median (IQR): $179.76 (75.08 – 333.27)* | | |  | | |  | | | | | | | | | | | *Ambulatory Care Visits* | | | *Median (IQR): $115.84 (63.00 - 175.23)* |
| *Prasanna, 2018^47^* | *Study Population* | | Median (IQR): $  61.90 | | | | | | *Income Change* | | | | | | *Study Population* | | Median (IQR): $  0.00 | *Study Population* | | Median (IQR): $  80.51 | *Study Population* | | | | | Median (IQR): $  0.00 | | | | | |  | | | |
|  |  |  |  |  |  |  |  |  |  |  |  |  |  |  | *Those who incurred costs* | | Median (IQR): $  82.61 |  |  |  |  |  |  |  |  |  |  |  |  |  |  |  |  |  |  |
|  | *Those who incurred costs* | | Median (IQR): $  164.36 | | | | | | *Job Loss* | | | | | | *Study Population* | | Median (IQR): $  0.00 | *Those who incurred costs* | | Median (IQR): $  84.34 | *Those who incurred costs* | | | | | Median (IQR): $  0.00 | | | | | |  |  |  |  |
|  |  |  |  |  |  |  |  |  |  |  |  |  |  |  | *Those who incurred costs* | | Median (IQR): $412.93 |  |  |  |  |  |  |  |  |  |  |  |  |  |  |  |  |  |  |
| *Ramma, 2015^48^* | *Inpatients* | | *Mean (SD): $114.79* | | | | | |  | | | | | | |  | | *Seeking Care* | *Inpatients* | *Mean (SD): $5.15* |  | | | |  | | | *Inpatients* | | | *Mean (SD): $3.17* |  | | | |
|  |  |  |  |  |  |  |  |  |  |  |  |  |  |  |  |  |  |  |  | *Median (IQR): $0.43* |  | | | |  |  |  |  |  |  |  |  |  |  |  |
|  |  |  |  |  |  |  |  |  |  |  |  |  |  |  |  |  |  |  | *Outpatients* | *Mean (SD): $3.92* |  | | | |  |  |  |  |  |  | *Median (IQR): $0.99* |  |  |  |  |
|  |  |  |  |  |  |  |  |  |  |  |  |  |  |  |  |  |  |  |  | *Median (IQR): $1.37* |  | | | |  |  |  |  |  |  |  |  |  |  |  |
|  | *Outpatients* | | *Mean (SD): $29.86* | | | | | |  |  |  |  |  |  |  |  |  |  | *Intensive Phase* | *Mean (SD): $3.64* |  | | | |  | | | *Outpatients* | | | *Mean (SD); $13.33* |  |  |  |  |
|  |  |  |  |  |  |  |  |  |  |  |  |  |  |  |  |  |  |  |  | *Median (IQR): $0.00* |  | | | |  |  |  |  |  |  |  |  |  |  |  |
|  |  |  |  |  |  |  |  |  |  |  |  |  |  |  |  |  |  |  | *Continuation Phase* | *Mean (SD): $1.94* |  | | | |  |  |  |  |  |  | *Median (IQR): $0.00* |  |  |  |  |
|  |  |  |  |  |  |  |  |  |  |  |  |  |  |  |  |  |  |  |  | *Median (IQR): $0.61* |  | | | |  |  |  |  |  |  |  |  |  |  |  |
|  | *Intensive Phase* | | *Mean (SD); $91.44* | | | | | |  |  |  |  |  |  |  | *Hospitalization* | | *Inpatients* | | | *Mean (SD): $106.42* | | | | *Intensive Phase* | | | | | | *Mean (SD): $5.95* |  |  |  |  |
|  |  |  |  |  |  |  |  |  |  |  |  |  |  |  |  |  |  |  |  |  | *Median (IQR): $70.41* | | | |  |  |  |  |  |  |  |  |  |  |  |
|  |  |  |  |  |  |  |  |  |  |  |  |  |  |  |  |  |  | *Outpatients* | | | *Mean (SD): $12.61* | | | |  |  |  |  |  |  | *Median (IQR): $0.85* |  |  |  |  |
|  |  |  |  |  |  |  |  |  |  |  |  |  |  |  |  |  |  |  |  |  | *Median (IQR): $0.00* | | | |  |  |  |  |  |  |  |  |  |  |  |
|  | *Continuation Phase* | | *Mean (SD): $60.01* | | | | | |  |  |  |  |  |  |  |  |  | *Intensive Phase* | | | *Mean (SD): $81.85* | | | | *Continuation Phase* | | | | | | *Mean (SD): $15.55* |  |  |  |  |
|  |  |  |  |  |  |  |  |  |  |  |  |  |  |  |  |  |  |  |  |  | *Median (IQR): $54.63* | | | |  |  |  |  |  |  |  |  |  |  |  |
|  |  |  |  |  |  |  |  |  |  |  |  |  |  |  |  |  |  | *Continuation Phase* | | | *Mean (SD): $42.53* | | | |  |  |  |  |  |  | *Median (IQR): $0.00* |  |  |  |  |
|  |  |  |  |  |  |  |  |  |  |  |  |  |  |  |  |  |  |  |  |  | *Median (IQR): $0.00* | | | |  |  |  |  |  |  |  |  |  |  |  |
| *Rupani, 2020^49^* | *Private Provider* | | *Median (IQR): $20.00 (3-76)* | | | | | | *Private Provider* | | | | | | *Median (IQR): $8.00 (0-21)* |  | | *Private Provider* | | | *Median (IQR): $0.00 (0-19)* | | | |  | | | | | | | *Private Provider* | *Median (IQR): $0.00 (0-27)* | | |
|  | *Public Provider* | | *Median (IQR): $4.00 (3-10)* | | | | | | *Public Provider* | | | | | | *Median (IQR): $3.00 (0-6)* |  |  | *Public Provider* | | | *Median (IQR): $0.00 (0-3)* | | | |  |  |  |  |  |  |  | *Public Provider* | *Median (IQR): $0.00 (0-0)* | | |
|  | *Total* | | *Median (IQR): $6.00 (3-13)* | | | | | | *Total* | | | | | | *Median (IQR): $3.00 (0-6)* |  |  | *Total* | | | *Median (IQR): $0.00 (0-3)* | | | |  | | | | | | | *Total* | *Median (IQR): $0.00 (0-0)* | | |
| *Rupani, 2022* | *TB* | | *Median (IQR): $18.00 (10.00 – 32.00)* | | | | | | *TB* | | | | | | *Median (IQR): $12.00 (5.00 – 19.00)* |  | | *TB* | | | *Median (IQR): $4.00 (2.00 – 7.00)* | | | |  | | | | | | |  | | | |
|  | *TB/HIV* | | *Median (IQR): $33.00 (18.00 – 54.00)* | | | | | | *TB/HIV* | | | | | | *Median (IQR): $20.00 (10.00 – 35.00)* |  | | *TB/HIV* | | | *Median (IQR): $9.00 (4.00 – 13.00)* | | | |  | | | | | | |  | | | |
| *Shin, 2020^50^* | Inpatient (Initial) | | Mean (SD):  $21.34 | | | | | | Inpatient (Initial) | | | | | | Mean (SD):  $14.46 |  | | Inpatient (Initial) | | | *Mean (SD):* $10.97 | | | |  | | | | | | |  | | | |
|  | Inpatient (Recurrent) | | Mean (SD):  $66.71 | | | | | | Inpatient (Recurrent) | | | | | | Mean (SD):  $56.69 |  |  | Inpatient (Recurrent) | | | *Mean (SD):* $13.47 | | | |  | | | | | | |  |  |  |  |
|  | Outpatient (HIV) | | *Mean (SD):*  $1.26 | | | | | | Outpatient (HIV) | | | | | | Mean (SD):  $1.26 |  |  | Outpatient (HIV) | | | *-* | | | |  | | | | | | |  |  |  |  |
|  | Outpatient (TB) | | *Mean (SD):*  $1.43 | | | | | | Outpatient (TB) | | | | | | Mean (SD):  $1.43 |  |  | Outpatient (TB) | | | *-* | | | |  | | | | | | |  |  |  |  |
| *Stracker, 2019^51^* |  | | | | | | | | *TB+* | | | | | | *Mean: $186.40* |  | | *TB+* | | | *Mean: $8.98* | | | |  | | | | | | |  | | | |
|  |  |  |  |  |  |  |  |  | *Xpert-* | | | | | | *Mean: $2.45* |  |  | *Xpert-* | | | *Mean: $1.63* | | | |  | | | | | | |  |  |  |  |
| *Sweeney, 2018*^52^* | *Approach #1: Current income (prompted ranges)* | | | | *Mean (SD):*  $21.12 | | | |  | | | | | | |  | |  | | |  | | | | | | | | | | |  | | | |
|  | *Approach #2: Current income (detailed)* | | | | *Mean (SD):*  $27.59 | | | |  |  |  |  |  |  |  |  |  |  |  |  |  |  |  |  |  |  |  |  |  |  |  |  |  |  |  |
|  | *Approach #3: Permanent income (MCA)* | | | | *Mean (SD):*  $47.36 | | | |  |  |  |  |  |  |  |  |  |  |  |  |  |  |  |  |  |  |  |  |  |  |  |  |  |  |  |
|  | *Approach #4: National mean income* | | | | *Mean (SD):*  $72.08 | | | |  |  |  |  |  |  |  |  |  |  |  |  |  |  |  |  |  |  |  |  |  |  |  |  |  |  |  |
|  | *Approach #5: Self-reported income loss* | | | | *Mean (SD):*  $54.45 | | | |  |  |  |  |  |  |  |  |  |  |  |  |  |  |  |  |  |  |  |  |  |  |  |  |  |  |  |
| *Timire, 2021*^53^* | *DS-TB* | | | | *Median (IQR): $195.93* | | | |  | | | | | | |  | |  | | |  | | | | | | | | | | |  | | | |
|  | *DR-TB* | | | | *Median (IQR): $979.66* | | | |  |  |  |  |  |  |  |  |  |  |  |  |  |  |  |  |  |  |  |  |  |  |  |  |  |  |  |
|  | *Total* | | | | *Median (IQR): $244.92* | | | |  |  |  |  |  |  |  |  |  |  |  |  |  |  |  |  |  |  |  |  |  |  |  |  |  |  |  |
| *Tomeny, 2020*^54^* | DS-TB | | | | *Mean:*  $4.94 | | | |  | | | | | | |  | |  | | |  | | | | | | | | | | |  | | | |
|  | MDR-TB | | | | *Mean:*  $51.46 | | | |  |  |  |  |  |  |  |  |  |  |  |  |  |  |  |  |  |  |  |  |  |  |  |  |  |  |  |
| *Trajman, 2016*^55^* | All | *Minimum Wage* | | | | | Mean (SD):  $103.38 | |  | | | | | | |  | | *Minimum Wage* | | | *Mean (SD):*  $6.87 | | | | *Minimum Wage* | | | | | *Mean (SD);* $6.54 | |  | | | |
|  |  | *SES Income per Capita* | | | | | Mean (SD):  $103.38 | |  |  |  |  |  |  |  |  |  |  |  |  |  |  |  |  |  |  |  |  |  |  |  |  |  |  |  |
|  |  | *Income per Activity* | | | | | *Mean (SD):* $108.62 | |  |  |  |  |  |  |  |  |  | *SES Income per Capita* | | | *Mean (SD):*  $5.89 | | | | *SES Income per Capita* | | | | | *Mean (SD):* $7.20 | |  |  |  |  |
|  |  | *Reported Income* | | | | | Mean (SD): $116.80 | |  |  |  |  |  |  |  |  |  |  |  |  |  |  |  |  |  |  |  |  |  |  |  |  |  |  |  |
|  | *Patients Only* | *Minimum Wage* | | | | | *Mean (SD):*  $101.42 | |  |  |  |  |  |  |  | *Income per Activity* | | *Mean (SD):*  $9.81 | | |  | | | | *Income per Activity* | | | | | *Mean (SD):*$8.83 | |  |  |  |  |
|  |  | *SES Income per Capita* | | | | | *Mean (SD):*  $102.40 | |  |  |  |  |  |  |  |  |  |  |  |  |  |  |  |  |  |  |  |  |  |  |  |  |  |  |  |
|  |  | *Income per Activity* | | | | | Mean (SD):  $105.67 | |  |  |  |  |  |  |  | *Reported Income* | | *Mean (SD):*  $8.18 | | |  | | | | *Reported Income* | | | | | *Mean (SD):*$1.64 | |  |  |  |  |
|  |  | *Reported Income* | | | | | Mean (SD)  $115.82 | |  |  |  |  |  |  |  |  |  |  |  |  |  |  |  |  |  |  |  |  |  |  |  |  |  |  |  |
| *Ukwaja, 2013^(1)56^* | *Mean: $42.86* | | | | | | | | *Mean: $42.33* | | | | | | |  | | | | | *Median: $33.87* | | | |  | | | | | | | *Value of time for drug collections* | | *Mean: $0.80* | |
| *Ukwaja, 2013^(2)57^* |  | | | | | | | |  | | | | | | |  | | | | |  | | | |  | | | | | | | *DOT supporter costs* | | *Mean: $3.70* | |
| *Van der Hof, 2016^58^* |  | | DS-TB | | | | | MDR-TB |  | | | | | | |  | | | | |  | | | |  | | | | | | |  | | | |
|  | Ethiopia | Intensive Phase | *Median (IQR): $0.00* | | | | | *Median (IQR): $275.02* |  |  |  |  |  |  |  |  |  |  |  |  |  |  |  |  |  |  |  |  |  |  |  |  |  |  |  |
|  |  | Continuation Phase | *Median (IQR): $0.00* | | | | | *Median (IQR): $91.26* |  |  |  |  |  |  |  |  |  |  |  |  |  |  |  |  |  |  |  |  |  |  |  |  |  |  |  |
|  | Indonesia | Intensive Phase | *Median (IQR): $3.24* | | | | | *Median (IQR): $101.90* |  |  |  |  |  |  |  |  |  |  |  |  |  |  |  |  |  |  |  |  |  |  |  |  |  |  |  |
|  |  | Continuation Phase | *Median (IQR): $2.91* | | | | | *Median (IQR): $82.17* |  |  |  |  |  |  |  |  |  |  |  |  |  |  |  |  |  |  |  |  |  |  |  |  |  |  |  |
|  | Kazakhstan | Intensive Phase | *Median (IQR): $143.96* | | | | | *Median (IQR): $547.71* |  |  |  |  |  |  |  |  |  |  |  |  |  |  |  |  |  |  |  |  |  |  |  |  |  |  |  |
|  |  | Continuation Phase | *Median (IQR): $37.06* | | | | | *Median (IQR): $80.89* |  |  |  |  |  |  |  |  |  |  |  |  |  |  |  |  |  |  |  |  |  |  |  |  |  |  |  |
| *Viney, 2019^59^* | *Mean (95% CI): $1,392.81* | | | | | | | |  | | | | | | |  | | | | |  | | | | | | | | | | |  | | | |
| *Viney, 2022* | Extra-pulmonary TB | | | *Median (IQR): $99.00 (0 – 258.00* | | | | |  | | | | | | |  | |  | | |  | | | | | | | | | | |  | | | |
|  | Pulmonary TB | | | *Median (IQR); $74.00 (0 – 333.00)* | | | | |  |  |  |  |  |  |  |  |  |  |  |  |  |  |  |  |  |  |  |  |  |  |  |  |  |  |  |
|  | Total | | | *Median (IQR): $74.00 (0 – 296.00)* | | | | |  |  |  |  |  |  |  |  |  |  |  |  |  |  |  |  |  |  |  |  |  |  |  |  |  |  |  |
| *Vo, 2021* | ACF | | | | | | | Mean (95% CI): $13 (7-20) |  | | | | | | |  | |  | | |  | | | | | | | | | | |  | | | |
|  |  |  |  |  |  |  |  | Median (IQR): $6 (4-13) |  | | | | | | |  | |  | | |  | | | | | | | | | | |  | | | |
|  | PCF | | | | | | | Mean (95% CI): $27 (1-53) |  | | | | | | |  | |  | | |  | | | | | | | | | | |  | | | |
|  |  |  |  |  |  |  |  | Median (IQR): $5 (4-8) |  | | | | | | |  | |  | | |  | | | | | | | | | | |  | | | |
|  | Total | | | | | | | Mean (95% CI): $20 (7-33) |  | | | | | | |  | |  | | |  | | | | | | | | | | |  | | | |
|  |  |  |  |  |  |  |  | Median (IQR): $5 (4-11) |  | | | | | | |  | |  | | |  | | | | | | | | | | |  | | | |
| *Wang, 2020^61^* | *Mean: $2,579.84* | | | | | | | |  | | | | | | |  | |  | | |  | | | | | | | | | | |  | | | |
|  | *Median (IQR): $1,575.69* | | | | | | | |  |  |  |  |  |  |  |  |  |  |  |  |  |  |  |  |  |  |  |  |  |  |  |  |  |  |  |
| *Abbreviations: TB – Tuberculosis, DS-TB – Drug sensitive TB, DR-TB – Drug resistant TB, MDR-TB – Multidrug resistant TB, RS-TB – Rifampicin sensitive TB, RMR-TB – Rifampicin mono-resistant TB, HIV – Human Immunodeficiency Virus, LTBI – Latent TB Infection, ACF – Active case finding, PCF – Passive case finding, SES – Socioeconomic status, DOT – Directly observed therapy, SD – Standard deviation, IQR – Interquartile range, CI – Confidence Interval*  **Costs reported are a combination of pre- and post-diagnostic costs* | | | | | | | | | | | | | | | | | | | | | | | | | | | | | | | | | | | |
