# Supplementary material for: The catastrophic cost of TB care: Understanding costs incurred by individuals undergoing TB care in low-, middle-, and high-income settings – A systematic review
Source: PLOS Glob Public Health. 2025 Apr 2;5(4):e0004283. doi: 10.1371/journal.pgph.0004283 (PMC12005564; doi:10.1371/journal.pgph.0004283)
Supplement: S8 Table — (DOCX) [file pgph.0004283.s014.docx]

## ***Table S8 - Use of Coping Strategies in the Included Studies:***

|  | *N* | | | | *Used Coping Strategies* | | *Borrowed Money/Loans* | | *Sold Assets* | | | | | | *Use of Savings* | | *Family Assistance* | | *Combination of Mechanisms* | |
| --- | --- | --- | --- | --- | --- | --- | --- | --- | --- | --- | --- | --- | --- | --- | --- | --- | --- | --- | --- | --- |
| *Aia, 2022^14^* | *1000* | | | | *26.4%* | | *DS-TB* | *22.6%* | *DS-TB* | *8.1%* | | | | |  | |  | | *DS-TB* | *26.9%* |
|  |  |  |  |  |  |  | *MDR-TB* | *0%* | *MDR-TB* | *0%* | | | | |  |  |  |  | *MDR-TB* | *0%* |
|  |  |  |  |  |  |  | *Total* | *22.2%* | *Total* | *7.9%* | | | | |  |  |  |  | *Total* | *26.4%* |
| *Assebe, 2020^15^* | *787* | | | | *68%* | | *4%* | | *19%* | | | | | |  | | *16%* | |  | |
| *Aung, 2021^16^* | *967* | | | |  | | *32.5%* | | *23.8%* | | | | | | *36.8%* | |  | | *64.4%* | |
| *Chandra, 2021(1)^21^* | *110* | | | | *46.36%* | | *94%* | |  | | | | | |  | |  | |  | |
| *Chatterjee, 2023* | *221** | | | |  | | *Intensive Phase* | *47.06%* | *Intensive Phase* | | | | *11.31%* | | *Intensive Phase* | *20.81%* |  | |  | |
|  |  |  |  |  |  |  | *Continuation Phase* | *28.51%* | *Continuation Phase* | | | | *12.67%* | | *Continuation Phase* | *-* |  |  |  |  |
|  |  |  |  |  |  |  | *Post-Treatment* | *26.24%* | *Post-Treatment* | | | | *13.12%* | | *Post-Treatment* | *-* |  |  |  |  |
| *Chittamany, 2020^23^* | *DS-TB* | | | *717* |  | | *26.4%* | | *17.7%* | | | | | | *21.2%* | |  | | 49.9% | |
|  | *DR-TB* | | | *8* |  | | *25%* | | *12.5%* | | | | | | *37.5%* | |  | | 50% | |
|  | *Total* | | | *725* |  | | *26.3%* | | *17.7%* | | | | | | *21.4%* | |  | | 49.9% | |
| *Collins, 2018^24^* | *169* | | | |  | | *41%* | | *Sold Property: 38%* | | | | | |  | |  | |  | |
|  |  |  |  |  |  |  |  |  | *Leased Property: 7%* | | | | | |  |  |  |  |  |  |
| *Diallo, 2022* |  | | | | *DS-TB* | *39.8%* | *DS-TB* | *25.8%* | *DS-TB* | | *24.6%* | | | |  | |  | |  | |
|  |  |  |  |  | *DR-TB* | *-* | *DR-TB* | *-* | *DR-TB* | | *-* | | | |  |  |  |  |  |  |
|  |  |  |  |  | *Total* | *39.8%* | *Total* | *26.1%* | *Total* | | *24.0%* | | | |  |  |  |  |  |  |
| *Devoid, 2022* | *244* | | | | *Before treatment* | *57%* | *Before treatment* | *9%* | *Before treatment* | | *7%* | | | |  | |  | |  | |
|  |  |  |  |  | *Intensive Phase* | *18%* | *Intensive Phase* | *5%* | *Intensive Phase* | | *2%* | | | |  |  |  |  |  |  |
|  |  |  |  |  | *Continuation Phase* | *6%* | *Continuation Phase* | *3%* | *Continuation Phase* | | *1%* | | | |  |  |  |  |  |  |
| *Ellaban, 2021^26^* | *151* | | | |  | | *67.6%* | | *33.1%* | | | | | |  | |  | |  | |
|  |  |  |  |  |  | | *Friends* | 56.3% | *Household Property* | | | 45.7% | | |  | |  | |  | |
|  |  |  |  |  |  | | *Family* | 39.4% | *Jewelry & Gold* | | | 45.7% | | |  | |  | |  | |
|  |  |  |  |  |  | | *Employers* | 3.2% | *Means of Transport* | | | 8.7% | | |  | |  | |  | |
|  |  |  |  |  |  | | *Other* | 1.1% |  |  |  |  |  |  |  | |  | |  | |
| *Florentino, 2022* | *Urban DS-TB* | | *786* | |  | | *Urban DS-TB* | *25.6%* | *Urban DS-TB* | | | *4.7%* | | | *Urban DS-TB* | *12.7%* |  | | *Urban DS-TB* | *34.5%* |
|  | *Rural DS-TB* | | *806* | |  |  | *Rural DS-TB* | *32.9%* | *Rural DS-TB* | | | *8.4%* | | | *Rural DS-TB* | *14.0%* |  |  | *Rural DS-TB* | *44.7%* |
|  | *DR-TB* | | *320* | |  |  | *DR-TB* | *34.7%* | *DR-TB* | | | *14.7%* | | | *DR-TB* | *11.6%* |  |  | *DR-TB* | *44.4%* |
| *Gadallah, 2022* | *257* | | | | *11.3%* | | *30.6%* | | *26.4%* | | | | | |  | |  | | 49.0% | |
| *Getahun, 2016^30^* | *576* | | | |  | | 18% | | 11% | | | | | |  | |  | |  | |
|  |  |  |  |  |  | | Neighbours | 34% |  |  |  |  |  |  |  | |  | |  | |
|  |  |  |  |  |  | | Family | 23% |  |  |  |  |  |  |  | |  | |  | |
|  |  |  |  |  |  | | Friends | 26% |  |  |  |  |  |  |  | |  | |  | |
|  |  |  |  |  |  | | Organizations | 17% |  |  |  |  |  |  |  | |  | |  | |
| *Gurung, 2019^33^* | *ACF* | | | *50* |  | | *28%* | | *8%* | | | | | |  | |  | |  | |
|  | *PCF* | | | *49* |  | | *44%* | | *10%* | | | | | |  | |  | |  | |
|  | *Total* | | | *99* |  | | *36%* | | *9%* | | | | | |  | |  | |  | |
| *Gurung, 2021^32^* | *ACF* | | | *111* |  | |  | |  | | | | | |  | |  | |  | |
|  | *PCF* | | | *110* |  |  |  |  |  |  |  |  |  |  |  |  |  |  |  |  |
| *Kaswa, 2021* | *1,118* | | | *49%* |  | |  | |  | | | | | |  | |  | |  | |
| *Kilale, 2022* | *777* | | | *53%* |  | |  | |  | | | | | |  | |  | |  | |
| *Manyazewal, 2022* | *109* | | | *0%* |  | |  | |  | | | | | |  | |  | |  | |
| *Mauch, 2013 (1)^36^* | *Ghana* | | | *135* |  | | *47%* | | *37%* | | | | | |  | |  | |  | |
|  | *Vietnam* | | | *258* |  | | *17%* | | *5%* | | | | | |  | |  | |  | |
|  | *Dominican Republic* | | | *150* |  | | *45%* | | *19%* | | | | | |  | |  | |  | |
| *Mauch, 2013 (2)^38^* | *198* | | | |  | | *45%* | | *Property* | | | | | *20%* |  | |  | |  | |
|  |  |  |  |  |  | |  |  | *Household Items* | | | | | *43%* |  | |  | |  | |
|  |  |  |  |  |  | |  |  | *Vehicles* | | | | | *14%* |  | |  | |  | |
|  |  |  |  |  |  | |  |  | *Land* | | | | | *8%* |  | |  | |  | |
|  |  |  |  |  |  | |  |  | *House* | | | | | *3%* |  | |  | |  | |
| *Mauch, 2011^37^* | *208* | | | |  | | *57%* | | *52%* | | | | | |  | |  | |  | |
| *McAllister, 2020^39^* | *469* | | | |  | | *31%* | |  | | | | | |  | |  | |  | |
| *Medeiros, 2024* | *65* | | | | *73.8%* | |  | |  | | | | | |  | |  | |  | |
| *Morishita, 2016^40^* | *ACF* | | | *108* | *46.30%* | | *42.60%* | | *13.90%* | | | | | |  | |  | |  | |
|  | *PCF* | | | *100* | *52%* | | *46%* | | *21%* | | | | | |  | |  | |  | |
| *Muttamba, 2020^42^* | *DS-TB* | | | *1134* |  | | *25.90%* | |  | | | | | | *10%* | |  | | 47.20% | |
|  | *MDR-TB* | | | *44* |  | | *54.40%* | |  | | | | | | *39.90%* | |  | | 81.20% | |
| *Nhung, 2018^44^* | *735* | | | |  | | *25%* | | *5.8%* | | | | | | *16%* | |  | | 38% | |
| *Pedrazzoli, 2018^45^* | *DS-TB* | | | *625* | *51%* | | *27%* | | *10.7%* | | | | | | *..4%* | |  | | 52% | |
|  | *MDR-TB* | | | *66* |  |  | *30.3%* | | *15.2%* | | | | | | *16.7%* | |  | | 47% | |
|  | *Total* | | | *691* |  |  | *27.4%* | | *11.1%* | | | | | | *28.2%* | |  | | 51.5% | |
| *Prasanna, 2018^47^* | *102* | | | |  | | *38%* | | *8%* | | | | | | *20.8%* | |  | |  | |
| *Razzaq, 2022* | *516* | | | | *94%* | | *4.7%* | | *2.9%* | | | | | | *17.2%* | | *1.2%* | |  | |
| *Rupani, 2020^49^* | *458* | | | | *18%* | | *11%* | |  | | | | | |  | |  | |  | |
|  |  |  |  |  |  |  | *Median (IQR) amount borrowed: $71 (29 – 143)* | |  |  |  |  |  |  |  |  |  |  |  |  |
| *Rupani, 2022* | *234* | | | | *34%* | | *5%* | |  | | | | | |  | |  | |  | |
| *Sweeney, 2018^52^* | *66* | | | | *36%* | | *30%* | | *3%* | | | | | |  | |  | | 12% | |
| *Tomeny, 2020^54^* | *194* | | | |  | | *70%* | | *7%* | | | | | | *37%* | |  | |  | |
| *Ukwaja, 2013 (2)^57^* | *452* | | | | *88%* | | *47%* | | *9%* | | | | | |  | |  | | 32% | |
| *Van der Hof, 2016^58^* |  | *DS-TB* | | *MDR-TB* |  | | *DS-TB* | *MDR-TB* | *DS-TB* | | | | | *MDR-TB* |  | | *DS-TB* | *MDR-TB* |  | |
|  | *Ethiopia* | *25* | | *169* |  | | *56%* | *41%* | *24%* | | | | | *38%* |  | | *-* | *-* |  | |
|  | *Indonesia* | *118* | | *143* |  | | *9%* | *27%* | *9%* | | | | | *27%* |  | | *32%* | *43%* |  | |
|  | *Kazakhstan* | *54* | | *94* |  | | *0%* | *4%* | *0%* | | | | | *1%* |  | | *57%* | *66%* |  | |
| *Viney, 2022* | *183* | | | | Extra-pulmonary TB | *13.9%* | Extra-pulmonary TB | *8.3%* | Extra-pulmonary TB | *0%* | | | |  | Extra-pulmonary TB | *5.6%* |  | |  | |
|  |  |  |  |  | Pulmonary TB | *21.1%* | Pulmonary TB | *11.6%* | Pulmonary TB | *4.1%* | | | |  | Pulmonary TB | *10.2%* |  |  |  |  |
|  |  |  |  |  | Total | *19.7%* | Total | *10.9%* | Total | *3.3%* | | | |  | Total | *9.3%* |  |  |  |  |
| *Walcott, 2020^60^* | *196* | | | | *26%* | |  | |  | | | | |  |  | |  | |  | |
| *Abbreviations: TB – Tuberculosis, DS-TB – Drug sensitive TB, DR-TB – Drug resistant TB, MDR-TB – Multi-drug resistant TB, ACF – Active case finding, PCF – Passive case finding, IQR – Interquartile range* | | | | | | | | | | | | | | | | | | | | |
